# Elliptic differential operators on Lipschitz domains and abstract boundary value problems

**DOI:** 10.1016/j.jfa.2014.09.017

**Published:** 2014-11-15

**Authors:** Jussi Behrndt, Till Micheler

**Affiliations:** a Institut für Numerische Mathematik, TU Graz, Steyrergasse 30, 8010 Graz, Austria; b Institut für Mathematik, TU Berlin, Straße des 17. Juni 136, 10623 Berlin, Germany

**Keywords:** Lipschitz domain, Laplacian, Boundary triple, Self-adjoint extension

## Abstract

This paper consists of two parts. In the first part, which is of more abstract nature, the notion of quasi-boundary triples and associated Weyl functions is developed further in such a way that it can be applied to elliptic boundary value problems on non-smooth domains. A key feature is the extension of the boundary maps by continuity to the duals of certain range spaces, which directly leads to a description of all self-adjoint extensions of the underlying symmetric operator with the help of abstract boundary values. In the second part of the paper a complete description is obtained of all self-adjoint realizations of the Laplacian on bounded Lipschitz domains, as well as Kreĭn type resolvent formulas and a spectral characterization in terms of energy dependent Dirichlet-to-Neumann maps. These results can be viewed as the natural generalization of recent results by Gesztesy and Mitrea for quasi-convex domains. In this connection we also characterize the maximal range spaces of the Dirichlet and Neumann trace operators on a bounded Lipschitz domain in terms of the Dirichlet-to-Neumann map. The general results from the first part of the paper are also applied to higher order elliptic operators on smooth domains, and particular attention is paid to the second order case which is illustrated with various examples.

## 1 Introduction

Spectral theory of elliptic partial differential operators has received a lot of attention in the recent past, in particular, modern techniques from abstract operator theory were applied to extension and spectral problems for symmetric and self-adjoint elliptic differential operators on bounded and unbounded domains. We refer the reader to the recent contributions [3], [11], [12], [13], [17], [18], [43], [44], [45], [53] on smooth domains, [1], [4], [5], [33], [34], [35], [40], [42], [61], [62], [64] on non-smooth domains, and we point out the paper [36] by Gesztesy and Mitrea which has inspired parts of the present work. Many of these contributions are based on the classical works Grubb [39] and Višik [72] on the parameterization of the closed realizations of a given elliptic differential expression on a smooth domain, and other classical papers on realizations with local and non-local boundary conditions, see, e.g. [2], [8], [9], [16], [32], [68] and the monograph [52] by Lions and Magenes.

In [36] Gesztesy and Mitrea obtain a complete description of the self-adjoint realizations of the Laplacian on a class of bounded non-smooth, so-called *quasi-convex* domains. The key feature of quasi-convex domains is that the functions in the domains of the self-adjoint Dirichlet realization $\Delta_{D}$ and the self-adjoint Neumann realization $\Delta_{N}$ possess $H^{2}$-regularity, a very convenient property which is well-known to be false for the case of Lipschitz domains; cf. [49]. Denote by $\tau_{D}$ and $\tau_{N}$ the Dirichlet and Neumann trace operator, respectively. Building on earlier work of Maz'ya, Mitrea and Shaposhnikova [55], see also [21], [31], [37], the range spaces $G_{0}:=\tau_{D}(\mathrm{dom}\;\Delta_{N})$ and $G_{1}:=\tau_{N}(\mathrm{dom}\;\Delta_{D})$ were characterized for quasi-convex domains in [36], and the self-adjoint realizations of the Laplacian were parameterized via tuples {X,L}, where X is a closed subspace of the anti-dual $G_{0}^{\prime }$ or $G_{1}^{\prime }$ and *L* is a self-adjoint operator from X to $X^{\prime }$. This parameterization technique has its roots in [15], [51] and was used in [39], [72], see also [41, Chapter 13]. In [17] the connection to the notion of (ordinary) boundary triples from extension theory of symmetric operators was made explicit.

The theory of ordinary boundary triples and Weyl functions originates in the works of Kočubeĭ [50], Bruk [19], Gorbachuk and Gorbachuk [38], and Derkach and Malamud [27], [28]. A boundary triple $\left\{G,\Gamma_{0},\Gamma_{1}\right\}$ for a symmetric operator *A* in a Hilbert space H consists of an auxiliary Hilbert space G and two boundary mappings $\Gamma_{0},\Gamma_{1}:\mathrm{dom}\;A^{⁎}\to G$ which satisfy an abstract Green's identity and a maximality condition. With the help of a boundary triple the closed extensions of the underlying symmetric operator *A* can be parameterized in an efficient way with closed operators and subspaces *Θ* in the boundary space G. The concept of ordinary boundary triples was applied successfully to various problems in extension and spectral theory, in particular, in the context of ordinary differential operators, see [20] for a review and further references. However, for the Laplacian (or more general symmetric elliptic differential operators) on a domain $\Omega \subset R^{n}$, n≥2, with boundary ∂*Ω* the natural choice $\Gamma_{0}=\tau_{D}$ and $\Gamma_{1}=-\tau_{N}$ does not lead to an ordinary boundary triple since Green's identity does not extend to the domain of the maximal operator $A^{⁎}$. This simple observation led to a generalization of the concept of ordinary triples, the so-called *quasi-boundary triples*, which are designed for applications to PDE problems. Here the boundary mappings $\Gamma_{0}=\tau_{D}$ and $\Gamma_{1}=-\tau_{N}$ are only defined on some suitable subset of $\mathrm{dom}\;A^{⁎}$, e.g. $H^{2}(\Omega )$, and the realizations are labeled with operators and subspaces *Θ* in the boundary space $L^{2}(\partial \Omega )$ via boundary conditions of the form $\Theta \tau_{D}f+\tau_{N}f=0$, $f\in H^{2}(\Omega )$. One of the advantages of this approach is that the Weyl function corresponding to the quasi-boundary triple $\left\{L^{2}(\partial \Omega ),\tau_{D},-\tau_{N}\right\}$ coincides (up to a minus sign) with the usual family of Dirichlet-to-Neumann maps on the boundary ∂*Ω*, and hence the spectral properties of a fixed self-adjoint extension can be described with the Dirichlet-to-Neumann map and the parameter *Θ* in the boundary condition.

The aim of the present paper is twofold. Our first objective is to further develop the abstract notion of quasi-boundary triples and their Weyl functions. The main new feature is that we shall assume that the spaces

$$G_{0}=\mathrm{ran}(\Gamma_{0}↾\ker\Gamma_{1})\;\text{and}\;G_{1}=\mathrm{ran}(\Gamma_{1}↾\ker\Gamma_{0})$$

are reflexive Banach spaces densely embedded in the boundary space G; this assumption is natural in the context of PDE problems and related Sobolev spaces on the boundary of the domain, and is satisfied in applications to the Laplacian on Lipschitz domains and other elliptic boundary value problems treated in the second part of the present paper. In fact, this assumption is the abstract analog of the properties of the range spaces in [36], and it is also automatically satisfied in many abstract settings, e.g. for ordinary and so-called generalized boundary triples; cf. [28] and Section 2.4 for a counterexample in the general case. Under the density assumption it then follows that the boundary maps $\Gamma_{0}$ and $\Gamma_{1}$ can be extended by continuity to surjective mappings from $\mathrm{dom}\;A^{⁎}$ onto the anti-duals $G_{1}^{\prime }$ and $G_{0}^{\prime }$, respectively. Then also the *γ*-field and the Weyl function admit continuous extensions to operators mapping in between the appropriate spaces; for the special case of generalized boundary triples and $G_{0}$, $G_{1}$ equipped with particular topologies this was noted in the abstract setting earlier in [28, Proposition 6.3] and [26, Lemma 7.22]. Following the regularization procedure in the PDE case we then show that a quasi-boundary triple with this additional density property can be transformed into a quasi-boundary triple which is the restriction of an ordinary boundary triple, and hence can be extended by continuity; a similar argument can also be found in a different abstract form in [26]. As a consequence of these considerations we obtain a complete description of all closed extensions of the underlying symmetric operator in Section 3, as well as abstract regularity results, Kreĭn type resolvent formulas and new sufficient criteria for the parameter *Θ* in the boundary condition to imply self-adjointness of the corresponding extension.

The second objective of this paper is to apply the abstract quasi-boundary triple technique to various PDE problems. In particular, in Section 4.1 we extend the characterization of the self-adjoint realizations $\Delta_{\Theta }$ of the Laplacian on quasi-convex domains to the more natural case of Lipschitz domains. Here the Hilbert spaces $G_{0}$ and $G_{1}$ are topologized with the help of the Dirichlet-to-Neumann map in a similar manner as in [26], [28] for abstract generalized boundary triples. This also leads to a continuous extension of the Dirichlet and Neumann trace operators on a Lipschitz domain to the maximal domain of the Laplacian, and hence to a description of the Dirichlet boundary data for $L^{2}$-solutions of −Δf=λf. For the special case of quasi-convex domains and $C^{1,r}$-domains with $r\in (\frac{1}{2},1]$ we establish the link to the approach in [36], and recover many of the results in [36] as corollaries of the abstract methods developed in Section 2 and Section 3. In Section 4.2 we illustrate the abstract methods in the classical case of 2*m*-th order elliptic differential operators with smooth coefficients on smooth bounded domains, where the spaces $G_{0}$ and $G_{1}$ coincide with the usual product Sobolev trace spaces on ∂*Ω*. Here, e.g. some classical trace extension results follow from the abstract theory developed in the first part of the paper. Finally, we pay particular attention to the second order case on bounded and unbounded domains with compact smooth boundary in Section 4.3. Here we recover various recent results on the description and the spectral properties of the self-adjoint extensions of a symmetric second order elliptic differential operator, and extend these by adding, e.g. regularity results. This section contains also some simple examples, among them self-adjoint extensions with Robin boundary conditions. One of the examples is also interesting from a more abstract point of view: It turns out that there exist self-adjoint parameters in the range of the boundary maps of a quasi-boundary triple such that the corresponding extension is essentially self-adjoint, but not self-adjoint.

## 2 Quasi-boundary triples and their Weyl functions

The concept of boundary triples and their Weyl functions is a useful and efficient tool in extension and spectral theory of symmetric and self-adjoint operators, it originates in the works [19], [50] and was further developed in [27], [28], [38]; cf. [20] for a review. In the recent past different generalizations of the notion of boundary triples were introduced, among them boundary relations, boundary pairs and boundary triples associated with quadratic forms, and other related concepts, see [7], [24], [25], [26], [59], [60], [63], [64], [66], [67]. The concept of quasi-boundary triples and their Weyl functions introduced in [11] is designed for the analysis of elliptic differential operators. It can be viewed as a slight generalization of the notions of boundary and generalized boundary triples. In this section we first recall some definitions and basic properties which can be found in [11], [12]. Our main objective is to show that under an additional density condition the corresponding boundary maps can be extended by continuity and that the corresponding quasi-boundary triple can be transformed (or regularized) such that it turns into an ordinary boundary triple; cf. [26], [74], [75] for related investigations.

### 2.1 Ordinary and quasi-boundary triples

Let throughout this section *A* be a closed, densely defined, symmetric operator in a separable Hilbert space H.

Definition 2.1 Let $T\subset A^{⁎}$ be a linear operator in H such that $\bar{T}=A^{⁎}$. A triple $\left\{G,\Gamma_{0},\Gamma_{1}\right\}$ is called *quasi-boundary triple* for *T* if G is a Hilbert space and $\Gamma_{0},\Gamma_{1}:\mathrm{dom}\;T\to G$ are linear mappings such that

(i) the *abstract Green's identity*
  (2.1) $${\left(Tf,g\right)}_{H}-{\left(f,Tg\right)}_{H}={\left(\Gamma_{1}f,\Gamma_{0}g\right)}_{G}-{\left(\Gamma_{0}f,\Gamma_{1}g\right)}_{G}$$
  holds for all f,g∈domT,
(ii) the map $\Gamma :={\left(\Gamma_{0},\Gamma_{1}\right)}^{⊤}:\mathrm{dom}\;T\to G\times G$ has dense range,
(iii) and $A_{0}:=T↾\ker\Gamma_{0}$ is a self-adjoint operator in H.

In the special case $T=A^{⁎}$ a quasi-boundary triple $\left\{G,\Gamma_{0},\Gamma_{1}\right\}$ is called *ordinary boundary triple*.

Let $\left\{G,\Gamma_{0},\Gamma_{1}\right\}$ be a quasi-boundary triple for $T\subset A^{⁎}$. Then the mapping $\Gamma ={\left(\Gamma_{0},\Gamma_{1}\right)}^{⊤}:\mathrm{dom}\;T\to G\times G$ is closable with respect to the graph norm of $A^{⁎}$ and ker⁡Γ=domA holds; cf. [11, Proposition 2.2]. Moreover, according to [11, Theorem 2.3] (see also Proposition 2.2 below) we have $T=A^{⁎}$ if and only if ranΓ=G×G, in this case $\Gamma ={\left(\Gamma_{0},\Gamma_{1}\right)}^{⊤}:\mathrm{dom}\;A^{⁎}\to G\times G$ is onto and continuous with respect to the graph norm of $A^{⁎}$, and the restriction $A_{0}=A^{⁎}↾\ker\Gamma_{0}$ is automatically self-adjoint. Thus, the above definition of an ordinary boundary triple coincides with the usual one, see, e.g. [27]. We also note that a quasi-boundary triple is in general not a boundary relation in the sense of [24], [25], but it can be viewed as a certain transform of a boundary relation; cf. [75, Proposition 5.1].

For later purposes we recall a variant of [11, Theorem 2.3].

Proposition 2.2 *Let* G *be a Hilbert space and let T be a linear operator in* H *. Assume that* $\Gamma_{0},\Gamma_{1}:\mathrm{dom}\;T\to G$ *are linear mappings such that the following conditions are satisfied:*

(i) $T↾\ker\Gamma_{0}$ *contains a self-adjoint linear operator A in* H *,*
(ii) *The range and the kernel of* $\Gamma :={\left(\Gamma_{0},\Gamma_{1}\right)}^{⊤}:\mathrm{dom}\;T\to G\times G$ *are dense in* G×G *and* H *, respectively,*
(iii) *The abstract Green's identity* (2.1) *holds for all* f,g∈domT *.*

*Then* S:=T↾ker⁡Γ *is a densely defined, closed symmetric operator in* H *and* $\left\{G,\Gamma_{0},\Gamma_{1}\right\}$ *is a quasi-boundary triple for* $S^{⁎}$ *such that* $A=T↾\ker\Gamma_{0}=A_{0}$ *. Moreover,* $T=S^{⁎}$ *if and only if* ranΓ=G×G *.*

Not surprisingly, suitable restrictions of ordinary boundary triples lead to quasi-boundary triples.

Proposition 2.3 *Let*$\left\{G,\Gamma_{0},\Gamma_{1}\right\}$*be an ordinary boundary triple for*$A^{⁎}$*with*$A_{0}=A^{⁎}↾\ker\Gamma_{0}$*. Let*$T\subset A^{⁎}$*be such that*$A_{0}\subset T$*and*$\bar{T}=A^{⁎}$*. Then the restricted triple*$\left\{G,\Gamma_{0}^{T},\Gamma_{1}^{T}\right\}$*, where*$\Gamma_{0}^{T}:=\Gamma_{0}↾\mathrm{dom}\;T$*and*$\Gamma_{1}^{T}:=\Gamma_{1}↾\mathrm{dom}\;T$*is a quasi-boundary triple for*$T\subset A^{⁎}$*.*

Proof Clearly, items (i) and (iii) in Definition 2.1 hold for the restricted triple $\left\{G,\Gamma_{0}^{T},\Gamma_{1}^{T}\right\}$. Hence it remains to show that $\mathrm{ran}\;\Gamma^{T}=\mathrm{ran}\;{\left(\Gamma_{0}^{T},\Gamma_{1}^{T}\right)}^{⊤}$ is dense in G×G. For this let $\overset{ˆ}{x}\in G\times G$. Then $\overset{ˆ}{x}\in \mathrm{ran}\;\Gamma$ and there exists an element $f\in \mathrm{dom}\;A^{⁎}$ such that $\Gamma f=\overset{ˆ}{x}$. Since $\bar{T}=A^{⁎}$ there exists a sequence $(f_{n})\subset \mathrm{dom}\;T$ which converges to *f* in the graph norm of $A^{⁎}$. As *Γ* is continuous with respect to the graph norm we obtain $\Gamma^{T}f_{n}=\Gamma f_{n}\to \overset{ˆ}{x}$ for n→∞, that is, item (ii) in Definition 2.1 holds and $\left\{G,\Gamma_{0}^{T},\Gamma_{1}^{T}\right\}$ is a quasi-boundary triple for $T\subset A^{⁎}$.  □

The following proposition shows that the converse of Proposition 2.3 holds under an additional continuity assumption. In particular, it implies that if a quasi-boundary triple can be extended to an ordinary boundary triple then this extension is unique.

Proposition 2.4 *Let*$\left\{G,\Gamma_{0}^{T},\Gamma_{1}^{T}\right\}$*be a quasi-boundary triple for*$T\subset A^{⁎}$*. Then*$\left\{G,\Gamma_{0}^{T},\Gamma_{1}^{T}\right\}$*is a restriction of an ordinary boundary triple*$\left\{G,\Gamma_{0},\Gamma_{1}\right\}$*for*$A^{⁎}$*on T if and only if the mapping*$\Gamma^{T}={\left(\Gamma_{0}^{T},\Gamma_{1}^{T}\right)}^{⊤}:\mathrm{dom}\;T\to G\times G$*is continuous with respect to the graph norm of*$A^{⁎}$*.*

Proof (⇒) Since $\Gamma :\mathrm{dom}\;A^{⁎}\to G\times G$ is continuous with respect to the graph norm of $A^{⁎}$ the same holds for the restriction $\Gamma^{T}:\mathrm{dom}\;T\to G\times G$.

(⇐) Let $\Gamma ={\left(\Gamma_{0},\Gamma_{1}\right)}^{⊤}:\mathrm{dom}\;A^{⁎}\to G\times G$ be the continuous extension of $\Gamma^{T}$ with respect to the graph norm of $A^{⁎}$. Then also the abstract Green's identity extends by continuity from dom *T* to $\mathrm{dom}\;A^{⁎}$,

(2.2) $${\left(A^{⁎}f,g\right)}_{H}-{\left(f,A^{⁎}g\right)}_{H}={\left(\Gamma_{1}f,\Gamma_{0}g\right)}_{G}-{\left(\Gamma_{0}f,\Gamma_{1}g\right)}_{G},\;f,g\in \mathrm{dom}\;A^{⁎},$$

and the range of *Γ* is dense in G×G. Moreover, from (2.2) it follows that the operator $A^{⁎}↾\ker\Gamma_{0}$ is a symmetric extension of the self-adjoint operator $A_{0}=T↾\ker\Gamma_{0}^{T}$ and hence $A_{0}=A^{⁎}↾\ker\Gamma_{0}$. We conclude that $\left\{G,\Gamma_{0},\Gamma_{1}\right\}$ is a quasi-boundary triple for $\bar{T}=A^{⁎}$, that is, $\left\{G,\Gamma_{0},\Gamma_{1}\right\}$ is an ordinary boundary triple for $A^{⁎}$; cf. Definition 2.1. Clearly, $\left\{G,\Gamma_{0}^{T},\Gamma_{1}^{T}\right\}$ is the restriction of this ordinary boundary triple to *T*.  □

A simple and useful example of an ordinary and quasi-boundary triple is provided in Lemma 2.5 below, it also implies the well-known fact that a boundary triple or quasi-boundary triple exists if and only if *A* has equal deficiency indices $n_{\pm }(A):=\dim\ker(A^{⁎}\pm i)$, that is, if and only if *A* admits self-adjoint extensions in H. Recall first that for a self-adjoint extension $A_{0}\subset T$ of *A* and $\eta \in \rho (A_{0})$ the domains of *T* and $A^{⁎}$ admit the direct sum decompositions

(2.3) $$\mathrm{dom}\;T=\mathrm{dom}\;A_{0}\;∔\;N_{\eta }(T)\;\text{and}\;\mathrm{dom}\;A^{⁎}=\mathrm{dom}\;A_{0}\;∔\;N_{\eta }\left(A^{⁎}\right),$$

where $N_{\eta }(T)=\ker(T-\eta )$ and $N_{\eta }(A^{⁎})=\ker(A^{⁎}-\eta )$. Note also that $\bar{T}=A^{⁎}$ implies $\bar{N_{\eta }(T)}=N_{\eta }(A^{⁎})$. Moreover we set

$${\overset{ˆ}{N}}_{\eta }(T):=\left\{{\left(f_{\eta },\eta f_{\eta }\right)}^{⊤}:f_{\eta }\in N_{\eta }(T)\right\},\;{\overset{ˆ}{N}}_{\eta }\left(A^{⁎}\right):=\left\{{\left(f_{\eta },\eta f_{\eta }\right)}^{⊤}:f_{\eta }\in N_{\eta }\left(A^{⁎}\right)\right\},$$

hence we may write $T=A_{0}∔{\overset{ˆ}{N}}_{\eta }(T)$ and $A^{⁎}=A_{0}∔{\overset{ˆ}{N}}_{\eta }(A^{⁎})$. The orthogonal projection in H onto the defect subspace $N_{\eta }(A^{⁎})$ will be denoted by $P_{\eta }$.

In the next lemma a special boundary triple and quasi-boundary triple are constructed. The restriction η∈R below is for convenience only, an example of a similar ordinary boundary triple with η∈C∖R can be found in, e.g. [27] or the monographs [38], [69].

Lemma 2.5 *Assume that the deficiency indices of A are equal and let*G*be a Hilbert space with*$\dim G=n_{\pm }(A)$*. Let*$A_{0}$*be a self-adjoint extension of A in*H*, assume that there exists*$\eta \in \rho (A_{0})\cap R$*and fix a unitary operator*$\varphi :N_{\eta }(A^{⁎})\to G$*. Then the following statements hold.*

(i) *The triple*$\left\{G,\Gamma_{0},\Gamma_{1}\right\}$*, where*
  $$\Gamma_{0}f:=\varphi f_{\eta }\;\text{and}\;\Gamma_{1}f:=\varphi P_{\eta }(A_{0}-\eta )f_{0},$$
  *and*$f\in \mathrm{dom}\;A^{⁎}$*is decomposed in*$f=f_{0}+f_{\eta }\in \mathrm{dom}\;A_{0}+N_{\eta }(A^{⁎})$*, is an ordinary boundary triple for*$A^{⁎}$*with*$A_{0}=A^{⁎}↾\ker\Gamma_{0}$*.*
(ii) *If T is an operator such that*$A_{0}\subset T$*and*$\bar{T}=A^{⁎}$*, then the triple*$\left\{G,\Gamma_{0}^{T},\Gamma_{1}^{T}\right\}$*, where*
  $$\Gamma_{0}^{T}f:=\varphi f_{\eta }\;\text{and}\;\Gamma_{1}^{T}f:=\varphi P_{\eta }(A_{0}-\eta )f_{0},$$
  *and*f∈domT*is decomposed in*$f=f_{0}+f_{\eta }\in \mathrm{dom}\;A_{0}+N_{\eta }(T)$*, is a quasi-boundary triple for T with*$A_{0}=T↾\ker\Gamma_{0}^{T}$*and*$\mathrm{ran}\;\Gamma_{1}^{T}=\mathrm{ran}\;\Gamma_{1}=G$*.*

Proof (i) Let $f,g\in \mathrm{dom}\;A^{⁎}$ be decomposed in the form $f=f_{0}+f_{\eta }$ and $g=g_{0}+g_{\eta }$ with $f_{0},g_{0}\in \mathrm{dom}\;A_{0}$ and $f_{\eta },g_{\eta }\in N_{\eta }(A^{⁎})$. Making use of $A_{0}=A_{0}^{⁎}$ and η∈R a straightforward computation yields

$${\left(A^{⁎}f,g\right)}_{H}-{\left(f,A^{⁎}g\right)}_{H}={\left((A_{0}-\eta )f_{0},g_{\eta }\right)}_{H}-{\left(f_{\eta },(A_{0}-\eta )g_{0}\right)}_{H}={\left(\varphi P_{\eta }(A_{0}-\eta )f_{0},\varphi g_{\eta }\right)}_{G}-{\left(\varphi f_{\eta },\varphi P_{\eta }(A_{0}-\eta )g_{0}\right)}_{G}={\left(\Gamma_{1}f,\Gamma_{0}g\right)}_{G}-{\left(\Gamma_{0}f,\Gamma_{1}g\right)}_{G},$$

i.e., the abstract Green's identity holds. Moreover, $\Gamma_{0}:\mathrm{dom}\;A^{⁎}\to G$ is surjective and since $\mathrm{ran}(A_{0}-\eta )=H$ it follows that also $\Gamma :\mathrm{dom}\;A^{⁎}\to G\times G$ is surjective. This implies that $\left\{G,\Gamma_{0},\Gamma_{1}\right\}$ is an ordinary boundary triple for *A*. It is obvious that $A_{0}=A^{⁎}↾\ker\Gamma_{0}$ holds.

(ii) follows from (i) and Proposition 2.3.  □

### 2.2 Weyl functions and *γ*-fields of quasi-boundary triples

In this subsection the notion and some properties of *γ*-fields and Weyl functions associated to quasi-boundary triples are briefly reviewed. Furthermore, a simple but useful description of the range of the boundary mappings is given in terms of the Weyl function in Proposition 2.8.

Let $\left\{G,\Gamma_{0},\Gamma_{1}\right\}$ be a quasi-boundary triple for $T\subset A^{⁎}$ and let $A_{0}=T↾\ker\Gamma_{0}$. Note that by (2.3) the restriction $\Gamma_{0}↾N_{\lambda }(T)$ is invertible for every $\lambda \in \rho (A_{0})$.

Definition 2.6 The *γ-field* and the *Weyl function* corresponding to the quasi-boundary triple $\left\{G,\Gamma_{0},\Gamma_{1}\right\}$ are defined by

$$\lambda \mapsto \gamma (\lambda ):={\left(\Gamma_{0}↾N_{\lambda }(T)\right)}^{-1}\;\text{and}\;\lambda \mapsto M(\lambda ):=\Gamma_{1}\gamma (\lambda ),\;\lambda \in \rho (A_{0}).$$

It follows that for $\lambda \in \rho (A_{0})$ the operator γ(λ) is continuous from G to H with dense domain $\mathrm{dom}\;\gamma (\lambda )=\mathrm{ran}\;\Gamma_{0}$ and range $\mathrm{ran}\;\gamma (\lambda )=N_{\lambda }(T)$, the function λ↦γ(λ)g is holomorphic on $\rho (A_{0})$ for every $g\in \mathrm{ran}\;\Gamma_{0}$, and the relations

(2.4) $$\gamma (\lambda )=\left(I+(\lambda -\mu ){\left(A_{0}-\lambda \right)}^{-1}\right)\gamma (\mu )\;\text{and}\;\gamma {\left(\lambda \right)}^{⁎}=\Gamma_{1}{\left(A_{0}-\overset{¯}{\lambda }\right)}^{-1}$$

hold for all $\lambda ,\mu \in \rho (A_{0})$; cf. [11, Proposition 2.6]. Note that $\gamma {\left(\lambda \right)}^{⁎}:H\to G$ is continuous and that ${\left(\ker\gamma {\left(\lambda \right)}^{⁎}\right)}^{⊥}=\bar{\mathrm{ran}\;\gamma (\lambda )}=N_{\lambda }(A^{⁎})$ yields the orthogonal space decomposition

(2.5) $$H=\ker\gamma {\left(\lambda \right)}^{⁎}⊕N_{\lambda }\left(A^{⁎}\right).$$

For $\lambda \in \rho (A_{0})$ the values M(λ) of the Weyl function are operators in G with dense domain $\mathrm{ran}\;\Gamma_{0}$ and range contained in $\mathrm{ran}\;\Gamma_{1}$. If, in addition, $A_{1}=T↾\ker\Gamma_{1}$ is self-adjoint in H then M(λ) maps $\mathrm{ran}\;\Gamma_{0}$ onto $\mathrm{ran}\;\Gamma_{1}$ for all $\lambda \in \rho (A_{0})\cap \rho (A_{1})$. Furthermore, $M(\lambda )\Gamma_{0}f_{\lambda }=\Gamma_{1}f_{\lambda }$ holds for all $f_{\lambda }\in N_{\lambda }(T)$ and this implies the identity

(2.6) $$\Gamma_{1}f=M(\lambda )\Gamma_{0}f+\Gamma_{1}f_{0},\;f=f_{0}+f_{\lambda }\in \mathrm{dom}\;A_{0}∔N_{\lambda }(T).$$

We also mention that for $\lambda ,\mu \in \rho (A_{0})$ the Weyl function is connected with the *γ*-field via

(2.7) $$M(\lambda )x-M{\left(\mu \right)}^{⁎}x=(\lambda -\overset{¯}{\mu })\gamma {\left(\mu \right)}^{⁎}\gamma (\lambda )x,\;x\in \mathrm{ran}\;\Gamma_{0},$$

and, in particular, M(λ) is a symmetric operator in G for $\lambda \in R\cap \rho (A_{0})$. It is important to note that

(2.8) $$\mathrm{ran}\;\Gamma_{0}=\mathrm{dom}\;M(\lambda )\subset \mathrm{dom}\;M{\left(\mu \right)}^{⁎},\;\lambda ,\mu \in \rho (A_{0}).$$

The subspaces $G_{0}$ and $G_{1}$ of G in the next definition will play a fundamental role throughout this paper.^1^

Definition 2.7 Let $\left\{G,\Gamma_{0},\Gamma_{1}\right\}$ be a quasi-boundary triple for $T\subset A^{⁎}$. Then we define the spaces

$$G_{0}:=\mathrm{ran}(\Gamma_{0}↾\ker\Gamma_{1})\;\text{and}\;G_{1}:=\mathrm{ran}(\Gamma_{1}↾\ker\Gamma_{0}).$$

Observe that for the spaces $G_{0}$ and $G_{1}$ in Definition 2.7 we have $G_{0}\times G_{1}\subset \mathrm{ran}\;\Gamma$. Note also that the second identity in (2.4) implies

(2.9) $$\mathrm{ran}\;\gamma {\left(\lambda \right)}^{⁎}=G_{1},\;\lambda \in \rho (A_{0}).$$

Proposition 2.8 *Let* $\left\{G,\Gamma_{0},\Gamma_{1}\right\}$ *be a quasi-boundary triple for* $T\subset A^{⁎}$ *with* $A_{0}=T↾\ker\Gamma_{0}$ *and Weyl function M, and let* $G_{0}$ *and* $G_{1}$ *be as in* Definition 2.7 *. Then the following assertions hold for all* $\lambda \in \rho (A_{0})$ *.*

(i) M(λ) *maps* $G_{0}$ *into* $G_{1}$ *and if, in addition,* $A_{1}=T↾\ker\Gamma_{1}$ *is self-adjoint, then* $M(\lambda )↾G_{0}$ *is a bijection onto* $G_{1}$ *for* $\lambda \in \rho (A_{0})\cap \rho (A_{1})$ *,*
(ii) *The range of the boundary mapping* $\Gamma ={\left(\Gamma_{0},\Gamma_{1}\right)}^{⊤}$ *is*
  (2.10) $$\mathrm{ran}\;\Gamma =\left\{\left(\begin{array}{c} x\\ x^{\prime } \end{array}\right)\in \mathrm{ran}\;\Gamma_{0}\times \mathrm{ran}\;\Gamma_{1}:x^{\prime }=M(\lambda )x+y,\;y\in G_{1}\right\}$$
  *and, in particular,* $\mathrm{dom}\;M{\left(\lambda \right)}^{⁎}\cap G_{1}^{⊥}=\{0\}$ *.*

Proof (i) We verify $M(\lambda )x\in G_{1}$ for $x\in G_{0}$. By definition of $G_{0}$ there exists $f_{1}\in \ker\Gamma_{1}$ such that $\Gamma_{0}f_{1}=x$. Together with $\Gamma_{0}\gamma (\lambda )x=x$ we conclude $\gamma (\lambda )x-f_{1}\in \ker\Gamma_{0}$ and

$$M(\lambda )x=\Gamma_{1}\gamma (\lambda )x=\Gamma_{1}\left(\gamma (\lambda )x-f_{1}\right)\in G_{1}.$$

Assume now that $A_{1}$ is self-adjoint and let $\lambda \in \rho (A_{0})\cap \rho (A_{1})$. Since $M(\lambda ):\mathrm{ran}\;\Gamma_{0}\to \mathrm{ran}\;\Gamma_{1}$ is a bijection it suffices to check that $M(\lambda )↾G_{0}$ maps onto $G_{1}$. For $y\in G_{1}$ there exists $f_{0}\in \ker\Gamma_{0}$ with $\Gamma_{1}f_{0}=y$ and $x\in \mathrm{ran}\;\Gamma_{0}$ with M(λ)x=y. Hence we obtain

$$\Gamma_{1}f_{0}=y=M(\lambda )x=\Gamma_{1}\gamma (\lambda )x$$

and therefore $\gamma (\lambda )x-f_{0}\in \ker\Gamma_{1}$ and $\Gamma_{0}(\gamma (\lambda )x-f_{0})=x\in G_{0}$. This completes the proof of item (i).

(ii) We show first that ran *Γ* is contained in the right hand side of (2.10). Let $\overset{ˆ}{x}={\left(x,\;x^{\prime }\right)}^{⊤}\in \mathrm{ran}\;\Gamma$ and choose $f=f_{0}+f_{\lambda }\in \mathrm{dom}\;T=\mathrm{dom}\;A_{0}∔N_{\lambda }(T)$ such that $\Gamma f=\overset{ˆ}{x}$. From (2.6) and $\Gamma_{0}f=x$ we conclude

$$x^{\prime }=\Gamma_{1}f=M(\lambda )\Gamma_{0}f+\Gamma_{1}f_{0}=M(\lambda )x+y,\;\text{where }y:=\Gamma_{1}f_{0}\in G_{1},$$

and hence $\overset{ˆ}{x}$ belongs to the right hand side of (2.10).

Conversely, let $x\in \mathrm{ran}\;\Gamma_{0}$ and $x^{\prime }=M(\lambda )x+y$ with some $y\in G_{1}$. Then there exist $f_{0}\in \ker\Gamma_{0}$ with $\Gamma_{1}f_{0}=y$ and $f_{\lambda }\in N_{\lambda }(T)$ with $\Gamma_{0}f_{\lambda }=x$. Setting $f:=f_{0}+f_{\lambda }\in \mathrm{dom}\;T$ we find $\Gamma_{0}f=x$ and from (2.6) we obtain

$$x^{\prime }=M(\lambda )x+y=M(\lambda )\Gamma_{0}f+\Gamma_{1}f_{0}=\Gamma_{1}f,$$

that is, ${\left(x,x^{\prime }\right)}^{⊤}\in \mathrm{ran}\;\Gamma$ and the identity (2.10) is proved.

The remaining assertion in (ii) follows from the representation (2.10) and the fact that ran *Γ* is dense in G×G.  □

Let again $\left\{G,\Gamma_{0},\Gamma_{1}\right\}$ be a quasi-boundary triple for $T\subset A^{⁎}$ with $A_{0}=T↾\ker\Gamma_{0}$ and Weyl function *M*. For $\lambda \in \rho (A_{0})$ define the operators

(2.11) ReM(λ):=12(M(λ)+M(λ)⁎),dom(ReM(λ))=ranΓ0,ImM(λ):=12i(M(λ)−M(λ)⁎),dom(ImM(λ))=ranΓ0.

Then M(λ)=ReM(λ)+iImM(λ) and it follows from (2.7) that

ImM(λ)=Imλγ(λ)⁎γ(λ),λ∈ρ(A0),

holds. Hence ImM(λ) is a densely defined, invertible bounded operator in G with ran(ImM(λ))⊂G1; cf. (2.4). Therefore we may rewrite Proposition 2.8(ii) in the form

ranΓ={(xx′)∈ranΓ0×ranΓ1:x′=ReM(λ)x+y,y∈G1}.

The continuous extension of ImM(λ) onto G is given by the closure

(2.12) ImM(λ)¯=Imλγ(λ)⁎γ(λ)¯,λ∈ρ(A0).

It is important to note that for λ∈C∖R we have

(2.13) ker⁡(ImM(λ)¯)=ker⁡γ(λ)¯=(ranγ(λ)⁎)⊥=G1⊥,

which may be nontrivial; cf. Proposition 2.17.

### 2.3 Extensions of boundary mappings, *γ*-fields and Weyl functions

Let $\left\{G,\Gamma_{0},\Gamma_{1}\right\}$ be a quasi-boundary triple for $T\subset A^{⁎}$. In this section we investigate the case where the space $G_{1}=\mathrm{ran}(\Gamma_{1}↾\ker\Gamma_{0})$ in Definition 2.7 is dense in G. Under this assumption we show that the boundary map $\Gamma_{0}$ and the *γ*-field admit continuous extensions. If, in addition, $G_{0}=\mathrm{ran}(\Gamma_{0}↾\ker\Gamma_{1})$ is dense in G and $A_{1}=T↾\ker\Gamma_{1}$ is self-adjoint in H then also $\Gamma_{1}$ and the Weyl function *M* admit continuous extensions. We point out that in general $G_{1}$ (or $G_{0}$) is not dense in G, see Proposition 2.17 for a counterexample.

The next proposition is a variant of [28, Proposition 6.3] (see also [26, Lemma 7.22]) for quasi-boundary triples and their Weyl functions. It was proved for generalized boundary triples in [28], where the additional assumption that $G_{1}$ is dense in G is automatically satisfied; cf. (2.13) and [28, Lemma 6.1]. In the following $G_{1}^{\prime }$ stands for the anti-dual space of $G_{1}$.

Proposition 2.9 *Let*$\left\{G,\Gamma_{0},\Gamma_{1}\right\}$*be a quasi-boundary triple for*$T\subset A^{⁎}$*with Weyl function M, set*Λ:=ImM(i)¯*and assume, in addition, that*$G_{1}$*is dense in*G*. Then*

$$G_{1}=\mathrm{ran}\;\Lambda^{1/2}$$

*and if*$G_{1}$*is equipped with the norm induced by the inner product*

(2.14) $${\left(\Lambda^{-1/2}x,\Lambda^{-1/2}y\right)}_{G},\;x,y\in G_{1},$$

*then the following assertions hold.*

(i) γ(i)*extends to an isometry*$\overset{˜}{\gamma }(i)$*from*$G_{1}^{\prime }$*onto*$N_{i}(A^{⁎})$*,*
(ii) ImM(i)*extends to an isometry from*$G_{1}^{\prime }$*onto*$G_{1}$*.*

Proof Since the space $G_{1}$ is dense in G the bounded self-adjoint operator Λ=ImM(i)¯=γ(i)⁎γ(i)¯ is injective and non-negative; cf. (2.12) and (2.13). Hence ran *Λ* and $\mathrm{ran}\;\Lambda^{1/2}$ are dense in G. As in the proof of [28, Proposition 6.3] we equip $G:=\mathrm{ran}\;\Lambda^{1/2}$ with the inner product

$${\left(\Lambda^{-1/2}x,\Lambda^{-1/2}y\right)}_{G},\;x,y\in G.$$

Then G is a Hilbert space which is densely embedded in G and hence gives rise to a Gelfand triple $G↪G↪G^{\prime }$, where $G^{\prime }$ is the completion of G equipped with the inner product ${\left(\Lambda^{1/2}x,\Lambda^{1/2}y\right)}_{G}$, x,y∈G. As in [28, Proposition 6.3] one verifies that the mapping γ(i) admits a continuation to an isometry $\overset{˜}{\gamma }(i)$ from $G^{\prime }$ onto $N_{i}(A^{⁎})$ and the mapping ImM(i) admits a continuation to an isometry $\overset{˜}{\Lambda }$ from $G^{\prime }$ onto G with $\Lambda \subset \overset{˜}{\Lambda }=\gamma {\left(i\right)}^{⁎}\overset{˜}{\gamma }(i)$. This implies $G=\mathrm{ran}\;\gamma {\left(i\right)}^{⁎}=G_{1}$ by (2.9) and assertions (i) and (ii) follow.  □

The next proposition contains a simple but far-reaching observation: If $G_{1}$ is dense in G and $G_{1}$ is equipped with a Hilbert or Banach space norm such that $\left(G_{1},{\left\|\cdot \right\|}_{G_{1}}\right)$ is a reflexive Banach space continuously embedded in G then the boundary map $\Gamma_{0}$ can be extended by continuity onto $\mathrm{dom}\;A^{⁎}$. Although Proposition 2.9 provides a possible norm on $G_{1}$ it is essential for later applications to allow other norms which are a priori not connected with the Weyl function.

Proposition 2.10 *Let*$\left\{G,\Gamma_{0},\Gamma_{1}\right\}$*be a quasi-boundary triple for*$T\subset A^{⁎}$*with*$A_{0}=T↾\ker\Gamma_{0}$*and assume, in addition, that*$G_{1}$*is dense in*G*. Then for any norm*${\left\|\cdot \right\|}_{G_{1}}$*such that*$\left(G_{1},{\left\|\cdot \right\|}_{G_{1}}\right)$*is a reflexive Banach space continuously embedded in*G*, the boundary mapping*$\Gamma_{0}$*admits a unique surjective, continuous extension*

$${\overset{˜}{\Gamma }}_{0}:\left(\mathrm{dom}\;A^{⁎},{\left\|\cdot \right\|}_{A^{⁎}}\right)\to G_{1}^{\prime },$$

*where*$G_{1}^{\prime }$*is the anti-dual space of*$G_{1}$*. Moreover, the norm*$⦀\cdot {⦀}_{G_{1}}$*induced by the inner product*(2.14)*is equivalent to any norm*${\left\|\cdot \right\|}_{G_{1}}$*such that*$\left(G_{1},{\left\|\cdot \right\|}_{G_{1}}\right)$*is a reflexive Banach space continuously embedded in*G*.*

Proof Fix some $\lambda \in \rho (A_{0})$ and define $S:=\Gamma_{1}{\left(A_{0}-\overset{¯}{\lambda }\right)}^{-1}=\gamma {\left(\lambda \right)}^{⁎}$. We show first that $S:H\to G_{1}$ is closed and continuous. In fact, let $h_{n}\to h$ for n→∞ be a sequence in H and assume that $Sh_{n}\to k$, n→∞, in $G_{1}$. Then $Sh_{n}\to k$ in G as the embedding of $G_{1}$ into G is continuous, and

$$Sh_{n}=\gamma (\lambda )h_{n}\to \gamma {\left(\lambda \right)}^{⁎}h=Sh,\;n\to \infty ,$$

in G due to the continuity of $\gamma {\left(\lambda \right)}^{⁎}:H\to G$; cf. Section 2.2. Thus k=Sh and hence *S* is closed as a mapping from H into $G_{1}$. As domS=H we conclude that $S:H\to G_{1}$ is continuous. Moreover, since

$$\ker S=\ker\gamma {\left(\lambda \right)}^{⁎}={\left(\mathrm{ran}\;\gamma (\lambda )\right)}^{⊥}=N_{\lambda }{\left(A^{⁎}\right)}^{⊥}$$

the restriction of *S* onto $N_{\lambda }(A^{⁎})$ is an isomorphism from $N_{\lambda }(A^{⁎})$ onto $G_{1}$. Hence the adjoint operator $S^{\prime }:G_{1}^{\prime }\to H$ is bounded, invertible and by the closed range theorem $\mathrm{ran}\;S^{\prime }=N_{\lambda }(A^{⁎})$. The inverse ${\left(S^{\prime }\right)}^{-1}$ is regarded as an isomorphism from $N_{\lambda }(A^{⁎})$ onto $G_{1}^{\prime }$ in the sequel. For $x\in \mathrm{ran}\;\Gamma_{0}\subset G_{1}^{\prime }$ and h∈H it follows from

$${\left(S^{\prime }x,h\right)}_{H}={\langle x,Sh\rangle }_{G_{1}^{\prime }\times G_{1}}={\left(x,Sh\right)}_{G}={\left(x,\Gamma_{1}{\left(A_{0}-\overset{¯}{\lambda }\right)}^{-1}h\right)}_{G}={\left(\gamma (\lambda )x,h\right)}_{H},$$

that $S^{\prime }↾\mathrm{ran}\;\Gamma_{0}=\gamma (\lambda )$. We define the mapping

$${\overset{˜}{\Gamma }}_{0}:\mathrm{dom}\;A^{⁎}\to G_{1}^{\prime },\;f\mapsto {\overset{˜}{\Gamma }}_{0}f={\left(S^{\prime }\right)}^{-1}f_{\lambda },$$

where $f=f_{0}+f_{\lambda }\in \mathrm{dom}\;A_{0}+N_{\lambda }(A^{⁎})=\mathrm{dom}\;A^{⁎}$. For f∈domT decomposed in the form $f=f_{0}+f_{\lambda }$ with $f_{0}\in \mathrm{dom}\;A_{0}$ and $f_{\lambda }\in N_{\lambda }(T)$ we have

$${\overset{˜}{\Gamma }}_{0}f={\left(S^{\prime }\right)}^{-1}f_{\lambda }={\left(S^{\prime }\right)}^{-1}\gamma (\lambda )\Gamma_{0}f_{\lambda }={\left(S^{\prime }\right)}^{-1}S^{\prime }\Gamma_{0}f_{\lambda }=\Gamma_{0}f_{\lambda }=\Gamma_{0}f,$$

and hence ${\overset{˜}{\Gamma }}_{0}$ is an extension of $\Gamma_{0}$. It remains to check that ${\overset{˜}{\Gamma }}_{0}$ is continuous. For this let $f=f_{0}+f_{\lambda }\in \mathrm{dom}\;A^{⁎}$ and note that $f_{\lambda }=f-{\left(A_{0}-\lambda \right)}^{-1}(A^{⁎}-\lambda )f$ holds. Since ${\left(S^{\prime }\right)}^{-1}:N_{\lambda }(A^{⁎})\to G_{1}^{\prime }$ is bounded we find

$${\left\|{\overset{˜}{\Gamma }}_{0}f\right\|}_{G_{1}^{\prime }}={\left\|{\left(S^{\prime }\right)}^{-1}f_{\lambda }\right\|}_{G_{1}^{\prime }}\leq \left\|{\left(S^{\prime }\right)}^{-1}\right\|\left({\left\|f\right\|}_{H}+{\left\|{\left(A_{0}-\lambda \right)}^{-1}\left(A^{⁎}-\lambda \right)f\right\|}_{H}\right)\leq c{\left\|f\right\|}_{A^{⁎}}$$

with some constant c>0.

Let $⦀\cdot {⦀}_{G_{1}}$ be the norm induced by the inner product (2.14) and let ${\left\|\cdot \right\|}_{G_{1}}$ be an arbitrary norm on $G_{1}$ such that $\left(G_{1},{\left\|\cdot \right\|}_{G_{1}}\right)$ is a reflexive Banach space densely embedded in G. Recall that $\ker\gamma {\left(i\right)}^{⁎}=N_{i}{\left(A^{⁎}\right)}^{⊥}$; cf. (2.5). It follows from Proposition 2.9 that $\gamma {\left(i\right)}^{⁎}$ is an isometry from $N_{i}(A^{⁎})$ onto ${(G_{1},⦀\cdot ⦀}_{G_{1}})$ and hence ${\left(\gamma {\left(i\right)}^{⁎}↾N_{i}(A^{⁎})\right)}^{-1}$ is an isometry from ${(G_{1},⦀\cdot ⦀}_{G_{1}})$ onto $N_{i}(A^{⁎})$. Therefore we obtain

$$⦀x{⦀}_{G_{1}}={\left\|{\left(\gamma {\left(i\right)}^{⁎}↾N_{i}\left(A^{⁎}\right)\right)}^{-1}x\right\|}_{H}\leq c^{\prime }{\left\|x\right\|}_{G_{1}}$$

with $c^{\prime }>0$ for all $x\in G_{1}$. Hence $I:(G_{1},{\left\|\cdot \right\|}_{G_{1}})\to {(G_{1},⦀\cdot ⦀}_{G_{1}})$ is continuous and this implies the norm equivalence $⦀\cdot {⦀}_{G_{1}}∼{\left\|\cdot \right\|}_{G_{1}}$.  □

If $\left\{G,\Gamma_{0},\Gamma_{1}\right\}$ is a quasi-boundary triple for $T\subset A^{⁎}$ with Weyl function *M* and the additional property that $A_{1}=T↾\ker\Gamma_{1}$ is self-adjoint, then the triple $\left\{G,-\Gamma_{1},\Gamma_{0}\right\}$ is also a quasi-boundary triple for $T\subset A^{⁎}$ with Weyl function $\lambda \mapsto -M{\left(\lambda \right)}^{-1}$, $\lambda \in \rho (A_{1})$. This fact together with Proposition 2.10 implies the following statement.

Corollary 2.11 *Let*$\left\{G,\Gamma_{0},\Gamma_{1}\right\}$*be a quasi-boundary triple for*$T\subset A^{⁎}$*and assume, in addition, that*$A_{1}=T↾\ker\Gamma_{1}$*is self-adjoint in*H*and*$G_{0}$*is dense in*G*. Then for any norm*${\left\|\cdot \right\|}_{G_{0}}$*such that*$\left(G_{0},{\left\|\cdot \right\|}_{G_{0}}\right)$*is a reflexive Banach space continuously embedded in*G*the boundary mapping*$\Gamma_{1}$*admits a unique surjective, continuous extension*

$${\overset{˜}{\Gamma }}_{1}:\left(\mathrm{dom}\;A^{⁎},{\left\|\cdot \right\|}_{A^{⁎}}\right)\to G_{0}^{\prime },$$

*where*$G_{0}^{\prime }$*is the anti-dual space of*$G_{0}$*.*

We note that in the situation of the above corollary it follows that the closure of Im(−M(i)−1) is an invertible bounded operator defined on G. Making use of Proposition 2.9 for the quasi-boundary triple $\left\{G,-\Gamma_{1},\Gamma_{0}\right\}$ and setting Σ:=Im(−M(i)−1)¯ we then conclude that the norm $⦀\cdot {⦀}_{G_{0}}$ induced by the inner product

$${\left(\Sigma^{-1/2}x,\Sigma^{-1/2}y\right)}_{G},\;x,y\in G_{0},$$

is equivalent to any norm ${\left\|\cdot \right\|}_{G_{0}}$ on $G_{0}$ such that $\left(G_{0},{\left\|\cdot \right\|}_{G_{0}}\right)$ is a reflexive Banach space continuously embedded in G.

The next theorem is strongly inspired by regularization techniques used in extension theory of symmetric partial differential operators; cf. [39], [72]. It will be shown that a quasi-boundary triple $\left\{G,\Gamma_{0},\Gamma_{1}\right\}$ with the additional property that $G_{1}$ is dense in G can be transformed and extended to an ordinary boundary triple. Such a type of transform appears also in [12], [17] and in a more abstract form in [26], see also [74], [75]. Here we discuss only a situation which is relevant in applications, namely we assume that the spectrum of the self-adjoint operator $A_{0}=T↾\ker\Gamma_{0}$ does not cover the whole real line. The more general case is left to the reader; cf. Remark 2.13. Recall that for the Gelfand triple $G_{1}↪G↪G_{1}^{\prime }$ there exist isometric isomorphisms $\iota_{+}:G_{1}\to G$ and $\iota_{-}:G_{1}^{\prime }\to G$ such that

(2.15) $${\left(\iota_{-}x^{\prime },\;\iota_{+}x\right)}_{G}={\langle x^{\prime },\;x\rangle }_{G_{1}^{\prime }\times G_{1}}\;\text{for all }x\in G_{1},\;x^{\prime }\in G_{1}^{\prime }.$$

Here and in the following $G_{1}$ is equipped with some norm ${\left\|\cdot \right\|}_{G_{1}}$ such that $\left(G_{1},{\left\|\cdot \right\|}_{G_{1}}\right)$ is a reflexive Banach space continuously embedded in G; cf. Proposition 2.10. Recall that according to Proposition 2.9 such a norm always exists (if $G_{1}$ is dense in G) and that all such norms are equivalent by Proposition 2.10.

Theorem 2.12 *Let* $\left\{G,\Gamma_{0},\Gamma_{1}\right\}$ *be a quasi-boundary triple for* $T\subset A^{⁎}$ *with* $A_{0}=A^{⁎}↾\ker\Gamma_{0}$ *, assume that there exists* $\eta \in \rho (A_{0})\cap R$ *and that* $G_{1}$ *is dense in* G *. Then the triple* $\left\{G,{ϒ}_{0},{ϒ}_{1}\right\}$ *with boundary mappings* ${ϒ}_{0},{ϒ}_{1}:\mathrm{dom}\;A^{⁎}\to G$ *given by*

$${ϒ}_{0}f:=\iota_{-}{\overset{˜}{\Gamma }}_{0}f,\;{ϒ}_{1}f:=\iota_{+}\Gamma_{1}f_{0},\;f=f_{0}+f_{\eta }\in \mathrm{dom}\;A_{0}∔N_{\eta }\left(A^{⁎}\right),$$

*is an ordinary boundary triple for* $A^{⁎}$ *with*

$$A^{⁎}↾\ker{ϒ}_{0}=A_{0}\;\text{and}\;A^{⁎}↾\ker{ϒ}_{1}=A∔{\overset{ˆ}{N}}_{\eta }\left(A^{⁎}\right).$$

Proof We verify that the restriction $\left\{G,{ϒ}_{0}^{T},{ϒ}_{1}^{T}\right\}$,

$${ϒ}_{0}^{T}f=\iota_{-}\Gamma_{0}f,\;{ϒ}_{1}^{T}f=\iota_{+}\Gamma_{1}f_{0},\;f=f_{0}+f_{\eta }\in \mathrm{dom}\;A_{0}∔N_{\eta }(T),$$

of the triple $\left\{G,{ϒ}_{0},{ϒ}_{1}\right\}$ on *T* is a quasi-boundary triple for $T\subset A^{⁎}$, such that the boundary mapping ${ϒ}^{T}={\left({ϒ}_{0}^{T},{ϒ}_{1}^{T}\right)}^{⊤}:\mathrm{dom}\;T\to G\times G$ is continuous with respect to the graph norm of $A^{⁎}$. Then Proposition 2.4 implies that $\left\{G,{ϒ}_{0},{ϒ}_{1}\right\}$ is an ordinary boundary triple for $A^{⁎}$.

Note first that $\ker{ϒ}_{0}^{T}=\ker\Gamma_{0}$ holds. Thus $T↾\ker{ϒ}_{0}^{T}$ coincides with the self-adjoint linear operator $A_{0}$ in H and (iii) in Definition 2.1 holds. In order to check Green's identity observe that for all f∈domT the identity ${ϒ}_{1}^{T}f=\iota_{+}(\Gamma_{1}f-M(\eta )\Gamma_{0}f)$ holds by (2.6). Here *M* is the Weyl function of the quasi-boundary triple $\left\{G,\Gamma_{0},\Gamma_{1}\right\}$ and since by assumption $\eta \in R\cap \rho (A_{0})$ the operator M(η) is symmetric in G; cf. (2.7). Making use of (2.15) and the fact that ${\langle \cdot ,\cdot \rangle }_{G_{1}\times G_{1}^{\prime }}$ is the continuous extension of the scalar product in G we compute for all f,g∈domT

$${\left({ϒ}_{1}^{T}f,{ϒ}_{0}^{T}g\right)}_{G}-{\left({ϒ}_{0}^{T}f,{ϒ}_{1}^{T}g\right)}_{G}={\langle \Gamma_{1}f-M(\eta )\Gamma_{0}f,\Gamma_{0}g\rangle }_{G_{1}\times G_{1}^{\prime }}-{\langle \Gamma_{0}f,\Gamma_{1}g-M(\eta )\Gamma_{0}g\rangle }_{G_{1}^{\prime }\times G_{1}}={\left(\Gamma_{1}f-M(\eta )\Gamma_{0}f,\Gamma_{0}g\right)}_{G}-{\left(\Gamma_{0}f,\Gamma_{1}g-M(\eta )\Gamma_{0}g\right)}_{G}={\left(\Gamma_{1}f,\Gamma_{0}g\right)}_{G}-{\left(\Gamma_{0}f,\Gamma_{1}g\right)}_{G}={\left(Tf,g\right)}_{H}-{\left(f,Tg\right)}_{H}.$$

Now we verify that $\mathrm{ran}\;{ϒ}^{T}$ is dense in G×G. For this let $\overset{ˆ}{x}={\left(x,x^{\prime }\right)}^{⊤}\in G\times G$. Then there exists $\xi^{\prime }\in G_{1}$ such that $\iota_{+}\xi^{\prime }=x^{\prime }$ and $f_{0}\in \ker\Gamma_{0}=\mathrm{dom}\;A_{0}$ such that $\Gamma_{1}f_{0}=\xi^{\prime }$. Note that $\mathrm{ran}\;{ϒ}_{0}^{T}$ is dense in G since $\mathrm{ran}\;\Gamma_{0}$ is dense in G. Hence we find a sequence $\left(f_{n})\subset N_{\eta }(T\right)$ such that ${ϒ}_{0}^{T}f_{n}\to x$, n→∞. It follows from ${ϒ}_{0}^{T}f_{0}=0$ and the definition of ${ϒ}_{1}^{T}$ that

$${ϒ}^{T}(f_{0}+f_{n})=\left(\begin{array}{c} {ϒ}_{0}^{T}(f_{0}+f_{n})\\ {ϒ}_{1}^{T}(f_{0}+f_{n}) \end{array}\right)=\left(\begin{array}{c} {ϒ}_{0}^{T}f_{n}\\ \iota_{+}\Gamma_{1}f_{0} \end{array}\right)=\left(\begin{array}{c} {ϒ}_{0}^{T}f_{n}\\ x^{\prime } \end{array}\right)$$

tends to $\overset{ˆ}{x}$ for n→∞. Hence (ii) in Definition 2.1 holds and it follows that $\left\{G,{ϒ}_{0}^{T},{ϒ}_{1}^{T}\right\}$ is a quasi-boundary triple.

Now we have to check that ${ϒ}_{0}^{T},{ϒ}_{1}^{T}:\mathrm{dom}\;T\to G$ are continuous with respect to the graph norm. It follows from Proposition 2.10 that this is even true for ${ϒ}_{0}=\iota_{-}{\overset{˜}{\Gamma }}_{0}$, and hence also for the restriction ${ϒ}_{0}^{T}$. For $f=f_{0}+f_{\eta }\in \mathrm{dom}\;T$ with $f_{0}\in \mathrm{dom}\;A_{0}$ and $f_{\eta }\in N_{\eta }(T)$ we have

$${ϒ}_{1}^{T}f=\iota_{+}\Gamma_{1}f_{0}=\iota_{+}\Gamma_{1}{\left(A_{0}-\eta \right)}^{-1}(T-\eta )f.$$

Since $\Gamma_{1}{\left(A_{0}-\eta \right)}^{-1}:H\to G_{1}$ is continuous (see the proof of Proposition 2.10) we conclude that ${ϒ}_{1}^{T}$ is continuous with respect to the graph norm.

It remains to check that $\ker{ϒ}_{1}=\mathrm{dom}\;A∔N_{\eta }(A^{⁎})$. For the inclusion “⊂” let $f\in \ker{ϒ}_{1}$ with $f=f_{0}+f_{\eta }\in \mathrm{dom}\;A_{0}∔N_{\eta }(A^{⁎})$. Since $\Gamma_{1}f_{0}=0$ we find $f_{0}\in \mathrm{dom}\;A_{0}\cap \ker\Gamma_{1}=\mathrm{dom}\;A$ and hence $f\in \mathrm{dom}\;A∔N_{\eta }(A^{⁎})$. The inclusion “⊃” follows immediately from $\mathrm{dom}\;A\subset \ker\Gamma_{1}$ and $\Gamma_{1}f_{\eta }=0$ for $f_{\eta }\in N_{\eta }(A^{⁎})$.  □

Remark 2.13 We note that the assumption η∈R in Theorem 2.12 can be dropped. In fact, if η∈C∖R replace M(η) and $N_{\eta }(A^{⁎})$ by ReM(η) (see (2.11)) and

$$Q_{\eta }\left(A^{⁎}\right):=\left\{f_{\eta }+f_{\overset{¯}{\eta }}:f\in \mathrm{dom}\;A^{⁎}\right\},$$

respectively. Here $f=f_{0\eta }+f_{\eta }=f_{0\overset{¯}{\eta }}+f_{\overset{¯}{\eta }}\in \mathrm{dom}\;A^{⁎}$ with $f_{0\eta },f_{0\overset{¯}{\eta }}\in \mathrm{dom}\;A_{0}$ and $f_{\eta }\in N_{\eta }(A^{⁎})$, $f_{\overset{¯}{\eta }}\in N_{\overset{¯}{\eta }}(A^{⁎})$. Instead of (2.6) use the following formula

Γ1f0=Γ1f−ReM(η)Γ0f,f=f0+12(fη+fη¯)∈domA0∔Qη(A⁎),

when verifying Green's identity in the proof of Theorem 2.12.

With the help of the extensions ${\overset{˜}{\Gamma }}_{0}$ and ${\overset{˜}{\Gamma }}_{1}$ of the boundary mappings $\Gamma_{0}$ and $\Gamma_{1}$, respectively, also the *γ*-field and Weyl function can be extended by continuity. Observe that by Theorem 2.12 we have $\ker{\overset{˜}{\Gamma }}_{0}=\ker{ϒ}_{0}=\mathrm{dom}\;A_{0}$ and hence ${\overset{˜}{\Gamma }}_{0}↾N_{\lambda }(A^{⁎})$, $\lambda \in \rho (A_{0})$, is invertible.

Definition 2.14 Let $\left\{G,\Gamma_{0},\Gamma_{1}\right\}$ be a quasi-boundary triple for $T\subset A^{⁎}$ with *γ*-field *γ*, Weyl function *M* and $A_{j}=T↾\ker\Gamma_{j}$, j=0,1.

(i) Assume that $G_{1}$ is dense in G and let ${\overset{˜}{\Gamma }}_{0}:\mathrm{dom}\;A^{⁎}\to G_{1}^{\prime }$ be the continuous extension of $\Gamma_{0}$ from Proposition 2.10. Then the extended *γ*-field $\overset{˜}{\gamma }$ corresponding to the quasi-boundary triple $\left\{G,\Gamma_{0},\Gamma_{1}\right\}$ is defined by
  $$\lambda \mapsto \overset{˜}{\gamma }(\lambda ):={\left({\overset{˜}{\Gamma }}_{0}↾N_{\lambda }\left(A^{⁎}\right)\right)}^{-1}:G_{1}^{\prime }\to H,\;\lambda \in \rho (A_{0}).$$
(ii) Assume that $G_{0}$ and $G_{1}$ are dense in G, that $A_{1}$ is self-adjoint in H, and let ${\overset{˜}{\Gamma }}_{1}:\mathrm{dom}\;A^{⁎}\to G_{0}^{\prime }$ be the continuous extension of $\Gamma_{1}$ from Corollary 2.11. Then the extended Weyl function $\overset{˜}{M}$ corresponding to the quasi-boundary triple $\left\{G,\Gamma_{0},\Gamma_{1}\right\}$ is defined by
  $$\lambda \mapsto \overset{˜}{M}(\lambda ):={\overset{˜}{\Gamma }}_{1}\overset{˜}{\gamma }(\lambda ):G_{1}^{\prime }\to G_{0}^{\prime },\;\lambda \in \rho (A_{0}).$$

We mention that the values of the extended *γ*-field $\overset{˜}{\gamma }$ are bounded linear operators from $G_{1}^{\prime }$ to H, where $G_{1}$ is equipped with a norm ${\left\|\cdot \right\|}_{G_{1}}$ such that $\left(G_{1},{\left\|\cdot \right\|}_{G_{1}}\right)$ is a reflexive Banach space continuously embedded in G. If also $G_{0}$ is equipped with a norm ${\left\|\cdot \right\|}_{G_{0}}$ such that $\left(G_{0},{\left\|\cdot \right\|}_{G_{0}}\right)$ is a reflexive Banach space continuously embedded in G then the values of the extended Weyl function $\overset{˜}{M}$ are bounded linear operators from $G_{1}^{\prime }$ to $G_{0}^{\prime }$. Therefore the adjoints

$$\overset{˜}{\gamma }{\left(\lambda \right)}^{\prime }:H\to G_{1}\;\text{and}\;\overset{˜}{M}{\left(\lambda \right)}^{\prime }:G_{0}\to G_{1}$$

are continuous for all $\lambda \in \rho (A_{0})$. Moreover we obtain the simple identity

(2.16) $$\overset{˜}{M}(\lambda ){\overset{˜}{\Gamma }}_{0}f_{\lambda }={\overset{˜}{\Gamma }}_{1}f_{\lambda }\;\text{for all }f_{\lambda }\in N_{\lambda }\left(A^{⁎}\right),\;\lambda \in \rho (A_{0}).$$

In the next two lemmas some basic, but important, facts about the extended boundary mappings, the extended *γ*-field and the extended Weyl function are summarized. As above it is assumed that $G_{1}$ is dense in G and that $G_{1}$ is equipped with a norm such that $\left(G_{1},{\left\|\cdot \right\|}_{G_{1}}\right)$ is a reflexive Banach space continuously embedded in G.

Lemma 2.15 *Let*$\left\{G,\Gamma_{0},\Gamma_{1}\right\}$*be a quasi-boundary triple for*$T\subset A^{⁎}$*with γ-field γ, and*$A_{0}=T↾\ker\Gamma_{0}$*such that*$\rho (A_{0})\cap R\neq \emptyset$*. Assume that*$G_{1}$*is dense in*G*. Then the following statements hold.*

(i) $\ker{\overset{˜}{\Gamma }}_{0}=\ker\Gamma_{0}=\mathrm{dom}\;A_{0}$*,*
(ii) $\overset{˜}{\gamma }(\lambda )$*is an isomorphism from*$G_{1}^{\prime }$*onto*$N_{\lambda }(A^{⁎})\subset H$*for all*$\lambda \in \rho (A_{0})$*,*
(iii) $\overset{˜}{\gamma }{\left(\lambda \right)}^{\prime }=\Gamma_{1}{\left(A_{0}-\overset{¯}{\lambda }\right)}^{-1}:H\to G_{1}$*is continuous and surjective for all*$\lambda \in \rho (A_{0})$*,*
(iv) *the identity*
  $$\overset{˜}{\gamma }(\lambda )=\left(I+(\lambda -\mu ){\left(A_{0}-\lambda \right)}^{-1}\right)\overset{˜}{\gamma }(\mu )$$
  *holds for all*$\lambda ,\mu \in \rho (A_{0})$*.*

Proof Let $\left\{G,{ϒ}_{0},{ϒ}_{1}\right\}$ be the ordinary boundary triple for $A^{⁎}$ from Theorem 2.12 and denote the corresponding *γ*-field with *β*. Then according to Theorem 2.12 statement (i) follows from

$$\ker\Gamma_{0}=\mathrm{dom}\;A_{0}=\ker{ϒ}_{0}=\ker\iota_{-}{\overset{˜}{\Gamma }}_{0}=\ker{\overset{˜}{\Gamma }}_{0},$$

see the text before Definition 2.14. From Proposition 2.10 we obtain that ${\overset{˜}{\Gamma }}_{0}:(\mathrm{dom}\;A^{⁎},\;{\left\|\cdot \right\|}_{A^{⁎}})\to G_{1}^{\prime }$ is continuous and surjective with $\ker{\overset{˜}{\Gamma }}_{0}=\mathrm{dom}\;A_{0}$; cf. (i). Hence ${\overset{˜}{\Gamma }}_{0}:N_{\lambda }(A^{⁎})\to G_{1}^{\prime }$ is bijective and continuous and this implies (ii). The identity

$$\beta (\lambda )=\left(I+(\lambda -\mu ){\left(A_{0}-\lambda \right)}^{-1}\right)\beta (\mu ),\;\lambda ,\mu \in \rho (A_{0}),$$

(see (2.4)) together with the straightforward computation

$$\beta (\lambda )={\left({ϒ}_{0}↾N_{\lambda }\left(A^{⁎}\right)\right)}^{-1}={\left(\iota_{-}{\overset{˜}{\Gamma }}_{0}↾N_{\lambda }\left(A^{⁎}\right)\right)}^{-1}=\overset{˜}{\gamma }(\lambda )\iota_{-}^{-1}$$

implies (iv). To prove statement (iii) we only have to show that the identity $\overset{˜}{\gamma }{\left(\lambda \right)}^{\prime }=\Gamma_{1}{\left(A_{0}-\overset{¯}{\lambda }\right)}^{-1}$ holds. With f∈H and x∈G it follows from

$${\left(\beta {\left(\lambda \right)}^{⁎}f,\;x\right)}_{G}={\left(f,\beta (\lambda )x\right)}_{H}={\left(f,\overset{˜}{\gamma }(\lambda )\iota_{-}^{-1}x\right)}_{H}={\langle \overset{˜}{\gamma }{\left(\lambda \right)}^{\prime }f,\iota_{-}^{-1}x\rangle }_{G_{1}\times G_{1}^{\prime }}={\left(\iota_{+}\overset{˜}{\gamma }{\left(\lambda \right)}^{\prime }f,\iota_{-}\iota_{-}^{-1}x\right)}_{G}={\left(\iota_{+}\overset{˜}{\gamma }{\left(\lambda \right)}^{\prime }f,x\right)}_{G}$$

that $\iota_{+}\overset{˜}{\gamma }{\left(\lambda \right)}^{\prime }=\beta {\left(\lambda \right)}^{⁎}={ϒ}_{1}{\left(A_{0}-\overset{¯}{\lambda }\right)}^{-1}=\iota_{+}\Gamma_{1}{\left(A_{0}-\overset{¯}{\lambda }\right)}^{-1}$. Hence we obtain statement (iii).  □

Lemma 2.16 *Let the assumption be as in* Lemma 2.15 *and assume, in addition, that* $G_{0}$ *is dense in* G *and that* $A_{1}=T↾\ker\Gamma_{1}$ *is self-adjoint in* H *such that* $\rho (A_{1})\cap R\neq \emptyset$ *. Moreover, equip* $G_{0}$ *with a norm* ${\left\|\cdot \right\|}_{G_{0}}$ *such that* $\left(G_{0},{\left\|\cdot \right\|}_{G_{0}}\right)$ *is a reflexive Banach space continuously embedded in* G *. Then the following statements hold for all* $\lambda \in \rho (A_{0})$ *.*

(i) $\ker{\overset{˜}{\Gamma }}_{1}=\ker\Gamma_{1}=\mathrm{dom}\;A_{1}$ *,*
(ii) ${\overset{˜}{\Gamma }}_{1}f=\overset{˜}{M}(\lambda ){\overset{˜}{\Gamma }}_{0}f+\Gamma_{1}f_{0}$ *for all* $f=f_{0}+f_{\lambda }\in \mathrm{dom}\;A_{0}∔N_{\lambda }(A^{⁎})$ *,*
(iii) $\overset{˜}{M}{\left(\lambda \right)}^{\prime }x=M{\left(\lambda \right)}^{⁎}x=M(\overset{¯}{\lambda })x$ *for all* $x\in G_{0}$ *,*
(iv) *if, in addition,* $\lambda \in \rho (A_{1})$ *then* $\overset{˜}{M}(\lambda ):G_{1}^{\prime }\to G_{0}^{\prime }$ *and* $M(\lambda )↾G_{0}:G_{0}\to G_{1}$ *are isomorphisms,*
(v) *the range of the boundary mapping* $\overset{˜}{\Gamma }$ *is given by*
  $$\mathrm{ran}\;\overset{˜}{\Gamma }=\left\{\left(\begin{array}{c} x\\ x^{\prime } \end{array}\right)\in G_{1}^{\prime }\times G_{0}^{\prime }:x^{\prime }=\overset{˜}{M}(\lambda )x+y,\;y\in G_{1}\right\}.$$

Proof Statement (i) follows in the same way as in Lemma 2.15 and from the fact that $\left\{G,-\Gamma_{1},\Gamma_{0}\right\}$ is a quasi-boundary triple for $T\subset A^{⁎}$.

The identity (2.16) together with $f=f_{0}+f_{\lambda }\in \mathrm{dom}\;A_{0}∔N_{\lambda }(A^{⁎})$ yields the identity

$${\overset{˜}{\Gamma }}_{1}f={\overset{˜}{\Gamma }}_{1}f_{0}+{\overset{˜}{\Gamma }}_{1}f_{\lambda }=\Gamma_{1}f_{0}+\overset{˜}{M}(\lambda ){\overset{˜}{\Gamma }}_{0}f_{\lambda }=\Gamma_{1}f_{0}+\overset{˜}{M}(\lambda ){\overset{˜}{\Gamma }}_{0}f,$$

therefore (ii) holds; cf. (2.6). In order to verify (iii) note first that according to (2.8) we have $G_{0}\subset \mathrm{ran}\;\Gamma_{0}=\mathrm{dom}\;M(\lambda )=\mathrm{dom}\;M(\overset{¯}{\lambda })\subset \mathrm{dom}\;M{\left(\lambda \right)}^{⁎}$. For $x\in G_{0}$ and $y\in \mathrm{ran}\;\Gamma_{0}\subset G\subset G_{j}^{\prime }$, j=0,1, we compute

$${\left(M{\left(\lambda \right)}^{⁎}x,y\right)}_{G}={\left(x,\;M(\lambda )y\right)}_{G}={\langle x,\;\overset{˜}{M}(\lambda )y\rangle }_{G_{0}\times G_{0}^{\prime }}={\langle \overset{˜}{M}{\left(\lambda \right)}^{\prime }x,y\rangle }_{G_{1}\times G_{1}^{\prime }}={\left(\overset{˜}{M}{\left(\lambda \right)}^{\prime }x,y\right)}_{G}.$$

As $\mathrm{ran}\;\Gamma_{0}$ is dense in G this implies $M{\left(\lambda \right)}^{⁎}x=\overset{˜}{M}{\left(\lambda \right)}^{\prime }x$ and $M(\overset{¯}{\lambda })x=M{\left(\lambda \right)}^{⁎}x$ holds by (2.7), (2.8).

By Lemma 2.15(ii) the operator $\overset{˜}{\gamma }(\lambda )$ is an isomorphism from $G_{1}^{\prime }$ onto $N_{\lambda }(A^{⁎})$. Since $A_{1}$ is self-adjoint in H we have $\mathrm{dom}\;A^{⁎}=\mathrm{dom}\;A_{1}∔N_{\lambda }(A^{⁎})$ for $\lambda \in \rho (A_{1})$. Therefore the first assertion in (iv) follows from (i) and Corollary 2.11. The second assertion in (iv) is a consequence of (iii). Finally, statement (v) follows from (ii) in the same way as in the proof of Proposition 2.8(ii).  □

Since $\ker\Gamma_{1}=\ker{\overset{˜}{\Gamma }}_{1}$ and $\ker\Gamma_{0}=\ker{\overset{˜}{\Gamma }}_{0}$ hold by Lemma 2.16(i) and Lemma 2.15(i) we conclude that the spaces $G_{0}$ and $G_{1}$ in Definition 2.7 remain the same for the extended boundary mappings, i.e.,

$$G_{0}=\mathrm{ran}(\Gamma_{0}↾\ker\Gamma_{1})=\mathrm{ran}({\overset{˜}{\Gamma }}_{0}↾\ker{\overset{˜}{\Gamma }}_{1}),$$

$$G_{1}=\mathrm{ran}(\Gamma_{1}↾\ker\Gamma_{0})=\mathrm{ran}({\overset{˜}{\Gamma }}_{1}↾\ker{\overset{˜}{\Gamma }}_{0}).$$

For later purposes we also note that for a quasi-boundary triple $\left\{G,\Gamma_{0},\Gamma_{1}\right\}$ as in Lemma 2.15, Lemma 2.16, with *γ*-field *γ*, Weyl function *M*, their extensions $\overset{˜}{\gamma }(\lambda ):G_{1}^{\prime }\to H$ and $\overset{˜}{M}(\lambda ):G_{1}^{\prime }\to G_{0}^{\prime }$, and the corresponding ordinary boundary triple $\left\{G,{ϒ}_{0},{ϒ}_{1}\right\}$ from Theorem 2.12 with *γ*-field *β*, Weyl function M the following relations hold:

(2.17) $$\beta (\lambda )=\overset{˜}{\gamma }(\lambda )\iota_{-}^{-1}\;\text{and}\;M(\lambda )=\iota_{+}\left(\overset{˜}{M}(\lambda )-\overset{˜}{M}(\eta )\right)\iota_{-}^{-1},\;\lambda \in \rho (A_{0}),$$

where $\eta \in \rho (A_{0})\cap R$ is as in Theorem 2.12. In fact, the identity $\beta (\lambda )=\overset{˜}{\gamma }(\lambda )\iota_{-}^{-1}$ was already shown in the proof of Lemma 2.15 and the second relation in (2.17) is a direct consequence of the definition of the Weyl function M, Lemma 2.16(ii), and the particular form of the ordinary boundary triple $\left\{G,{ϒ}_{0},{ϒ}_{1}\right\}$ in Theorem 2.12. More precisely, for $f_{\lambda }\in N_{\lambda }(A^{⁎})$ decomposed in the form $f_{\lambda }=f_{0}+f_{\eta }$ with $f_{0}\in \mathrm{dom}\;A_{0}$, $f_{\eta }\in N_{\eta }(A^{⁎})$, one has

$$\iota_{+}\left(\overset{˜}{M}(\lambda )-\overset{˜}{M}(\eta )\right)\iota_{-}^{-1}{ϒ}_{0}f_{\lambda }=\iota_{+}\left(\overset{˜}{M}(\lambda )-\overset{˜}{M}(\eta )\right){\overset{˜}{\Gamma }}_{0}f_{\lambda }=\iota_{+}\left({\overset{˜}{\Gamma }}_{1}f_{\lambda }-\overset{˜}{M}(\eta ){\overset{˜}{\Gamma }}_{0}f_{\lambda }\right)=\iota_{+}\Gamma_{1}f_{0}={ϒ}_{1}f_{\lambda }.$$

### 2.4 A counterexample

In this supplementary subsection we show that the assumption $G_{1}^{⊥}=\{0\}$, which is essential for Proposition 2.9, Proposition 2.10, Corollary 2.11 and Theorem 2.12, is not satisfied automatically. For this we construct a quasi-boundary triple $\left\{H,{ϒ}_{0},{ϒ}_{1}\right\}$ with the property $G_{1}^{⊥}\neq \{0\}$ as a transform of the quasi-boundary triple in Lemma 2.5(ii).

Proposition 2.17 *Let*$\left\{N_{\eta }(A^{⁎}),\Gamma_{0}^{T},\Gamma_{1}^{T}\right\}$*be the quasi-boundary triple for*$T\subset A^{⁎}$*from*Lemma 2.5(ii) *with*φ=I*,*$G=N_{\eta }(A^{⁎})$*, and let*H*be an auxiliary Hilbert space. Choose a densely defined, bounded operator*$\gamma :H\to N_{\eta }(A^{⁎})$*such that*

$$\ker\gamma =\{0\},\;\mathrm{ran}\;\gamma =N_{\eta }(T)\;\text{and}\;\ker\bar{\gamma }\neq \{0\},$$

*and let M be an (unbounded) self-adjoint operator in*H*defined on* dom *γ. Then* $\left\{H,{ϒ}_{0},{ϒ}_{1}\right\}$*, where*

$${ϒ}_{0}f:=\gamma^{-1}\Gamma_{0}^{T}f,\;{ϒ}_{1}f:=\gamma^{⁎}\Gamma_{1}^{T}f+M\gamma^{-1}\Gamma_{0}^{T}f,\;f\in \mathrm{dom}\;T,$$

*is a quasi-boundary triple for* $T\subset A^{⁎}$ *such that* $A_{0}=T↾\ker{ϒ}_{0}$*,*

$$G_{1}=\mathrm{ran}({ϒ}_{1}↾\ker{ϒ}_{0})=\mathrm{ran}\;\gamma^{⁎}\;\text{and}\;G_{1}^{⊥}=\ker\bar{\gamma }\neq \{0\}.$$

*In particular, if* M(⋅) *is the Weyl function corresponding to the quasi-boundary triple* $\left\{H,{ϒ}_{0},{ϒ}_{1}\right\}$ *then we have* M(η)=M *and* ImM(λ)¯ *is not invertible for any* λ∈C∖R*.*

Proof We verify that $\left\{H,{ϒ}_{0},{ϒ}_{1}\right\}$ is a quasi-boundary triple for $T\subset A^{⁎}$. Since *M* is self-adjoint in H and $\left\{N_{\eta }(A^{⁎}),\Gamma_{0}^{T},\Gamma_{1}^{T}\right\}$ is a quasi-boundary triple we have

$${\left({ϒ}_{1}f,{ϒ}_{0}g\right)}_{H}-{\left({ϒ}_{0}f,{ϒ}_{1}g\right)}_{H}={\left(\gamma^{⁎}\Gamma_{1}^{T}f,\gamma^{-1}\Gamma_{0}^{T}g\right)}_{H}-{\left(\gamma^{-1}\Gamma_{0}^{T}f,\gamma^{⁎}\Gamma_{1}^{T}g\right)}_{H}={\left(\Gamma_{1}^{T}f,\gamma \gamma^{-1}\Gamma_{0}^{T}g\right)}_{N_{\eta }(A^{⁎})}-{\left(\gamma \gamma^{-1}\Gamma_{0}^{T}f,\Gamma_{1}^{T}g\right)}_{N_{\eta }(A^{⁎})}={\left(\Gamma_{1}^{T}f,\Gamma_{0}^{T}g\right)}_{N_{\eta }(A^{⁎})}-{\left(\Gamma_{0}^{T}f,\Gamma_{1}^{T}g\right)}_{N_{\eta }(A^{⁎})}={\left(Tf,g\right)}_{H}-{\left(f,Tg\right)}_{H}$$

for all f,g∈domT, and hence the abstract Green's identity holds. Observe that

$$A_{0}=T↾\ker\Gamma_{0}^{T}=T↾\ker{ϒ}_{0}$$

holds since by assumption *γ* is a bijection from dom *γ* onto $N_{\eta }(T)$.

Next it will be shown that the range of $ϒ:={\left({ϒ}_{0},{ϒ}_{1}\right)}^{⊤}$ is dense in H×H. Since $\gamma^{-1}$ is a bijection from $N_{\eta }(T)$ onto dom *γ* we have

$$\mathrm{ran}\;ϒ=\left\{\left(\begin{array}{c} \gamma^{-1}\Gamma_{0}^{T}f\\ \gamma^{⁎}\Gamma_{1}^{T}f+M\gamma^{-1}\Gamma_{0}^{T}f \end{array}\right):f\in \mathrm{dom}\;T\right\}=\left\{\left(\begin{array}{c} \gamma^{-1}f_{\eta }\\ \gamma^{⁎}\Gamma_{1}^{T}f_{0}+M\gamma^{-1}f_{\eta } \end{array}\right):f=f_{0}+f_{\eta }\in \ker{ϒ}_{0}∔N_{\eta }(T)\right\}=\left\{\left(\begin{array}{c} x\\ y+Mx \end{array}\right):x\in \mathrm{dom}\;\gamma ,\;y\in \mathrm{ran}\;\gamma^{⁎}\right\}.$$

Here we have used in the last step that $\mathrm{ran}\;\Gamma_{1}^{T}=N_{\eta }(A^{⁎})$ by Lemma 2.5(ii). Suppose that $(z,z^{\prime })\in {\left(\mathrm{ran}\;ϒ\right)}^{⊥}$. Then

(2.18) $${\left(z,x\right)}_{H}+{\left(z^{\prime },y\right)}_{H}+{\left(z^{\prime },Mx\right)}_{H}=0$$

for all x∈domγ and all $y\in \mathrm{ran}\;\gamma^{⁎}$. We note that if $z^{\prime }=0$ then z=0 as dom *γ* is dense in H. Assume first that $z^{\prime }\in \ker\bar{\gamma }={\left(\mathrm{ran}\;\gamma^{⁎}\right)}^{⊥}$. Then ${\left(z^{\prime },y\right)}_{H}=0$, $y\in \mathrm{ran}\;\gamma^{⁎}$, and (2.18) yields

$${\left(z^{\prime },Mx\right)}_{H}={\left(-z,x\right)}_{H}$$

for all x∈domM. As *M* is self-adjoint we conclude $z^{\prime }\in \mathrm{dom}\;M=\mathrm{dom}\;\gamma$ and from ker⁡γ={0} we find $z^{\prime }=0$. Assume now that $z^{\prime }\notin \ker\bar{\gamma }={\left(\mathrm{ran}\;\gamma^{⁎}\right)}^{⊥}$. Then there exists $y\in \mathrm{ran}\;\gamma^{⁎}$ such that ${\left(z^{\prime },y\right)}_{H}\neq 0$ which is a contradiction to (2.18) when setting x=0. Thus we conclude $z^{\prime }=z=0$ and hence ran *ϒ* is dense in H×H.

Since $\ker{ϒ}_{0}=\ker\Gamma_{0}^{T}$ and $\mathrm{ran}(\Gamma_{1}^{T}↾\ker\Gamma_{0}^{T})=N_{\eta }(A^{⁎})$ we have

$$G_{1}=\mathrm{ran}({ϒ}_{1}↾\ker{ϒ}_{0})=\mathrm{ran}\;\left(\gamma^{⁎}\Gamma_{1}^{T}↾\ker\Gamma_{0}^{T}\right)=\mathrm{ran}\;\gamma^{⁎}$$

and therefore $G_{1}^{⊥}=\ker\bar{\gamma }\neq \{0\}$ by assumption. Finally, if M(⋅) is the Weyl function corresponding to the quasi-boundary triple $\left\{H,{ϒ}_{0},{ϒ}_{1}\right\}$ then it follows from $\Gamma_{1}^{T}f_{\eta }=0$, $f_{\eta }\in N_{\eta }(T)$, and $M{ϒ}_{0}f_{\eta }=M\gamma^{-1}\Gamma_{0}^{T}f_{\eta }={ϒ}_{1}f_{\eta }$ that M(η)=M holds. The fact that $\bar{\text{Im}\;M(\lambda )}$ is not invertible for λ∈C∖R is immediate from (2.13).  □

## 3 Extensions of symmetric operators

The main objective of this section is to parameterize the extensions of a symmetric operator *A* with the help of a quasi-boundary triple $\left\{G,\Gamma_{0},\Gamma_{1}\right\}$ for $T\subset A^{⁎}$. In contrast to ordinary boundary triples there is no immediate direct connection between the properties of the extensions

(3.1) $$A_{\vartheta }=T↾\{f\in \mathrm{dom}\;T:\Gamma f\in \vartheta \}$$

and the properties of the corresponding parameters *ϑ* in G×G, as, e.g. self-adjointness. The key idea in Theorem 3.3 and Theorem 3.4 is to mimic a regularization procedure which is used in the investigation of elliptic differential operators and goes back to [39], [72], see also [12], [17], [26], [36], [53], [60], [62]. This also leads to an abstract complete description of the extensions $A_{\vartheta }\subset A^{⁎}$ via the extended boundary mappings ${\overset{˜}{\Gamma }}_{0}$ and ${\overset{˜}{\Gamma }}_{1}$ in Theorem 3.7. The general results are illustrated with various examples and sufficient conditions on the parameters to imply self-adjointness, as well as a variant of Kreĭn's formula is discussed.

### 3.1 Parameterization of extensions with quasi-boundary triples

Let in the following *A* be a closed, densely defined, symmetric operator in the Hilbert space H with equal, in general, infinite deficiency indices. In the first theorem in this subsection we recall one of the key features of ordinary boundary triples $\left\{G,\Gamma_{0},\Gamma_{1}\right\}$ for $A^{⁎}$: A complete description and parameterization of the extensions $A_{\Theta }$ of *A* given by

$$A_{\Theta }:=A^{⁎}↾\left\{f\in \mathrm{dom}\;A^{⁎}:\Gamma f\in \Theta \right\}$$

and their properties in terms of linear relations *Θ* in the boundary space G, see, e.g. [27], [28], [38].

Theorem 3.1 *Let*$\left\{G,\Gamma_{0},\Gamma_{1}\right\}$*be an ordinary boundary triple for*$A^{⁎}$*. Then the mapping*^2^

$$\Theta \mapsto A_{\Theta }=A^{⁎}↾\left\{f\in \mathrm{dom}\;A^{⁎}:\Gamma f\in \Theta \right\}=A^{⁎}↾\ker(\Gamma_{1}-\Theta \Gamma_{0})$$

*establishes a bijective correspondence between the set of closed linear relations Θ in*G*and the set of closed extensions*$A_{\Theta }\subset A^{⁎}$*of A. Furthermore,*

$$A_{\Theta^{⁎}}=A_{\Theta }^{⁎}$$

*and the operator*$A_{\Theta }$*is symmetric (self-adjoint, (maximal) dissipative, (maximal) accumulative) in*H*if and only if the closed linear relation Θ is symmetric (self-adjoint, (maximal) dissipative, (maximal) accumulative, respectively) in*G*.*

It is not surprising that Theorem 3.1 does not hold for quasi-boundary triples $\left\{G,\Gamma_{0},\Gamma_{1}\right\}$, see, e.g. [11, Proposition 4.11] for a counterexample. In particular, $\vartheta =\{0\}\times G_{1}\subset \mathrm{ran}\;\Gamma$ (see Definition 2.7 and Proposition 2.8(ii)) is symmetric and not self-adjoint in G but the corresponding extension $A_{\vartheta }$ of *A* in (3.1) coincides with the self-adjoint operator $A_{0}=T↾\ker\Gamma_{0}$ in H. Note that for a quasi-boundary triple $\left\{G,\Gamma_{0},\Gamma_{1}\right\}$ the range of the boundary map $\Gamma ={\left(\Gamma_{0},\Gamma_{1}\right)}^{⊤}$ is only dense in G×G, so that for a linear relation *ϑ* in G only the part ϑ∩ranΓ can be “detected” by the boundary maps. However, even for a self-adjoint linear relation ϑ⊂ranΓ the corresponding extension $A_{\vartheta }$ of *A* in (3.1) is in general not self-adjoint, see Example 4.22. Nevertheless, the following weaker statement is a direct consequence of the abstract Green's identity (2.1); cf. [11, Proposition 2.4].

Lemma 3.2 *Let*$\left\{G,\Gamma_{0},\Gamma_{1}\right\}$*be a quasi-boundary triple for*$T\subset A^{⁎}$*. Then the mapping*

$$\vartheta \mapsto A_{\vartheta }=T↾\{f\in \mathrm{dom}\;T:\Gamma f\in \vartheta \}$$

*establishes a bijective correspondence between the set of symmetric linear relations*ϑ⊂ranΓ*in*G*and the set of symmetric extensions*$A_{\vartheta }\subset T$*of A in*H*.*

We also mention that for a quasi-boundary triple $\left\{G,\Gamma_{0},\Gamma_{1}\right\}$ and linear relations θ⊂ϑ⊂ranΓ one has $A_{\theta }\subset A_{\vartheta }\subset T$; cf. (3.1).

In the next theorem we make use of a different type of parameterization to characterize the restrictions of *T* with the help of a quasi-boundary triple. The idea of the proof is to relate the given quasi-boundary triple $\left\{G,\Gamma_{0},\Gamma_{1}\right\}$ to the quasi-boundary triple in Lemma 2.5(ii) and to transform the parameters accordingly. We also point out that in contrast to most of the results in Section 2.3 here it is not assumed that the space $G_{1}=\mathrm{ran}(\Gamma_{1}↾\ker\Gamma_{0})$ is dense in G.

Theorem 3.3 *Let*$\left\{G,\Gamma_{0},\Gamma_{1}\right\}$*be a quasi-boundary triple for*$T\subset A^{⁎}$*with γ-field γ and Weyl function M. Assume that for*$A_{0}=T↾\ker\Gamma_{0}$*there exists*$\eta \in \rho (A_{0})\cap R$*and fix a unitary operator*$\varphi :N_{\eta }(A^{⁎})\to G$*. Then the mapping*

$$\Theta \mapsto A_{\vartheta }=T↾\{f\in \mathrm{dom}\;T:\Gamma f\in \vartheta \}\;\text{with}\vartheta =\gamma {\left(\eta \right)}^{⁎}\varphi^{⁎}\Theta \varphi \gamma (\eta )+M(\eta )$$

*establishes a bijective correspondence between all closed (symmetric, self-adjoint, (maximal) dissipative, (maximal) accumulative) linear relations Θ in*G*with*$\mathrm{dom}\;\Theta \subset \mathrm{ran}(\varphi ↾N_{\eta }(T))$*and all closed (symmetric, self-adjoint, (maximal) dissipative, (maximal) accumulative, respectively) extensions*$A_{\vartheta }\subset T$*of A in*H*.*

Proof Let *Θ* be a linear relation in G and decompose f∈domT in $f=f_{0}+f_{\eta }$, where $f_{0}\in \mathrm{dom}\;A_{0}$ and $f_{\eta }\in N_{\eta }(T)$. Then $\Gamma f\in \gamma {\left(\eta \right)}^{⁎}\varphi^{⁎}\Theta \varphi \gamma (\eta )+M(\eta )$ is equivalent to

$$\Gamma_{1}f=\gamma {\left(\eta \right)}^{⁎}\varphi^{⁎}x+M(\eta )\Gamma_{0}f\;\text{with }\left(\begin{array}{c} \varphi \gamma (\eta )\Gamma_{0}f\\ x \end{array}\right)\in \Theta ,$$

and by (2.6) this can be rewritten as

(3.2) $$\Gamma_{1}f_{0}=\gamma {\left(\eta \right)}^{⁎}\varphi^{⁎}x\;\text{with }\left(\begin{array}{c} \varphi f_{\eta }\\ x \end{array}\right)\in \Theta .$$

Denote the orthogonal projection in H onto $N_{\eta }(A^{⁎})$ by $P_{\eta }$. Making use of (2.4) and (2.5) we find

$$\Gamma_{1}f_{0}=\gamma {\left(\eta \right)}^{⁎}(A_{0}-\eta )f_{0}=\gamma {\left(\eta \right)}^{⁎}P_{\eta }(A_{0}-\eta )f_{0}$$

and as $\gamma {\left(\eta \right)}^{⁎}↾N_{\eta }(A^{⁎})$ is invertible we conclude together with (3.2)

(3.3) $$\Gamma f\in \gamma {\left(\eta \right)}^{⁎}\varphi^{⁎}\Theta \varphi \gamma (\eta )+M(\eta )\;\text{if and only if}\;\left(\begin{array}{c} \varphi f_{\eta }\\ \varphi P_{\eta }(A_{0}-\eta )f_{0} \end{array}\right)\in \Theta$$

for all $f=f_{0}+f_{\eta }\in \mathrm{dom}\;T$.

According to Proposition 2.3 and Lemma 2.5 the quasi-boundary triple $\left\{G,f\mapsto \varphi f_{\eta },f\mapsto \varphi P_{\eta }(A_{0}-\eta )f_{0}\right\}$ is the restriction of the ordinary boundary triple $\left\{G,f\mapsto \varphi f_{\eta },f\mapsto \varphi P_{\eta }(A_{0}-\eta )f_{0}\right\}$ for $A^{⁎}$. Now the statement is a consequence of Theorem 3.1. In fact, if e.g. *Θ* is self-adjoint in G with $\mathrm{dom}\;\Theta \subset \mathrm{ran}(\varphi ↾N_{\eta }(T))$, then by Theorem 3.1 the operator

(3.4) $$A^{⁎}↾\left\{f_{0}+f_{\eta }=\mathrm{dom}\;A_{0}∔N_{\eta }\left(A^{⁎}\right):\left(\begin{array}{c} \varphi f_{\eta }\\ \varphi P_{\eta }(A_{0}-\eta )f_{0} \end{array}\right)\in \Theta \right\}$$

is a self-adjoint restriction of $A^{⁎}$ in H. As $\mathrm{dom}\;\Theta \subset \mathrm{ran}(\varphi ↾N_{\eta }(T))$ we conclude that the domain of the operator in (3.4) is contained in dom *T*. Hence by (3.3) the operator in (3.4) can be written as

(3.5) $$A_{\vartheta }=T↾\{f\in \mathrm{dom}\;T:\Gamma f\in \vartheta \}\;\text{with }\vartheta =\gamma {\left(\eta \right)}^{⁎}\varphi^{⁎}\Theta \varphi \gamma (\eta )+M(\eta )$$

and $A_{\vartheta }$ is a self-adjoint operator in H. Conversely, by Theorem 3.1 for any self-adjoint extension $A_{\vartheta }$ of *A* which is contained in *T* there exists a self-adjoint relation *Θ* in G such that $A_{\vartheta }$ can be written in the form (3.4), where $N_{\eta }(A^{⁎})$ can be replaced by $N_{\eta }(T)$. Therefore $\mathrm{dom}\;\Theta \subset \mathrm{ran}(\varphi ↾N_{\eta }(T))$ and together with (3.3) we conclude that $A_{\vartheta }$ can be written in the form (3.5).  □

The next theorem is of similar flavor as Theorem 3.3 but more explicit and relevant for elliptic boundary value problems; cf. Section 4. Under the additional assumption that the space $G_{1}=\mathrm{ran}(\Gamma_{1}↾\ker\Gamma_{0})$ in Definition 2.7 is dense in G a more natural parameterization of the extensions is found. Here we will again make use of the Gelfand triple $G_{1}↪G↪G_{1}^{\prime }$ and the corresponding isometric isomorphisms $\iota_{+}$ and $\iota_{-}$ in (2.15). We also note that after suitable modifications the assumption η∈R can be dropped, see Remark 2.13.

Theorem 3.4 *Let*$\left\{G,\Gamma_{0},\Gamma_{1}\right\}$*be a quasi-boundary triple for*$T\subset A^{⁎}$*with*$A_{0}=T↾\ker\Gamma_{0}$*and Weyl function M. Assume that there exists*$\eta \in \rho (A_{0})\cap R$*and that*$G_{1}$*is dense in*G*. Then the mapping*

$$\Theta \mapsto A_{\vartheta }=T↾\{f\in \mathrm{dom}\;T:\Gamma f\in \vartheta \}\;\text{with}\vartheta =\iota_{+}^{-1}\Theta \iota_{-}+M(\eta )$$

*establishes a bijective correspondence between all closed (symmetric, self-adjoint, (maximal) dissipative, (maximal) accumulative) linear relations Θ in*G*with*$\mathrm{dom}\;\Theta \subset \mathrm{ran}\;\iota_{-}\Gamma_{0}$*and all closed (symmetric, self-adjoint, (maximal) dissipative, (maximal) accumulative, respectively) extensions*$A_{\vartheta }\subset T$*of A in*H*.*

Proof Let *Θ* be a linear relation in G and decompose f∈domT in the form $f=f_{0}+f_{\eta }$ with $f_{0}\in \mathrm{dom}\;A_{0}$ and $f_{\eta }\in N_{\eta }(T)$. Then $\Gamma f\in \iota_{+}^{-1}\Theta \iota_{-}+M(\eta )$ if and only if

(3.6) $$\Gamma_{1}f=\iota_{+}^{-1}x+M(\eta )\Gamma_{0}f\;\text{with }\left(\begin{array}{c} \iota_{-}\Gamma_{0}f\\ x \end{array}\right)\in \Theta .$$

Eq. (2.6) implies $\Gamma_{1}f-M(\eta )\Gamma_{0}f=\Gamma_{1}f_{0}$ and since f∈domT we have $\Gamma_{0}f={\overset{˜}{\Gamma }}_{0}f$, where ${\overset{˜}{\Gamma }}_{0}$ is the continuous extension of $\Gamma_{0}$ to $\mathrm{dom}\;A^{⁎}$ from Proposition 2.10. Hence (3.6) is equivalent to

(3.7) $$\left(\begin{array}{c} \iota_{-}{\overset{˜}{\Gamma }}_{0}f\\ \iota_{+}\Gamma_{1}f_{0} \end{array}\right)\in \Theta .$$

According to Theorem 2.12 the triple $\left\{G,\;f\mapsto \iota_{-}{\overset{˜}{\Gamma }}_{0}f,\;f\mapsto \iota_{+}\Gamma_{1}f_{0}\right\}$ is an ordinary boundary triple for $A^{⁎}$. Now the statement follows from Theorem 3.1 and the same reasoning as in the proof of Theorem 3.3.  □

Corollary 3.5 *Let the assumptions be as in* Theorem 3.4 *and let ϑ be a linear relation in* G *. Then the extension* $A_{\vartheta }$ *of A in* H *given by*

(3.8) $$A_{\vartheta }=T↾\{f\in \mathrm{dom}\;T:\Gamma f\in \vartheta \}$$

*is closed (symmetric, self-adjoint, (maximal) dissipative, (maximal) accumulative) in* H *if and only if the linear relation*

$$\Theta =\iota_{+}\left(\vartheta -M(\eta )\right)\iota_{-}^{-1}\;\text{with}\mathrm{dom}\;\Theta \subset \mathrm{ran}\;\iota_{-}\Gamma_{0}$$

*is closed (symmetric, self-adjoint, (maximal) dissipative, (maximal) accumulative) in* G *.*

Proof (⇒) Assume that $A_{\vartheta }$ in (3.8) is a closed (symmetric, self-adjoint, (maximal) dissipative, (maximal) accumulative) operator in H. According to Theorem 3.4 there exists a closed (symmetric, self-adjoint, (maximal) dissipative, (maximal) accumulative, respectively) linear relation *Θ* in G with $\mathrm{dom}\;\Theta \subset \mathrm{ran}\;\iota_{-}\Gamma_{0}$ and

(3.9) $$A_{\vartheta }=A_{\theta }=T↾\{f\in \mathrm{dom}\;T:\Gamma f\in \theta \}\;\text{with }\theta =\iota_{+}^{-1}\Theta \iota_{-}+M(\eta ).$$

From $\iota_{+}^{-1}\Theta \iota_{-}\subset \mathrm{ran}\;\Gamma_{0}\times G_{1}$ and Proposition 2.8(ii) we conclude θ⊂ranΓ. Furthermore, we have θ=ϑ∩ranΓ, (see the text below Lemma 3.2). Solving Eq. (3.9) leads to the identity

$$\Theta =\iota_{+}\left(\theta -M(\eta )\right)\iota_{-}^{-1}=\iota_{+}\left(\vartheta -M(\eta )\right)\iota_{-}^{-1}.$$

(⇐) Let $\Theta =\iota_{+}(\vartheta -M(\eta ))\iota_{-}^{-1}$ with $\mathrm{dom}\;\Theta \subset \mathrm{ran}\;\iota_{-}\Gamma_{0}$ be a closed (symmetric, self-adjoint, (maximal) dissipative, (maximal) accumulative) linear relation in G. From $\vartheta -M(\eta )=\iota_{+}^{-1}\Theta \iota_{-}\subset \mathrm{ran}\;\Gamma_{0}\times G_{1}$ and Proposition 2.8(ii) we obtain $\theta =\iota_{+}^{-1}\Theta \iota_{-}+M(\eta )$ with θ=ϑ∩ranΓ. According to Theorem 3.4 the extension $A_{\theta }=A_{\vartheta }$ given by (3.8) is closed (symmetric, self-adjoint, (maximal) dissipative, (maximal) accumulative) in H.  □

We recall that a symmetric linear relation *Θ* in G with ranΘ=G is self-adjoint in G with 0∈ρ(Θ). This together with Corollary 3.5 yields the following example.

Example 3.6 Let the assumptions be as in Corollary 3.5 and let *ϑ* be a symmetric linear relation in G such that $\mathrm{ran}(\vartheta -M(\eta ))=G_{1}$. Then

$$A_{\vartheta }=T↾\{f\in \mathrm{dom}\;T:\Gamma f\in \vartheta \}$$

is a self-adjoint extension of *A* in H.

In the next result the assumptions on the quasi-boundary triple are strengthened further such that both boundary maps $\Gamma_{0}$ and $\Gamma_{1}$ extend by continuity to $\mathrm{dom}\;A^{⁎}$. In that case one obtains a description of all extensions $A_{\vartheta }\subset A^{⁎}$ which is very similar to the parameterization in Theorem 3.4. The additional abstract regularity result will turn out to be useful when considering the regularity of solutions of elliptic boundary value problems in Section 4.

Theorem 3.7 *Let the assumptions be as in Theorem*3.4*and assume, in addition, that*$A_{1}=T↾\ker\Gamma_{1}$*is self-adjoint in*H*,*$\eta \in \rho (A_{0})\cap \rho (A_{1})\cap R$*, and that*$G_{0}$*dense in*G*. Let*$\overset{˜}{M}$*be the extension of the Weyl function M from*Definition 2.14(ii)*. Then the mapping*

$$\Theta \mapsto {\overset{˜}{A}}_{\vartheta }=A^{⁎}↾\left\{f\in \mathrm{dom}\;A^{⁎}:\overset{˜}{\Gamma }f\in \vartheta \right\}\;\text{with}\vartheta =\iota_{+}^{-1}\Theta \iota_{-}+\overset{˜}{M}(\eta )$$

*establishes a bijective correspondence between all closed (symmetric, self-adjoint, (maximal) dissipative, (maximal) accumulative) linear relations Θ in*G*and all closed (symmetric, self-adjoint, (maximal) dissipative, (maximal) accumulative, respectively) extensions*${\overset{˜}{A}}_{\vartheta }\subset A^{⁎}$*of A in*H*.*

*Moreover, the following abstract regularity result holds: If Θ is a linear relation in* G *and S is an operator in* H *such that* $T\subset S\subset A^{⁎}$ *then*

$$\mathrm{dom}\;\Theta \subset \mathrm{ran}(\iota_{-}{\overset{˜}{\Gamma }}_{0}↾\mathrm{dom}\;S)\;\text{implies}\mathrm{dom}\;{\overset{˜}{A}}_{\vartheta }\subset \mathrm{dom}\;S.$$

Proof The proof of the first part is very similar to the proof of Theorem 3.4 and will not be repeated here. We show the abstract regularity result. Let *Θ* and *S* be as in the theorem and assume that dom *Θ* is contained in the range of the map $\iota_{-}{\overset{˜}{\Gamma }}_{0}↾\mathrm{dom}\;S$. Let

$${\overset{˜}{A}}_{\vartheta }=A^{⁎}↾\left\{f\in \mathrm{dom}\;A^{⁎}:\overset{˜}{\Gamma }f\in \iota_{+}^{-1}\Theta \iota_{-}+\overset{˜}{M}(\eta )\right\}$$

be the corresponding extension and let $f\in \mathrm{dom}\;{\overset{˜}{A}}_{\vartheta }$. As $\overset{˜}{\Gamma }f\in \iota_{+}^{-1}\Theta \iota_{-}+\overset{˜}{M}(\eta )$ we have $\iota_{-}{\overset{˜}{\Gamma }}_{0}f\in \mathrm{dom}\;\Theta$. Since $\mathrm{dom}\;\Theta \subset \mathrm{ran}(\iota_{-}{\overset{˜}{\Gamma }}_{0}↾\mathrm{dom}\;S)$ there exists an element g∈domS such that $\iota_{-}{\overset{˜}{\Gamma }}_{0}f=\iota_{-}{\overset{˜}{\Gamma }}_{0}g$ holds. Hence we conclude $f-g\in \ker{\overset{˜}{\Gamma }}_{0}=\mathrm{dom}\;A_{0}\subset \mathrm{dom}\;S$, so that f=g+(f−g)∈domS.  □

The next corollary is a counterpart of Corollary 3.5 and can be proved in the same way using Lemma 2.16(v) instead of Proposition 2.8(ii).

Corollary 3.8 *Let the assumptions be as in Theorem* 3.7 *and let ϑ be a linear relation in* $G_{1}^{\prime }\times G_{0}^{\prime }$ *. Then the extension* ${\overset{˜}{A}}_{\vartheta }$ *of A in* H *given by*

$${\overset{˜}{A}}_{\vartheta }=A^{⁎}↾\left\{f\in \mathrm{dom}\;A^{⁎}:\overset{˜}{\Gamma }f\in \vartheta \right\}$$

*is closed (symmetric, self-adjoint, (maximal) dissipative, (maximal) accumulative) in* H *if and only if the linear relation*

$$\Theta =\iota_{+}\left(\vartheta -\overset{˜}{M}(\eta )\right)\iota_{-}^{-1}$$

*is closed (symmetric, self-adjoint, (maximal) dissipative, (maximal) accumulative) in* G *.*

A simple application of Theorem 3.7 is discussed in the next example.

Example 3.9 Set Θ=0 in Theorem 3.7. Then $\vartheta =\overset{˜}{M}(\eta )$ and it follows that

$${\overset{˜}{A}}_{\vartheta }=A^{⁎}↾\left\{f\in \mathrm{dom}\;A^{⁎}:\overset{˜}{M}(\eta ){\overset{˜}{\Gamma }}_{0}f={\overset{˜}{\Gamma }}_{1}f\right\}$$

is a self-adjoint extension of *A* in H. From Lemma 2.16(ii) we obtain that the condition $\overset{˜}{M}(\eta ){\overset{˜}{\Gamma }}_{0}f={\overset{˜}{\Gamma }}_{1}f$ is equivalent to $\Gamma_{1}f_{0}=0$, where $f=f_{0}+f_{\eta }\in \mathrm{dom}\;A_{0}∔N_{\eta }(A^{⁎})$. This implies that ${\overset{˜}{A}}_{\vartheta }=A∔{\overset{ˆ}{N}}_{\eta }(A^{⁎})$, which coincides with the Kreĭn–von Neumann extension if *A* is uniformly positive and η=0; cf. [51] and, e.g. [47].

### 3.2 Sufficient conditions for self-adjointness and a variant of Kreĭn's formula

In this subsection we provide different sufficient conditions for the parameter *ϑ* in G×G such that the corresponding extension

$$A_{\vartheta }=T↾\{f\in \mathrm{dom}\;T:\Gamma f\in \vartheta \},\;\vartheta =\iota_{+}^{-1}\Theta \iota_{-}+M(\eta ),$$

in Theorem 3.4 becomes self-adjoint in H; cf. [11, Theorem 4.8], [13, Theorem 3.11] and, e.g. Example 3.6. In Proposition 3.10 below we will make use of standard perturbation results, such as the Kato–Rellich theorem. Thus we will restrict ourselves to operators *ϑ* instead of relations. Recall also the following notions from perturbation theory: If M is a linear operator acting between two Banach spaces then a sequence ${\left(x_{k}\right)}_{k\in N}\subset \mathrm{dom}\;M$ is called M*-bounded* if ${\left(x_{k}\right)}_{k\in N}$ is bounded with respect to the graph norm of M. A linear operator *θ* is said to *relatively compact* with respect to M if domM⊂domθ and *θ* maps M-bounded sequences into sequences which have convergent subsequences.

Proposition 3.10 *Let* $\left\{G,\Gamma_{0},\Gamma_{1}\right\}$ *be a quasi-boundary triple for* $T\subset A^{⁎}$ *with* $A_{j}=T↾\ker\Gamma_{j}$ *,* j=0,1 *, and Weyl function M, and assume that* $A_{1}$ *is self-adjoint in* H *and that there exists* $\eta \in \rho (A_{0})\cap \rho (A_{1})\cap R$ *. Furthermore, suppose that* $G_{0}$ *and* $G_{1}$ *are dense in* G *and equip* $G_{0}$ *and* $G_{1}$ *with norms* ${\left\|\cdot \right\|}_{G_{0}}$ *and* ${\left\|\cdot \right\|}_{G_{1}}$ *such that both* $\left(G_{0},{\left\|\cdot \right\|}_{G_{0}}\right)$ *and* $\left(G_{1},{\left\|\cdot \right\|}_{G_{1}}\right)$ *are reflexive Banach spaces continuously embedded in* G *.*

*If ϑ is a symmetric operator in*G*such that*

(3.10) $$G_{0}\subset \mathrm{dom}\;\vartheta \;\text{and}\;\mathrm{ran}\;\vartheta ↾G_{0}\subset G_{1},$$

*and one of the followings conditions* (i)–(iii) *hold,*

(i) *ϑ regarded as an operator from*$G_{0}$*to*$G_{1}$*is compact,*
(ii) *ϑ regarded as an operator from*$G_{0}$*to*$G_{1}$*is relatively compact with respect to*M(η)*regarded as an operator from*$G_{0}$*to*$G_{1}$*,*
(iii) *there exist*$c_{1}>0$*and*$c_{2}\in [0,1)$*such that*
  $${\left\|\vartheta x\right\|}_{G_{1}}\leq c_{1}{\left\|x\right\|}_{G_{1}^{\prime }}+c_{2}{\left\|M(\eta )x\right\|}_{G_{1}},\;x\in G_{0},$$

*then* $A_{\vartheta }=T↾\{f\in \mathrm{dom}\;T:\Gamma f\in \vartheta \}$ *is self-adjoint in* H*.*

Proof Note first that condition (i) is a special case of condition (ii). Hence it suffices to prove the proposition under conditions (ii) or (iii). By (3.10) the restriction $\theta :=\vartheta ↾G_{0}$ maps into $G_{1}$ and the corresponding extensions of *A* in H satisfy $A_{\theta }\subset A_{\vartheta }$. We show below that (ii) or (iii) imply the self-adjointness of $A_{\theta }$ and hence, as $A_{\vartheta }$ is symmetric by Lemma 3.2, the self-adjointness of $A_{\vartheta }$.

By Corollary 3.5 the operator $A_{\theta }=T↾\{f\in \mathrm{dom}\;T:\Gamma f\in \theta \}$ is self-adjoint in H if and only if $\Theta =\iota_{+}(\theta -M(\eta ))\iota_{-}^{-1}$ is self-adjoint in G. Since *ϑ* is assumed to be a symmetric operator the same holds for *θ*, $\iota_{+}\theta \iota_{-}^{-1}$ and *Θ*. From Lemma 2.16(iv) we obtain that $M:=M(\eta )↾G_{0}$ is an isomorphism onto $G_{1}$. Thus the symmetric operator $-\iota_{+}M\iota_{-}^{-1}$ defined on $\iota_{-}G_{0}$ is surjective and hence self-adjoint in G. Therefore

(3.11) $$\Theta =\iota_{+}(\theta -M)\iota_{-}^{-1}=-\iota_{+}M\iota_{-}^{-1}+\iota_{+}\theta \iota_{-}^{-1}$$

can be regarded as an additive symmetric perturbation of the self-adjoint operator $-\iota_{+}M\iota_{-}^{-1}$, and the assertion of the proposition holds if we show that *Θ* is self-adjoint in G.

Assume first that condition (ii) holds, that is, *θ* is relatively compact with respect to M, and hence also with respect to −M. Making use of the fact that $\iota_{+}:G_{1}\to G$ and $\iota_{-}:G_{1}^{\prime }\to G$ are isometric isomorphisms it is not difficult to verify that $\iota_{+}\theta \iota_{-}^{-1}$ is relatively compact with respect to $-\iota_{+}M\iota_{-}^{-1}$ in G. Hence by well known perturbation results the operator *Θ* in (3.11) is self-adjoint in G, see, e.g. [73, Theorem 9.14].

Suppose now that (iii) holds and set $\xi =\iota_{-}x$ for $x\in G_{0}$. Then

$${\left\|\iota_{+}\theta \iota_{-}^{-1}\xi \right\|}_{G}={\left\|\theta x\right\|}_{G_{1}}\leq c_{1}{\left\|x\right\|}_{G_{1}^{\prime }}+c_{2}{\left\|Mx\right\|}_{G_{1}}=c_{1}{\left\|\xi \right\|}_{G}+c_{2}{\left\|\iota_{+}M\iota_{-}^{-1}\xi \right\|}_{G}$$

shows that the symmetric operator $\iota_{+}\theta \iota_{-}^{-1}$ is $\iota_{+}M\iota_{-}^{-1}$-bounded with a relative bound $c_{2}<1$. Hence the Kato–Rellich theorem [65, Theorem X.12] implies that *Θ* in (3.11) is a self-adjoint operator in G.  □

The next proposition is of the same flavor as Proposition 3.10. It can be proved similarly with the help of a variant of the Kato–Rellich theorem due to Wüst; cf. [65, Theorem X.14] and [77].

Proposition 3.11 *Let the assumptions be as in Proposition* 3.10 *and assume that there exists* c>0 *such that*

$${\left\|\vartheta x\right\|}_{G_{1}}\leq c{\left\|x\right\|}_{G_{1}^{\prime }}+{\left\|M(\eta )x\right\|}_{G_{1}},\;x\in G_{0}.$$

*Then* $A_{\vartheta }=T↾\{f\in \mathrm{dom}\;T:\Gamma f\in \vartheta \}$ *is essentially self-adjoint in* H *.*

Example 3.12 Let *ϑ* be a symmetric operator in G with $G_{0}\subset \mathrm{dom}\;\vartheta$, such that *ϑ* is continuous from $\left(G_{0},\;{\left\|\cdot \right\|}_{G_{1}^{\prime }}\right)$ to $G_{1}$. Then condition (iii) in Proposition 3.10 is satisfied with $c_{2}=0$ and hence the extension $A_{\vartheta }$ of *A* is self-adjoint in H.

Now consider $\vartheta :=M(\eta )↾G_{0}$ as an operator from $G_{0}$ to $G_{1}$. Then Proposition 3.11 implies that $A_{\vartheta }$ is essentially self-adjoint in H. In fact, as in Example 3.9 one verifies $A_{\vartheta }=A∔{\overset{ˆ}{N}}_{\eta }(T)$, which is a proper restriction of ${\overset{˜}{A}}_{\vartheta }=A∔{\overset{ˆ}{N}}_{\eta }(A^{⁎})$ from Example 3.9.

For completeness we provide a version of Kreĭn's formula for quasi-boundary triples in Corollary 3.14 which can be viewed as a direct consequence of Kreĭn's formula for the ordinary boundary triple in Theorem 2.12. A similar type of resolvent formula can also be found in [26, Theorem 7.26] for generalized boundary triples. For the convenience of the reader we first recall Kreĭn's formula for ordinary boundary triples, see, e.g. [27]. The point, continuous and residual spectrum of a closed linear relation is defined in the same way a for a closed linear operator; cf. [29], [30].

Theorem 3.13 *Let*$\left\{G,\Gamma_{0},\Gamma_{1}\right\}$*be an ordinary boundary triple for*$A^{⁎}$*with γ-field γ and Weyl function M and*$A_{0}=A^{⁎}↾\ker\Gamma_{0}$*, let Θ be a closed linear relation in*G*and let*$A_{\Theta }$*be the corresponding closed extension in*Theorem 3.1*. Then for all*$\lambda \in \rho (A_{0})$*the following assertions* (i)–(iv) *hold.*

(i) $\lambda \in \sigma_{p}(A_{\Theta })$*if and only if*$0\in \sigma_{p}(\Theta -M(\lambda ))$*, in this case*
  $$\ker(A_{\Theta }-\lambda )=\gamma (\lambda )\ker\left(\Theta -M(\lambda )\right),$$
(ii) $\lambda \in \sigma_{c}(A_{\Theta })$*if and only if*$0\in \sigma_{c}(\Theta -M(\lambda ))$*,*
(iii) $\lambda \in \sigma_{r}(A_{\Theta })$*if and only if*$0\in \sigma_{r}(\Theta -M(\lambda ))$*,*
(iv) $\lambda \in \rho (A_{\Theta })$*if and only if*0∈ρ(Θ−M(λ))*and the formula*
  $${\left(A_{\Theta }-\lambda \right)}^{-1}={\left(A_{0}-\lambda \right)}^{-1}+\gamma (\lambda ){\left(\Theta -M(\lambda )\right)}^{-1}\gamma {\left(\overset{¯}{\lambda }\right)}^{⁎}$$
  *holds for all*$\lambda \in \rho (A_{0})\cap \rho (A_{\Theta })$*.*

The next corollary contains a variant of Kreĭn's formula for quasi-boundary triples; cf. [11, Theorem 2.8], [13, Theorem 3.6], and [12, Theorem 6.16] for other versions of Kreĭn's formula for the resolvent difference of canonical extensions in the quasi-boundary triple framework.

Corollary 3.14 *Let*$\left\{G,\Gamma_{0},\Gamma_{1}\right\}$*be a quasi-boundary triple for*$T\subset A^{⁎}$*with γ-field γ, Weyl function M,*$A_{j}=T↾\ker\Gamma_{j}$*,*j=0,1*, such that*$A_{1}$*is self-adjoint in*H*, there exists*$\eta \in \rho (A_{0})\cap R$*and*$G_{0},\;G_{1}$*are dense in*G*. Equip*$G_{0}$*and*$G_{1}$*with norms*${\left\|\cdot \right\|}_{G_{0}}$*and*${\left\|\cdot \right\|}_{G_{1}}$*such that both*$\left(G_{0},{\left\|\cdot \right\|}_{G_{0}}\right)$*and*$\left(G_{1},{\left\|\cdot \right\|}_{G_{1}}\right)$*are reflexive Banach spaces continuously embedded in*G*, and let*$\overset{˜}{\gamma }$*and*$\overset{˜}{M}$*be the extensions of γ and M, respectively. Moreover let*$\vartheta \subset G_{1}^{\prime }\times G_{0}^{\prime }$*be a linear relation in*$\mathrm{ran}\;\overset{˜}{\Gamma }$*such that the extension*

$${\overset{˜}{A}}_{\vartheta }=A^{⁎}↾\left\{f\in \mathrm{dom}\;A^{⁎}:\overset{˜}{\Gamma }f\in \vartheta \right\}$$

*is closed in*H*. Then for all*$\lambda \in \rho (A_{0})$*the following assertions* (i)–(iv) *hold.*

(i) $\lambda \in \sigma_{p}({\overset{˜}{A}}_{\vartheta })$*if and only if*$0\in \sigma_{p}(\iota_{+}(\vartheta -\overset{˜}{M}(\lambda ))\iota_{-}^{-1})$*, in this case*
  $$\ker({\overset{˜}{A}}_{\vartheta }-\lambda )=\overset{˜}{\gamma }(\lambda )\ker\left(\vartheta -\overset{˜}{M}(\lambda )\right),$$
(ii) $\lambda \in \sigma_{c}({\overset{˜}{A}}_{\vartheta })$*if and only if*$0\in \sigma_{c}(\iota_{+}(\vartheta -\overset{˜}{M}(\lambda ))\iota_{-}^{-1})$*,*
(iii) $\lambda \in \sigma_{r}({\overset{˜}{A}}_{\vartheta })$*if and only if*$0\in \sigma_{r}(\iota_{+}(\vartheta -\overset{˜}{M}(\lambda ))\iota_{-}^{-1})$*,*
(iv) $\lambda \in \rho ({\overset{˜}{A}}_{\vartheta })$*if and only if*$0\in \rho (\iota_{+}(\vartheta -\overset{˜}{M}(\lambda ))\iota_{-}^{-1})$*and*
  $${\left({\overset{˜}{A}}_{\vartheta }-\lambda \right)}^{-1}={\left(A_{0}-\lambda \right)}^{-1}+\overset{˜}{\gamma }(\lambda ){\left(\vartheta -\overset{˜}{M}(\lambda )\right)}^{-1}\overset{˜}{\gamma }{\left(\overset{¯}{\lambda }\right)}^{\prime }$$
  *holds for all*$\lambda \in \rho ({\overset{˜}{A}}_{\vartheta })\cap \rho (A_{0})$*.*

Proof Let $\left\{G,{ϒ}_{0},{ϒ}_{1}\right\}$ be the ordinary boundary triple for $A^{⁎}$ in Theorem 2.12 with $A_{0}=A^{⁎}↾\ker{ϒ}_{0}$, *γ*-field *β* and corresponding Weyl function M in (2.17). By assumption we have $\vartheta \subset \mathrm{ran}\;\overset{˜}{\Gamma }$. According to Corollary 3.8 the linear relation $\Theta =\iota_{+}(\vartheta -\overset{˜}{M}(\eta ))\iota_{-}^{-1}$ is closed in G and it follows that ${\overset{˜}{A}}_{\vartheta }$ and

$$A_{\Theta }=A^{⁎}↾\left\{f\in \mathrm{dom}\;A^{⁎}:ϒf\in \Theta \right\}$$

coincide. Since $M(\lambda )=\iota_{+}(\overset{˜}{M}(\lambda )-\overset{˜}{M}(\eta ))\iota_{-}^{-1}$ by (2.17) we obtain the identity $\Theta -M(\lambda )=\iota_{+}(\vartheta -\overset{˜}{M}(\lambda ))\iota_{-}^{-1}$ and from $\beta (\lambda )=\overset{˜}{\gamma }(\lambda )\iota_{-}^{-1}$ and $\beta {\left(\overset{¯}{\lambda }\right)}^{⁎}=\iota_{+}\overset{˜}{\gamma }{\left(\overset{¯}{\lambda }\right)}^{\prime }$ we then conclude

(3.12) $$\beta (\lambda ){\left(\Theta -M(\lambda )\right)}^{-1}\beta {\left(\overset{¯}{\lambda }\right)}^{⁎}=\overset{˜}{\gamma }(\lambda ){\left(\vartheta -\overset{˜}{M}(\lambda )\right)}^{-1}\overset{˜}{\gamma }{\left(\overset{¯}{\lambda }\right)}^{\prime }.$$

Now the assertions follow from Theorem 3.13, ${\overset{˜}{A}}_{\vartheta }=A_{\Theta }$ and (3.12). Note that ${\left(\vartheta -\overset{˜}{M}(\lambda )\right)}^{-1}\subset G_{1}\times G_{1}^{\prime }$ in (3.12) since $\vartheta -\overset{˜}{M}(\lambda )\subset G_{1}^{\prime }\times G_{1}$ by Lemma 2.16(v).  □

## 4 Applications to elliptic boundary value problems

In this section the abstract theory from Section 2 and Section 3 is applied to elliptic differential operators. In Section 4.1 we first study the Laplacian on bounded Lipschitz-, quasi-convex and $C^{1,r}$-domains with $r\in (\frac{1}{2},1]$. Then we investigate 2*m*-th order elliptic differential operators on bounded smooth domains in Section 4.2 and second order elliptic differential operators on domains with compact boundary in Section 4.3.

Throughout this section let $\Omega \subset R^{n}$, n≥2, be a domain with boundary ∂*Ω* (which is at least Lipschitz). In Section 4.1 and Section 4.2 the domain *Ω* is assumed to be bounded, in Section 4.3 the domain *Ω* may be unbounded as well but its boundary ∂*Ω* is assumed to be compact. We denote by $H^{s}(\Omega )$ the Sobolev spaces of order s∈R on *Ω* and by $H^{s}(\partial \Omega )$ the Sobolev spaces on ∂*Ω* of order *s* (with at least s∈[−1,1] in the Lipschitz case). By $H_{0}^{s}(\Omega )$ we denote the closure of $C_{0}^{\infty }(\Omega )$ in $H^{s}(\Omega )$, s≥0, and with $C^{\infty }(\bar{\Omega })$ the functions in $C_{0}^{\infty }(R^{n})$ restricted to *Ω*; see, e.g. [57, Chapter 3].

### 4.1 A description of all self-adjoint extensions of the Laplacian on bounded Lipschitz domains

In this subsection we give a complete description of the self-adjoint extensions of the Laplacian $-\Delta =-\sum_{j=1}^{n}\partial_{j}^{2}$ on a bounded Lipschitz domain *Ω* in terms of linear operators and relations *Θ* in $L^{2}(\partial \Omega )$ with the help of Theorem 3.7. This description extends the one by Gesztesy and Mitrea in [36], where the class of so-called quasi-convex domains was treated; cf. [36, Definition 8.9]. In addition we introduce Hilbert spaces $G_{0}$ and $G_{1}$ such that the Dirichlet- and Neumann trace operator admit continuous and surjective extensions from the maximal domain of the Laplacian onto the anti-dual spaces $G_{1}^{\prime }$ and $G_{0}^{\prime }$ respectively.

Let $\Omega \subset R^{n}$, n≥2, be a bounded Lipschitz domain. For s≥0 we define the Hilbert spaces

$$H_{\Delta }^{s}(\Omega ):=\left\{f\in H^{s}(\Omega ):\Delta f\in L^{2}(\Omega )\right\}$$

equipped with the norms induced by

$${\left(f,\;g\right)}_{H_{\Delta }^{s}(\Omega )}:={\left(f,g\right)}_{H^{s}(\Omega )}+{\left(\Delta f,\Delta g\right)}_{L^{2}(\Omega )},\;f,g\in H_{\Delta }^{s}(\Omega ).$$

Note that for s≥2 the spaces $H_{\Delta }^{s}(\Omega )$ coincide with $H^{s}(\Omega )$. Define the minimal and maximal realization of the Laplacian in $L^{2}(\Omega )$ by

$$\Delta_{\min}:=-\Delta ↾H_{0}^{2}(\Omega )\;\text{and}\;\Delta_{\max}:=-\Delta ↾H_{\Delta }^{0}(\Omega ),$$

respectively, and let $A:=\Delta_{\min}$. It follows from the Poincaré inequality that the norm induced by $H_{\Delta }^{0}(\Omega )$ is equivalent to the $H^{2}$-norm on $H_{0}^{2}(\Omega )$. Hence a usual distribution type argument yields

$$A=\Delta_{\min}=\Delta_{\max}^{⁎}\;\text{and}\;A^{⁎}=\Delta_{\min}^{⁎}=\Delta_{\max};$$

cf. [70, VI. § 29]. We mention that *A* is a closed, densely defined, symmetric operator in $L^{2}(\Omega )$ with equal infinite deficiency indices. Let $n={\left(n_{1},\;n_{2},\;\ldots ,\;n_{n}\right)}^{⊤}$ be the unit vector field pointing out of *Ω*, which exists almost everywhere, see, e.g. [57], [76]. The Dirichlet and Neumann trace operator $\tau_{D}$ and $\tau_{N}$ defined by

$$\tau_{D}f:=f{↾}_{\partial \Omega },\;\tau_{N}f:=n\cdot \nabla f{↾}_{\partial \Omega },\;f\in C^{\infty }(\bar{\Omega }),$$

admit continuous extensions to operators

(4.1) $$\tau_{D}:H_{\Delta }^{s}(\Omega )\to H^{s-1/2}(\partial \Omega )\;\text{and}\;\tau_{N}:H_{\Delta }^{s}(\Omega )\to H^{s-3/2}(\partial \Omega )$$

for all $s\in [\frac{1}{2},\frac{3}{2}]$. In particular, according to [36, Lemma 3.1 and Lemma 3.2] the extensions $\tau_{D}$ and $\tau_{N}$ in (4.1) are both surjective if $s=\frac{1}{2}$ and $s=\frac{3}{2}$.

In the next theorem we define a quasi-boundary triple for the Laplacian

(4.2) $$T:=-\Delta ↾H_{\Delta }^{3/2}(\Omega )=A^{⁎}↾H_{\Delta }^{3/2}(\Omega )\subset \Delta_{\max}$$

on the bounded Lipschitz domain *Ω* with $\Gamma_{0}$ and $\Gamma_{1}$ as the natural trace maps. In this setting it turns out that the spaces $G_{0}$ and $G_{1}$ from Definition 2.7 are dense in $L^{2}(\partial \Omega )$, the *γ*-field coincides with a family of Poisson operators and the values of the Weyl function are Dirichlet-to-Neumann maps (up to a minus sign).

Theorem 4.1 *Let Ω be a bounded Lipschitz domain, let T be as in*(4.2)*and let*

$$\Gamma_{0},\Gamma_{1}:H_{\Delta }^{3/2}(\Omega )\to L^{2}(\partial \Omega ),\;\Gamma_{0}f:=\tau_{D}f,\;\Gamma_{1}f:=-\tau_{N}f.$$

*Then*$\left\{L^{2}(\partial \Omega ),\Gamma_{0},\Gamma_{1}\right\}$*is a quasi-boundary triple for*$T\subset A^{⁎}=\Delta_{\max}$*such that the minimal realization*$A=\Delta_{\min}$*coincides with*T↾ker⁡Γ*and the following statements hold.*

(i) *The Dirichlet realization*$\Delta_{D}$*and Neumann realization*$\Delta_{N}$*correspond to*$\ker\Gamma_{0}$*and*$\ker\Gamma_{1}$*,*
  (4.3) $$\begin{array}{ccc} \Delta_{D} & := & T↾\ker\Gamma_{0}=\Delta_{\max}↾\left\{f\in H_{\Delta }^{3/2}(\Omega ):\tau_{D}f=0\right\},\\ \Delta_{N} & := & T↾\ker\Gamma_{1}=\Delta_{\max}↾\left\{f\in H_{\Delta }^{3/2}(\Omega ):\tau_{N}f=0\right\}, \end{array}$$
  *respectively, and both operators are self-adjoint in*$L^{2}(\Omega )$*.*
(ii) *The spaces*
  $$G_{0}=\mathrm{ran}(\Gamma_{0}↾\ker\Gamma_{1})\;\text{and}\;G_{1}=\mathrm{ran}(\Gamma_{1}↾\ker\Gamma_{0})$$
  *are dense in*$L^{2}(\partial \Omega )$*.*
(iii) *The values*$\gamma (\lambda ):L^{2}(\partial \Omega )⊃H^{1}(\partial \Omega )\to L^{2}(\Omega )$*of the γ-field are given by*
  $$\gamma (\lambda )\varphi =f,\;\varphi \in H^{1}(\partial \Omega ),\;\lambda \in \rho (\Delta_{D}),$$
  *where*$f\in L^{2}(\Omega )$*is the unique solution of the boundary value problem*
  (4.4) $$(-\Delta -\lambda )f=0,\;\tau_{D}f=\varphi .$$
(iv) *The values*$M(\lambda ):L^{2}(\partial \Omega )⊃H^{1}(\partial \Omega )\to L^{2}(\partial \Omega )$*of the Weyl function are Dirichlet-to-Neumann maps given by*
  $$M(\lambda )\varphi =-\tau_{N}f,\;\varphi \in H^{1}(\partial \Omega ),\;\lambda \in \rho (\Delta_{D}),$$
  *where*f=γ(λ)φ*is the unique solution of*(4.4)*. The operators*M(λ)*are bounded from*$H^{1}(\partial \Omega )$*to*$L^{2}(\partial \Omega )$*and if, in addition,*$\lambda \in \rho (\Delta_{N})$*then the Neumann-to-Dirichlet map*$M{\left(\lambda \right)}^{-1}$*is a compact operator in*$L^{2}(\partial \Omega )$*.*

Proof We check that $\left\{L^{2}(\partial \Omega ),\Gamma_{0},\Gamma_{1}\right\}$ is a quasi-boundary triple for $T\subset A^{⁎}$. From [33, Theorems 2.6 and 2.10, Lemmas 3.4 and 4.8] we obtain that the Dirichlet and Neumann Laplacian in (4.3) are both self-adjoint in $L^{2}(\Omega )$; for the $H^{3/2}$-regularity of the Dirichlet domain see also [48] or [49, Theorem B.2]. In particular, item (iii) in Definition 2.1 is valid and assertion (i) of the theorem holds.

The fact that ran *Γ* is dense in $L^{2}(\partial \Omega )\times L^{2}(\partial \Omega )$ will follow below when we verify assertion (ii) of the theorem. For the moment we note that item (ii) in Definition 2.1 holds.

The continuity of the trace maps $\tau_{D},\;\tau_{N}:H_{\Delta }^{3/2}(\Omega )\to L^{2}(\partial \Omega )$ and the fact that $C^{\infty }(\bar{\Omega })$ is dense in $H_{\Delta }^{3/2}(\Omega )$ (see [22, Lemme 3]) yield Green's identity

$${\left(Tf,g\right)}_{L^{2}(\Omega )}-{\left(f,Tg\right)}_{L^{2}(\Omega )}={\left(-\Delta f,g\right)}_{L^{2}(\Omega )}-{\left(f,-\Delta g\right)}_{L^{2}(\Omega )}={\left(-\tau_{N}f,\tau_{D}g\right)}_{L^{2}(\partial \Omega )}-{\left(\tau_{D}f,-\tau_{N}g\right)}_{L^{2}(\partial \Omega )}={\left(\Gamma_{1}f,\Gamma_{0}g\right)}_{L^{2}(\partial \Omega )}-{\left(\Gamma_{0}f,\Gamma_{1}g\right)}_{L^{2}(\partial \Omega )}$$

for all $f,\;g\in H_{\Delta }^{3/2}(\Omega )$, that is, condition (i) in Definition 2.1 holds.

Furthermore, as $C^{\infty }(\bar{\Omega })$ is dense in $H_{\Delta }^{0}(\Omega )=\mathrm{dom}\;A^{⁎}$ it follows that $\bar{T}=A^{⁎}=\Delta_{\max}$ holds. Therefore $\left\{L^{2}(\partial \Omega ),\Gamma_{0},\Gamma_{1}\right\}$ is a quasi-boundary triple for *T*. Hence we also obtain $T↾\ker\Gamma =A=\Delta_{\min}$ from the fact that ker⁡Γ=domA holds in every quasi-boundary triple.

Next we verify assertion (ii) (which also implies property (ii) in the definition of a quasi-boundary triple). Recall that $\mathrm{ran}\;\Gamma_{1}=L^{2}(\partial \Omega )$ by (4.1) and suppose that $h\;⊥\;G_{0}$. Choose $f\in \mathrm{dom}\;\Gamma_{1}$ such that $h=\Gamma_{1}f$. Then for all $g\in \ker\Gamma_{1}=\mathrm{dom}\;\Delta_{N}$ Green's identity yields

$$0={\left(h,\Gamma_{0}g\right)}_{L^{2}(\partial \Omega )}={\left(\Gamma_{1}f,\Gamma_{0}g\right)}_{L^{2}(\partial \Omega )}-{\left(\Gamma_{0}f,\Gamma_{1}g\right)}_{L^{2}(\partial \Omega )}={\left(Tf,g\right)}_{L^{2}(\Omega )}-{\left(f,\Delta_{N}g\right)}_{L^{2}(\Omega )}$$

and since $\Delta_{N}$ is selfadjoint by (i) we obtain $f\in \mathrm{dom}\;\Delta_{N}=\ker\Gamma_{1}$ and hence $h=\Gamma_{1}f=0$, that is, $G_{0}$ is dense in $L^{2}(\partial \Omega )$. The fact that $G_{1}$ is dense in $L^{2}(\partial \Omega )$ follows from [36, Lemma 6.3 and Corollary 6.5] since the subspace $\mathrm{ran}(\tau_{N}↾\{f\in H^{2}(\Omega ):\tau_{D}f=0\})$ of $G_{1}$ is dense in $L^{2}(\partial \Omega )$. This shows assertion (ii). Since $G_{0}\times G_{1}\subset \mathrm{ran}\;\Gamma$ also ran *Γ* is dense in $L^{2}(\partial \Omega )\times L^{2}(\partial \Omega )$ as noted above.

Most of the assertions in (iii) and (iv) are immediate consequences of the definition of the *γ*-field and the Weyl function corresponding to the quasi-boundary triple $\left\{L^{2}(\partial \Omega ),\Gamma_{0},\Gamma_{1}\right\}$. For the boundedness of M(λ) regarded as an operator from $H^{1}(\partial \Omega )$ into $L^{2}(\partial \Omega )$ and the compactness of $M{\left(\lambda \right)}^{-1}$ as an operator in $L^{2}(\partial \Omega )$ we refer to [33, Theorem 3.7 and Remark 3.8].  □

Let $\left\{L^{2}(\partial \Omega ),\Gamma_{0},\Gamma_{1}\right\}$ be the quasi-boundary triple for $T\subset A^{⁎}=\Delta_{\max}$ from Theorem 4.1 with Weyl function *M*. Equip the spaces $G_{0}$ and $G_{1}$ with the norms induced by

(4.5) (φ,ψ)G0:=(Σ−1/2φ,Σ−1/2ψ)L2(∂Ω),Σ=Im(−M(i)−1),(φ,ψ)G1:=(Λ−1/2φ,Λ−1/2ψ)L2(∂Ω),Λ=ImM(i)¯;

cf. Section 2.3. As an immediate consequence of Proposition 2.10 and Corollary 2.11, see also Definition 2.14, Lemma 2.15 and Lemma 2.16, we obtain a trace theorem for the Dirichlet and Neumann trace operator on the maximal domain of the Laplacian.

Corollary 4.2 *Let Ω be a bounded Lipschitz domain. Then the following statements hold.*

(i) *The Dirichlet trace operator*$\tau_{D}$*and Neumann trace operator*$\tau_{N}$*can be extended by continuity to surjective mappings*
  $${\overset{˜}{\tau }}_{D}:H_{\Delta }^{0}(\Omega )\to G_{1}^{\prime }\;\text{and}\;{\overset{˜}{\tau }}_{N}:H_{\Delta }^{0}(\Omega )\to G_{0}^{\prime }$$
  *such that*$\ker{\overset{˜}{\tau }}_{D}=\ker\tau_{D}=\mathrm{dom}\;\Delta_{D}$*and*$\ker{\overset{˜}{\tau }}_{N}=\ker\tau_{N}=\mathrm{dom}\;\Delta_{N}$*. In particular,*
  $$H_{0}^{2}(\Omega )=\left\{f\in H_{\Delta }^{0}(\Omega ):{\overset{˜}{\tau }}_{D}f={\overset{˜}{\tau }}_{N}f=0\right\}.$$
(ii) *For all*$\lambda \in \rho (\Delta_{D})$*the values of the γ-field γ from*Theorem 4.1*admit continuous extensions*
  $$\overset{˜}{\gamma }(\lambda ):G_{1}^{\prime }\to L^{2}(\partial \Omega ),\;\varphi \mapsto \overset{˜}{\gamma }(\lambda )\varphi =f,$$
  *where*$f\in L^{2}(\Omega )$*is the unique solution of*(4.4)*with*$\varphi \in G_{1}^{\prime }$*. In particular, the space*$G_{1}^{\prime }$*is maximal in the sense that whenever*$f\in L^{2}(\Omega )$*is a solution of the Dirichlet problem*(4.4)*then the boundary value φ belongs to*$G_{1}^{\prime }$*.*
(iii) *For all*$\lambda \in \rho (\Delta_{D})$*the values*M(λ)*of the Weyl function M from*Theorem 4.1*admit continuous extensions*
  $$\overset{˜}{M}(\lambda ):G_{1}^{\prime }\to G_{0}^{\prime },\;\varphi \mapsto \overset{˜}{M}(\lambda )\varphi =-{\overset{˜}{\tau }}_{N}f,\;\lambda \in \rho (\Delta_{D}),$$
  *where*$f=\overset{˜}{\gamma }(\lambda )\varphi$*is the unique solution of*(4.4)*with*$\varphi \in G_{1}^{\prime }$*.*

Applying Theorem 2.12 to the quasi-boundary triple $\left\{L^{2}(\partial \Omega ),\Gamma_{0},\Gamma_{1}\right\}$ from Theorem 4.1 we get a Lipschitz domain version of the ordinary boundary triple for the Laplacian as it appears already in the smooth case in [39], see also, e.g. [10], [12], [17], [53]. Recall that there exist isometric isomorphisms $\iota_{+}:G_{1}\to L^{2}(\partial \Omega )$, $\iota_{-}:G_{1}^{\prime }\to L^{2}(\partial \Omega )$ such that ${\left(\iota_{-}x^{\prime },\iota_{+}x\right)}_{L^{2}(\partial \Omega )}={\langle x^{\prime },x\rangle }_{G_{1}^{\prime }\times G_{1}}$; cf. (2.15).

Corollary 4.3 *Let*$\eta \in \rho (\Delta_{D})\cap R$*and let*${ϒ}_{0},{ϒ}_{1}:H_{\Delta }^{0}(\Omega )\to L^{2}(\partial \Omega )$*be given by*

$${ϒ}_{0}f:=\iota_{-}{\overset{˜}{\tau }}_{D}f,\;{ϒ}_{1}f:=-\iota_{+}\tau_{N}f_{D},\;f=f_{D}+f_{\eta }\in \mathrm{dom}\;\Delta_{D}∔N_{\eta }\left(A^{⁎}\right).$$

*Then*$\left\{L^{2}(\partial \Omega ),{ϒ}_{0},{ϒ}_{1}\right\}$*is an ordinary boundary triple for*$A^{⁎}=\Delta_{\max}$*with*$A^{⁎}↾\ker{ϒ}_{0}=\Delta_{D}$*and*

$$A^{⁎}↾\ker{ϒ}_{1}=\Delta_{\min}\overset{˙}{+}\left\{{\left(f_{\eta },\eta f_{\eta }\right)}^{⊤}:-\Delta f_{\eta }=\eta f_{\eta },\;f_{\eta }\in H_{\Delta }^{0}(\Omega )\right\}.$$

In the present setting Theorem 3.7 can be applied to the quasi-boundary triple from Theorem 4.1. This yields a description of all self-adjoint extensions $\Delta_{\vartheta }\subset \Delta_{\max}$ of the minimal Laplacian $\Delta_{\min}$ in $L^{2}(\Omega )$ on bounded Lipschitz domains.

Corollary 4.4 *Let Ω be a bounded Lipschitz domain,* $G_{0}$ *,* $G_{1}$ *be as in* Theorem 4.1 *,* $\eta \in R\cap \rho (\Delta_{D})\cap \rho (\Delta_{N})$ *and* $\overset{˜}{M}(\eta ):G_{1}^{\prime }\to G_{0}^{\prime }$ *be the extended Dirichlet-to-Neumann map. Then the mapping*

$$\Theta \mapsto \Delta_{\vartheta }=\Delta_{\max}↾\left\{f\in H_{\Delta }^{0}(\Omega ):\vartheta {\overset{˜}{\tau }}_{D}f+{\overset{˜}{\tau }}_{N}f=0\right\},\;\vartheta =\iota_{+}^{-1}\Theta \iota_{-}+\overset{˜}{M}(\eta ),$$

*establishes a bijective correspondence between all closed (symmetric, self-adjoint, (maximal) dissipative, (maximal) accumulative) linear relations Θ in* $L^{2}(\partial \Omega )$ *and all closed (symmetric, self-adjoint, (maximal) dissipative, (maximal) accumulative, respectively) extensions* $\Delta_{\vartheta }\subset A^{⁎}=\Delta_{\max}$ *of* $A=\Delta_{\min}$ *in* $L^{2}(\Omega )$ *. Moreover, the following regularity result holds: If* $\Delta_{s}$ *is an extension of T in* (4.2) *such that* $\Delta_{s}\subset A^{⁎}=\Delta_{\max}$ *then*

(4.6) $$\mathrm{dom}\;\Theta \subset \mathrm{ran}(\iota_{-}{\overset{˜}{\tau }}_{D}↾\mathrm{dom}\;\Delta_{s})\;\text{implies}\mathrm{dom}\;\Delta_{\vartheta }\subset \mathrm{dom}\;\Delta_{s}.$$

We note that the abstract propositions from Section 3.2 can be applied to the quasi-boundary triple $\left\{L^{2}(\partial \Omega ),\Gamma_{0},\Gamma_{1}\right\}$, see also Section 4.3. We leave the formulations to the reader and state only a version of Kreĭn's formula as in Corollary 3.14.

Corollary 4.5 *Let Ω be a bounded Lipschitz domain,*$\overset{˜}{\gamma }(\lambda ):G_{1}^{\prime }\to L^{2}(\Omega )$*and*$\overset{˜}{M}(\lambda ):G_{1}^{\prime }\to G_{0}^{\prime }$*be the extended γ-field and Dirichlet-to-Neumann map from*Corollary 4.2*. Let*$\vartheta \subset G_{1}^{\prime }\times G_{0}^{\prime }$*be a linear relation in*$\mathrm{ran}({\overset{˜}{\tau }}_{D},-{\overset{˜}{\tau }}_{N})$*such that*

$$\Delta_{\vartheta }=\Delta_{\max}↾\left\{f\in H_{\Delta }^{0}(\Omega ):\vartheta {\overset{˜}{\tau }}_{D}f+{\overset{˜}{\tau }}_{N}f=0\right\}$$

*is closed in*$L^{2}(\Omega )$*. Then for all*$\lambda \in \rho (\Delta_{D})$*the following assertions* (i)–(iv) *hold.*

(i) $\lambda \in \sigma_{p}(\Delta_{\vartheta })$*if and only if*$0\in \sigma_{p}(\iota_{+}(\vartheta -\overset{˜}{M}(\lambda ))\iota_{-}^{-1})$*, in this case*
  $$\ker(\Delta_{\vartheta }-\lambda )=\overset{˜}{\gamma }(\lambda )\ker\left(\vartheta -\overset{˜}{M}(\lambda )\right),$$
(ii) $\lambda \in \sigma_{c}(\Delta_{\vartheta })$*if and only if*$0\in \sigma_{c}(\iota_{+}(\vartheta -\overset{˜}{M}(\lambda ))\iota_{-}^{-1})$*,*
(iii) $\lambda \in \sigma_{r}(\Delta_{\vartheta })$*if and only if*$0\in \sigma_{r}(\iota_{+}(\vartheta -\overset{˜}{M}(\lambda ))\iota_{-}^{-1})$*,*
(iv) $\lambda \in \rho (\Delta_{\vartheta })$*if and only if*$0\in \rho (\iota_{+}(\vartheta -\overset{˜}{M}(\lambda ))\iota_{-}^{-1})$*and*
  $${\left(\Delta_{\vartheta }-\lambda \right)}^{-1}={\left(\Delta_{D}-\lambda \right)}^{-1}+\overset{˜}{\gamma }(\lambda ){\left(\vartheta -\overset{˜}{M}(\lambda )\right)}^{-1}\overset{˜}{\gamma }{\left(\overset{¯}{\lambda }\right)}^{\prime }$$
  *holds for all*$\lambda \in \rho (\Delta_{\vartheta })\cap \rho (\Delta_{D})$*.*

In the following we slightly improve Lemma 3.2 by using the fact that $\ker\tau_{N}=\ker{\overset{˜}{\tau }}_{N}=\mathrm{dom}\;\Delta_{N}$.

Lemma 4.6 *Let Ω be a bounded Lipschitz domain and let ϑ be a linear relation in* $L^{2}(\partial \Omega )$ *. Then*

$$\Delta_{\vartheta }:=\Delta_{\max}↾\left\{f\in H_{\Delta }^{0}(\Omega ):\vartheta {\overset{˜}{\tau }}_{D}f+{\overset{˜}{\tau }}_{N}f=0\right\}$$

*has regularity* $\mathrm{dom}\;\Delta_{\vartheta }\subset H_{\Delta }^{3/2}(\Omega )$ *. Moreover,* $\Delta_{\vartheta }$ *is symmetric in* $L^{2}(\Omega )$ *if and only if ϑ is symmetric* $L^{2}(\partial \Omega )$ *.*

Proof For $f\in \mathrm{dom}\;\Delta_{\vartheta }$ we have $\vartheta {\overset{˜}{\tau }}_{D}f=-{\overset{˜}{\tau }}_{N}f\in L^{2}(\partial \Omega )$ as *ϑ* is assumed to be a linear relation in $L^{2}(\partial \Omega )$. By (4.1) there exists $g\in H_{\Delta }^{3/2}(\Omega )$ such that $\tau_{N}g={\overset{˜}{\tau }}_{N}f$ and hence

$$f-g\in \ker{\overset{˜}{\tau }}_{N}=\ker\tau_{N}=\mathrm{dom}\;\Delta_{N}\subset H_{\Delta }^{3/2}(\Omega ).$$

Therefore $f=(f-g)+g\in H_{\Delta }^{3/2}(\Omega )$ and $\mathrm{dom}\;\Delta_{\vartheta }\subset H_{\Delta }^{3/2}(\Omega )$ holds. In particular, we have

(4.7) $$\Delta_{\vartheta }=\Delta_{\max}↾\left\{f\in H_{\Delta }^{3/2}(\Omega ):\vartheta \Gamma_{0}f-\Gamma_{1}f=0\right\},$$

where $\left\{L^{2}(\partial \Omega ),\Gamma_{0},\Gamma_{1}\right\}$ is the quasi-boundary triple from Theorem 4.1. Then by Lemma 3.2 $\Delta_{\vartheta }$ is symmetric in $L^{2}(\Omega )$ if and only if *ϑ* is symmetric $L^{2}(\partial \Omega )$.  □

The next theorem is a slightly improved Lipschitz domain version of [11, Theorem 4.8], see also [12, Theorem 6.21].

Theorem 4.7 *Let Ω be a bounded Lipschitz domain and let ϑ be a bounded self-adjoint operator in* $L^{2}(\partial \Omega )$ *. Then*

(4.8) $$\Delta_{\vartheta }:=\Delta_{\max}↾\left\{f\in H_{\Delta }^{0}(\Omega ):\vartheta {\overset{˜}{\tau }}_{D}f+{\overset{˜}{\tau }}_{N}f=0\right\}$$

*is a self-adjoint operator in* $L^{2}(\Omega )$ *with compact resolvent, semibounded from below and regularity* $\mathrm{dom}\;\Delta_{\vartheta }\subset H_{\Delta }^{3/2}(\Omega )$ *.*

Proof It follows from Lemma 4.6 that $\mathrm{dom}\;\Delta_{\vartheta }\subset H_{\Delta }^{3/2}(\Omega )$ holds and hence $\Delta_{\vartheta }$ is given by (4.7), where $\left\{L^{2}(\partial \Omega ),\Gamma_{0},\Gamma_{1}\right\}$ is the quasi-boundary triple for $T\subset \Delta_{\max}$ from Theorem 4.1 with Weyl function *M*. According to Theorem 4.1(iv) the Neumann-to-Dirichlet maps $M{\left(\lambda \right)}^{-1}$, $\lambda \in \rho (\Delta_{D})\cap \rho (\Delta_{N})$, are compact operators in $L^{2}(\partial \Omega )$, and hence [12, Theorem 6.21] implies that $\Delta_{\vartheta }$ is a self-adjoint operator in $L^{2}(\Omega )$. The compactness of the resolvent of $\Delta_{\vartheta }$ follows from [11, Theorem 4.8] applied to the quasi-boundary triple $\left\{L^{2}(\partial \Omega ),\Gamma_{1},-\Gamma_{0}\right\}$ and the parameter $\Theta =-\vartheta^{-1}$.

It remains to show that $\Delta_{\vartheta }$ is semibounded from below. If ϑ=0 this is obviously true. Suppose ϑ≠0, let 0<ε≤1/‖ϑ‖ and choose $c_{\varepsilon }>0$ such that

$${\left\|\tau_{D}g\right\|}_{L^{2}(\partial \Omega )}^{2}\leq \varepsilon {\left\|\nabla g\right\|}_{L^{2}{\left(\Omega \right)}^{n}}^{2}+c_{\varepsilon }{\left\|g\right\|}_{L^{2}(\Omega )}^{2},\;g\in H^{1}(\Omega );$$

see, e.g. [35, Lemma 4.2]. For $f\in \mathrm{dom}\;\Delta_{\vartheta }$ Green's identity together with $-\tau_{N}f=\vartheta \tau_{D}f$ (see (4.8)) implies

$${\left(\Delta_{\vartheta }f,\;f\right)}_{L^{2}(\Omega )}={\left\|\nabla f\right\|}_{L^{2}{\left(\Omega \right)}^{n}}^{2}+{\left(\vartheta \tau_{D}f,\;\tau_{D}f\right)}_{L^{2}(\partial \Omega )}\geq {\left\|\nabla f\right\|}_{L^{2}{\left(\Omega \right)}^{n}}^{2}-\|\vartheta \|\;{\left\|\tau_{D}f\right\|}_{L^{2}(\partial \Omega )}^{2}\geq {\left\|\nabla f\right\|}_{L^{2}{\left(\Omega \right)}^{n}}^{2}-\varepsilon \|\vartheta \|{\left\|\nabla f\right\|}_{L^{2}{\left(\Omega \right)}^{n}}^{2}-c_{\varepsilon }\|\vartheta \|{\left\|f\right\|}_{L^{2}(\Omega )}^{2}\geq -c_{\varepsilon }\|\vartheta \|\;{\left\|f\right\|}_{L^{2}(\partial \Omega )}^{2}.$$

 □

In the next corollary we formulate a version of Theorem 4.7 for Robin boundary conditions.

Corollary 4.8 *Let Ω be a bounded Lipschitz domain and let*$\alpha \in L^{\infty }(\partial \Omega )$*be a real function on* ∂*Ω. Then*

(4.9) $$\Delta_{\alpha }:=\Delta_{\max}↾\left\{f\in H_{\Delta }^{0}(\Omega ):\alpha \cdot {\overset{˜}{\tau }}_{D}f+{\overset{˜}{\tau }}_{N}f=0\right\}$$

*is self-adjoint operator in* $L^{2}(\Omega )$ *with compact resolvent, semibounded from below and regularity* $\mathrm{dom}\;\Delta_{\alpha }\subset H_{\Delta }^{3/2}(\Omega )$*. In* (4.9) *the multiplication with α is understood as an operator in* $L^{2}(\partial \Omega )$*.*

In the end of this subsection we establish the link to [36] and briefly discuss two more special cases of bounded Lipschitz domains: so-called quasi-convex domains in Theorem 4.9 and $C^{1,r}$-domains with $r\in (\frac{1}{2},1]$ in Theorem 4.10.

For the definition of quasi-convex domains we refer to [36, Definition 8.9]. We mention that all convex domains, all almost-convex domains, all domains that satisfy a local exterior ball condition, as well as all $C^{1,r}$-domains with $r\in (\frac{1}{2},1]$ are quasi-convex, for more details on almost-convex domains see [58]. The key feature of a quasi-convex domain is that the Dirichlet- and Neumann Laplacian have $H^{2}$-regularity,

(4.10) $$\mathrm{dom}\;\Delta_{D}\subset H^{2}(\Omega ),\;\mathrm{dom}\;\Delta_{N}\subset H^{2}(\Omega ).$$

For the next theorem we recall the definition of the tangential gradient operator

$$\nabla_{\tan}:H^{1}(\partial \Omega )\to L^{2}{\left(\partial \Omega \right)}^{n},\;\nabla_{\tan}f:={\left(\sum_{j=1}^{n}n_{j}\partial_{\tau_{j,k}}f\right)}_{k=1,\ldots ,n}^{⊤}$$

from [36, (6.1)]. Here $\partial_{\tau_{j,k}}:=n_{j}\partial_{k}-n_{k}\partial_{j}$, j,k∈{1,…,n}, are the first-order tangential differential operators acting continuously from $H^{1}(\partial \Omega )$ to $L^{2}(\partial \Omega )$.

Theorem 4.9 *Let Ω be a quasi-convex domain. Then the following statements hold.*

(i) *The spaces* $G_{0}$ *and* $G_{1}$ *in Theorem* 4.1 *are given by*
  $$G_{0}=\left\{\varphi \in H^{1}(\partial \Omega ):\nabla_{\tan}\varphi \in H^{1/2}{\left(\partial \Omega \right)}^{n}\right\},$$
  $$G_{1}=\left\{\psi \in L^{2}(\partial \Omega ):\psi \;n\in H^{1/2}{\left(\partial \Omega \right)}^{n}\right\},$$
  *and for the norms* ${\left\|\cdot \right\|}_{G_{0}}$ *and* ${\left\|\cdot \right\|}_{G_{1}}$ *induced by the inner products in* (4.5) *the following equivalences hold:*
  $${\left\|\varphi \right\|}_{G_{0}}∼{\left\|\varphi \right\|}_{L^{2}(\partial \Omega )}+{\left\|\nabla_{\tan}\varphi \right\|}_{H^{1/2}{\left(\partial \Omega \right)}^{n}},\;\varphi \in G_{0},$$
  $${\left\|\psi \right\|}_{G_{1}}∼{\left\|\psi \;n\right\|}_{H^{1/2}{\left(\partial \Omega \right)}^{n}},\;\psi \in G_{1}.$$
(ii) *The Dirichlet trace operator* $\tau_{D}$ *and Neumann trace operator* $\tau_{N}$ *admit continuous, surjective extensions to*
  $${\overset{˜}{\tau }}_{D}:H_{\Delta }^{0}(\Omega )\to {\left(\left\{\psi \in L^{2}(\partial \Omega ):\psi \;n\in H^{1/2}{\left(\partial \Omega \right)}^{n}\right\}\right)}^{\prime },$$
  $${\overset{˜}{\tau }}_{N}:H_{\Delta }^{0}(\Omega )\to {\left(\left\{\varphi \in H^{1}(\partial \Omega ):\nabla_{\tan}\varphi \in H^{1/2}{\left(\partial \Omega \right)}^{n}\right\}\right)}^{\prime }.$$

Proof Let *Ω* be a bounded Lipschitz domain. It follows from the considerations in [55, Section 7] (see also [36, Theorem 6.1]) that the trace operator $f\mapsto {\left(\tau_{D}f,\tau_{N}f\right)}^{⊤}$, $f\in C^{\infty }(\bar{\Omega })$, admits a continuous extension to a mapping from $H^{2}(\Omega )$ onto the space of all ${\left(\varphi ,\psi \right)}^{⊤}\in H^{1}(\partial \Omega )\times L^{2}(\partial \Omega )$ such that $\nabla_{\tan}\varphi +\psi \;n\in H^{1/2}{\left(\partial \Omega \right)}^{n}$; here $H^{1}(\partial \Omega )\times L^{2}(\partial \Omega )$ is equipped with the norm

$${\left\|\varphi \right\|}_{H^{1}(\partial \Omega )}+{\left\|\psi \right\|}_{L^{2}(\partial \Omega )}+{\left\|\nabla_{\tan}\varphi +\psi n\right\|}_{H^{1/2}{\left(\partial \Omega \right)}^{n}}.$$

The kernel of this extension of ${\left(\tau_{D},\;\tau_{N}\right)}^{⊤}$ is $H_{0}^{2}(\Omega )$. This implies that the Dirichlet trace operator $\tau_{D}$ admits a continuous extension to a surjective mapping from

$$\left\{f\in H^{2}(\Omega ):\tau_{N}f=0\right\}\;\text{onto}\;\left\{\varphi \in H^{1}(\partial \Omega ):\nabla_{\tan}\varphi \in H^{1/2}{\left(\partial \Omega \right)}^{n}\right\}$$

and the Neumann trace operator $\tau_{N}$ admits a continuous extension to a surjective mapping from

$$\left\{f\in H^{2}(\Omega ):\tau_{D}f=0\right\}\;\text{onto}\;\left\{\psi \in L^{2}(\partial \Omega ):\psi \;n\in H^{1/2}{\left(\partial \Omega \right)}^{n}\right\};$$

cf. [36, Lemma 6.3 and Lemma 6.9]. Now let *Ω* be a quasi-convex domain. Then according to [36, Lemma 8.11] the regularity properties (4.10) hold, and since $G_{0}$, $G_{1}$ are Hilbert spaces, which are dense in $L^{2}(\partial \Omega )$ the assertions follow from Proposition 2.10 and Corollary 2.11.  □

We note that Theorem 4.9 is essentially the same as [36, Theorems 6.4 and 6.10], and also implies [36, Corollaries 10.3 and 10.7]. Theorem 4.9 together with Corollary 4.4 yields results of similar form as in [36, Sections 14 and 15]; the Kreĭn type resolvent formulas in [36, Section 16] can also be viewed as consequences of Corollary 4.5.

In the next theorem we treat the case of $C^{1,r}$-domains with $r\in (\frac{1}{2},1]$. In a similar manner as above this theorem combined with the earlier abstract results leads to various results on self-adjoint realizations or Kreĭn type resolvent formulas in the flavor of [36].

Theorem 4.10 *Let Ω be a* $C^{1,r}$ *-domain with* $r\in (\frac{1}{2},1]$ *. Then the following statements hold.*

(i) *The spaces* $G_{0}$ *and* $G_{1}$ *in* Theorem 4.1 *are given by*
  $$G_{0}=H^{3/2}(\partial \Omega )\;\text{and}\;G_{1}=H^{1/2}(\partial \Omega )$$
  *and the norms induced by the inner products in* (4.5) *are equivalent to the usual norms in* $H^{3/2}(\partial \Omega )$ *and* $H^{1/2}(\partial \Omega )$ *, respectively.*
(ii) *The Dirichlet trace operator* $\tau_{D}$ *and Neumann trace operator* $\tau_{N}$ *admit continuous, surjective extensions to*
  $$\tau_{D}:H_{\Delta }^{0}(\Omega )\to H^{-1/2}(\partial \Omega )\;\text{and}\;\tau_{N}:H_{\Delta }^{0}(\Omega )\to H^{-3/2}(\partial \Omega ).$$

*Moreover, the following regularity result holds: For* $0\leq s\leq \frac{3}{2}$

(4.11) $$\mathrm{dom}\;\Theta \subset H^{s}(\partial \Omega )\;\text{implies}\;\mathrm{dom}\;\Delta_{\Theta }\subset H_{\Delta }^{s}(\Omega ).$$

Proof Note that (4.10) holds for the Dirichlet and Neumann Laplacian and that the trace operator $f\mapsto {\left(\tau_{D},\tau_{N}\right)}^{⊤}$, $f\in C^{\infty }(\bar{\Omega })$, admits a continuous extension to a mapping from $H^{2}(\Omega )$ onto $H^{3/2}(\partial \Omega )\times H^{1/2}(\partial \Omega )$, see, e.g. [54, Theorem 2]. Hence statements (i) and (ii) follow from Proposition 2.10 and Corollary 2.11. It remains to verify the regularity result (4.11). Let $\Delta_{s}:=\Delta_{\max}↾H_{\Delta }^{s}(\Omega )$ with $0\leq s\leq \frac{3}{2}$, so that *T* in (4.2) is contained in $\Delta_{s}\subset A^{⁎}=\Delta_{\max}$. Since $\mathrm{ran}({\overset{˜}{\tau }}_{D}↾\mathrm{dom}\;\Delta_{s})=H^{s-1/2}(\partial \Omega )$ and $\iota_{-}$ is an isometry from $H^{s-1/2}(\partial \Omega )$ onto $H^{s}(\partial \Omega )$ the assertion (4.11) follows from the abstract regularity result (4.6) in Corollary 4.4.  □

### 4.2 Elliptic differential operators of order 2*m* on bounded smooth domains

In this subsection we briefly illustrate some of the abstract results from Section 2 and Section 3 for elliptic differential operators of order 2*m* on a bounded smooth domain. The description of the selfadjoint realizations in this case can already be found in Grubb [39], other extension properties obtained below can be found in the monograph of Lions and Magenes [52]. We also refer the reader to the classical contributions [8], [9], [16], [32], [39], [52], [68] for more details on the notation and references, and to, e.g. [17], [45], [53] for some recent connected publications.

Let $\Omega \subset R^{n}$, n≥2, be a bounded domain with $C^{\infty }$-boundary ∂*Ω*. Let *A* and *T* be the realizations of the 2*m*-th order, properly elliptic, formally self-adjoint differential expression

$$L:=\sum_{|\alpha |,|\beta |\leq m}{\left(-1\right)}^{\left|\alpha \right|}\partial^{\alpha }a_{\alpha \beta }\partial^{\beta },\;a_{\alpha \beta }\in C^{\infty }(\bar{\Omega }),$$

on $H_{0}^{2m}(\Omega )$ and $H^{2m}(\Omega )$, respectively; cf. [52, Chapter 2.1] for more details. As in Section 4.1 we define the Hilbert spaces

(4.12) $$H_{L}^{s}(\Omega ):=\left\{f\in H^{s}(\Omega ):Lf\in L^{2}(\Omega )\right\},\;s\geq 0,$$

with norms induced by the inner products given by

(4.13) $${\left(f,g\right)}_{H_{L}^{s}(\Omega )}:={\left(f,g\right)}_{H^{s}(\Omega )}+{\left(Lf,Lg\right)}_{L^{2}(\Omega )},\;f,g\in H_{L}^{s}(\Omega ).$$

We note that $H_{L}^{s}(\Omega )=H^{s}(\Omega )$ with equivalent norms if s≥2m and that $C^{\infty }(\bar{\Omega })$ is dense in $H_{L}^{s}(\Omega )$ for s≥0. The minimal and the maximal realization of the differential expression L are given by

$$L_{\min}:=A=L↾H_{0}^{2m}(\Omega )\;\text{and}\;L_{\max}:=A^{⁎}=L↾H_{L}^{0}(\Omega ),$$

respectively. We mention that *A* is a closed, densely defined, symmetric operator in $L^{2}(\Omega )$ with equal infinite deficiency indices.

In the next theorem a quasi-boundary triple for the elliptic differential operator *T* is defined. Here we make use of normal systems $D={\left\{D_{j}\right\}}_{j=0}^{m-1}$ and $N={\left\{N_{j}\right\}}_{j=0}^{m-1}$ of boundary differential operators,

(4.14) $$D_{j}f:=\sum_{|\beta |\leq m_{j}}b_{j\beta }\;\partial^{\beta }f{↾}_{\partial \Omega },\;f\in H^{2m}(\Omega ),\;m_{j}\leq 2m-1,$$

(4.15) $$N_{j}f:=\sum_{|\beta |\leq \mu_{j}}c_{j\beta }\;\partial^{\beta }f{↾}_{\partial \Omega },\;f\in H^{2m}(\Omega ),\;\mu_{j}\leq 2m-1,$$

with $C^{\infty }$ coefficients $b_{j\beta },c_{j\beta }$ on ∂*Ω* and which cover L on ∂*Ω*; cf. [52, Chapter 2.1].

Theorem 4.11 *Let D be a normal system of boundary differential operators as in* (4.14) *. Then there exists a normal system of boundary differential operators N of the form* (4.15) *of order* $\mu_{j}=2m-m_{j}-1$ *, such that* $\left\{L^{2}{\left(\partial \Omega \right)}^{m},\Gamma_{0},\Gamma_{1}\right\}$ *,*

$$\Gamma_{0},\Gamma_{1}:H^{2m}(\Omega )\to L^{2}{\left(\partial \Omega \right)}^{m},\;\Gamma_{0}f:=Df,\;\Gamma_{1}f:=Nf,$$

*is a quasi-boundary triple for* $T\subset A^{⁎}$ *. The minimal realization* $A=L_{\min}$ *coincides with* T↾ker⁡Γ *and the following statements hold.*

(i) *The Dirichlet realization* $L_{D}$ *and Neumann realization* $L_{N}$ *correspond to* $\ker\Gamma_{0}$ *and* $\ker\Gamma_{1}$ *,*
  $$L_{D}:=T↾\ker\Gamma_{0}=L_{\max}↾\left\{f\in H^{2m}(\Omega ):Df=0\right\},$$
  $$L_{N}:=T↾\ker\Gamma_{1}=L_{\max}↾\left\{f\in H^{2m}(\Omega ):Nf=0\right\},$$
  *respectively, and* $L_{D}$ *is self-adjoint in* $L^{2}(\Omega )$ *.*
(ii) *The spaces*
  (4.16) $$G_{0}:=\mathrm{ran}(\Gamma_{0}↾\ker\Gamma_{1})=\prod_{j=0}^{m-1}H^{2m-m_{j}-1/2}(\partial \Omega ),$$
  $$G_{1}:=\mathrm{ran}(\Gamma_{1}↾\ker\Gamma_{0})=\prod_{j=0}^{m-1}H^{m_{j}+1/2}(\partial \Omega ),$$
  *are dense in* $L^{2}{\left(\partial \Omega \right)}^{m}$ *.*
(iii) *The values* $\gamma (\lambda ):L^{2}{\left(\partial \Omega \right)}^{m}⊃\prod_{j=0}^{m-1}H^{2m-m_{j}-1/2}(\partial \Omega )\to L^{2}(\Omega )$ *of the γ-field are given by*
  $$\gamma (\lambda )\varphi =f,\;\varphi \in \prod_{j=0}^{m-1}H^{2m-m_{j}-1/2}(\partial \Omega ),\;\lambda \in \rho (L_{D}),$$
  *where* $f\in L^{2}(\Omega )$ *is the unique solution of the boundary value problem*
  (4.17) (L−λ)f=0,Df=φ.
(iv) *The values* $M(\lambda ):L^{2}{\left(\partial \Omega \right)}^{m}⊃\prod_{j=0}^{m-1}H^{2m-m_{j}-1/2}(\partial \Omega )\to L^{2}{\left(\partial \Omega \right)}^{m}$ *of the Weyl function are given by*
  $$M(\lambda )\varphi =Nf,\;\varphi \in \prod_{j=0}^{m-1}H^{2m-m_{j}-1/2}(\partial \Omega ),\;\lambda \in \rho (L_{D}),$$
  *where* f=γ(λ)φ *is the unique solution of* (4.17) *.*

Proof First we remark that $C^{\infty }(\bar{\Omega })$, and hence $H^{2m}(\Omega )$, is dense in $H_{L}^{0}(\Omega )$. This implies $\bar{T}=A^{⁎}$. According to [52, Chapter 2.1] for a given normal system *D* of boundary differential operators as in (4.14) there exists a system a normal system *N* of boundary differential operators of the form (4.15) of order $\mu_{j}=2m-m_{j}-1$ such that {D,N} is a Dirichlet system of order 2m, which acts as a mapping from $H^{2m}(\Omega )$ onto

(4.18) $$\prod_{j=0}^{m-1}H^{2m-m_{j}-1/2}(\partial \Omega )\times \prod_{j=0}^{m-1}H^{m_{j}+1/2}(\partial \Omega )↪L^{2}{\left(\partial \Omega \right)}^{2m}.$$

The kernel of this map is $H_{0}^{2m}(\Omega )$ and Green's formula

$${\left(Lf,g\right)}_{L^{2}(\Omega )}-{\left(f,Lg\right)}_{L^{2}(\Omega )}={\left(Nf,Dg\right)}_{L^{2}{\left(\partial \Omega \right)}^{m}}-{\left(Df,Ng\right)}_{L^{2}{\left(\partial \Omega \right)}^{m}}$$

holds for all $f,g\in H^{2m}(\Omega )$; cf. [52, Theorem 2.2.1]. From (4.18) we conclude that (4.16) holds and the spaces $G_{0}$ and $G_{1}$ are dense in $L^{2}{\left(\partial \Omega \right)}^{m}$. This also implies that ran *Γ* is dense in $L^{2}{\left(\partial \Omega \right)}^{m}\times L^{2}{\left(\partial \Omega \right)}^{m}$. Moreover $A_{0}:=T↾\ker\Gamma_{0}=L_{D}$ is self-adjoint in $L^{2}(\Omega )$ by [52, Theorem 2.8.4]. Hence $\left\{L^{2}{\left(\partial \Omega \right)}^{m},\Gamma_{0},\Gamma_{1}\right\}$ is a quasi-boundary triple for $T\subset A^{⁎}$ with $T↾\ker\Gamma =L_{\min}=A$. The remaining statements follow from the definition of the *γ*-field and the Weyl function.  □

The next two corollaries show that the abstract theory from Section 2.3 implies some fundamental extension results due to Lions and Magenes. The proofs immediately follow from Proposition 2.10, Corollary 2.11 and standard interpolation theory of Sobolev spaces, see also Lemma 2.15 and Lemma 2.16.

Corollary 4.12 *Let*$\left\{L^{2}{\left(\partial \Omega \right)}^{m},\Gamma_{0},\Gamma_{1}\right\}$*be the quasi-boundary triple for*$T\subset A^{⁎}$*from*Theorem 4.11*with Weyl function M. Then the following statements hold.*

(i) *The mapping*$\Gamma_{0}=D$*admits a continuous extension to a surjective mapping*
  (4.19) $$\overset{˜}{D}:H_{L}^{0}(\Omega )\to \prod_{j=0}^{m-1}H^{-m_{j}-1/2}(\partial \Omega )$$
  *such that*$\ker\overset{˜}{D}=\ker D=\mathrm{dom}\;L_{D}$*.*
(ii) *The norm*
  ‖Λ−1/2f‖L2(∂Ω)m,Λ:=ImM(i)¯,f∈∏j=0m−1Hmj+1/2(∂Ω),
  *defines an equivalent norm on*$\prod_{j=0}^{m-1}H^{m_{j}+1/2}(\partial \Omega )$*.*

In the next corollary we assume, in addition, that $L_{N}=T↾\ker\Gamma_{1}$ is self-adjoint.

Corollary 4.13 *Let* $\left\{L^{2}{\left(\partial \Omega \right)}^{m},\Gamma_{0},\Gamma_{1}\right\}$ *be the quasi-boundary triple for* $T\subset A^{⁎}$ *from* Theorem 4.11 *with γ-field γ and Weyl function M. Assume that the realization* $L_{N}$ *of* L *is self-adjoint in* $L^{2}(\Omega )$ *. Then the following statements hold.*

(i) *The mapping* $\Gamma_{1}=N$ *admits a continuous extension to a surjective mapping*
  (4.20) $$\overset{˜}{N}:H_{L}^{0}(\Omega )\to \prod_{j=0}^{m-1}H^{-2m+m_{j}+1/2}(\partial \Omega )$$
  *such that* $\ker\overset{˜}{N}=\ker N=\mathrm{dom}\;L_{N}$ *.*
(ii) *The norm*
  ‖Σ−1/2f‖L2(∂Ω)m,Σ:=Im(−M(i)−1)¯,f∈∏j=0m−1H2m−mj−1/2(∂Ω),
  *defines an equivalent norm on* $\prod_{j=0}^{m-1}H^{2m-m_{j}-1/2}(\partial \Omega )$ *.*
(iii) *The values of the γ-field γ and the Weyl function M admit continuous extensions*
  $$\overset{˜}{\gamma }(\lambda ):\prod_{j=0}^{m-1}H^{-m_{j}-1/2}(\partial \Omega )\to L^{2}(\Omega ),$$
  $$\overset{˜}{M}(\lambda ):\prod_{j=0}^{m-1}H^{-m_{j}-1/2}(\partial \Omega )\to \prod_{j=0}^{m-1}H^{-2m+m_{j}+1/2}(\partial \Omega ),$$
  *for all* $\lambda \in \rho (L_{D})$ *.*
(iv) *The restrictions*
  (4.21) $$\overset{˜}{D}↾H_{L}^{s}(\Omega ):H_{L}^{s}(\Omega )\to \prod_{j=0}^{m-1}H^{s-m_{j}-1/2}(\partial \Omega ),$$
  $$\overset{˜}{N}↾H_{L}^{s}(\Omega ):H_{L}^{s}(\Omega )\to \prod_{j=0}^{m-1}H^{s-2m+m_{j}+1/2}(\partial \Omega ),$$
  *are continuous and surjective for all* s∈[0,2m] *.*

Corollary 4.12 and Corollary 4.13 imply that the maximal possible domain for a quasi-boundary triple with boundary mappings $\overset{˜}{D}$ and $\overset{˜}{N}$ is given by the space $H_{L}^{2m-1/2}(\Omega )$, see also [9].

Proposition 4.14 *Let*s∈[0,2m]*,*$T_{s}:=L_{\max}↾H_{L}^{s}(\Omega )$*, assume that*$L_{N}$*is self-adjoint and let*

$$\Gamma_{0}^{s}:H_{L}^{s}(\Omega )\to \prod_{j=0}^{m-1}H^{s-m_{j}-1/2}(\partial \Omega ),\;\Gamma_{0}^{s}f:=\overset{˜}{D}f,$$

$$\Gamma_{1}^{s}:H_{L}^{s}(\Omega )\to \prod_{j=0}^{m-1}H^{s-2m+m_{j}+1/2}(\partial \Omega ),\;\Gamma_{1}^{s}f:=\overset{˜}{N}f.$$

*Then the spaces*

$$G_{0}=\mathrm{ran}\;\left(\Gamma_{0}^{s}↾\ker\Gamma_{1}^{s}\right)=\prod_{j=0}^{m-1}H^{2m-m_{j}-1/2}(\partial \Omega ),$$

$$G_{1}=\mathrm{ran}\;\left(\Gamma_{1}^{s}↾\ker\Gamma_{0}^{s}\right)=\prod_{j=0}^{m-1}H^{m_{j}+1/2}(\partial \Omega )$$

*are dense in*$L^{2}(\partial \Omega )$*and do not depend on s. Moreover, if*$s\in [2m-\frac{1}{2},2m]$*then*$\mathrm{ran}\;\Gamma_{0}^{s}\subset L^{2}{\left(\partial \Omega \right)}^{m}$*,*$\mathrm{ran}\;\Gamma_{1}^{s}\subset L^{2}{\left(\partial \Omega \right)}^{m}$*, and*$\left\{L^{2}{\left(\partial \Omega \right)}^{m},\Gamma_{0}^{s},\Gamma_{1}^{s}\right\}$*is a quasi-boundary triple for*$T_{s}\subset A^{⁎}=L_{\max}$*.*

By applying Theorem 2.12 to the quasi-boundary triple $\left\{L^{2}{\left(\partial \Omega \right)}^{m},\Gamma_{0},\Gamma_{1}\right\}$ from Theorem 4.11 one obtains an ordinary boundary triple which appears implicitly already in [39], see also [17], [41] and [53, Propositions 3.5, 5.1]. The details of the formulation are left to the reader. As an example of the consequences of the abstract results from Section 2 and Section 3 we state only a version of Kreĭn's formula for the case of 2*m*-th order elliptic differential operators. We leave it to the reader to formulate the other corollaries from the general results, e.g. the description of the closed (symmetric, self-adjoint, (maximal) dissipative, (maximal) accumulative, respectively) extensions $L_{\vartheta }\subset L_{\max}$ of $L_{\min}$ in $L^{2}(\Omega )$, regularity results or sufficient criteria for self-adjointness, see also Section 4.3 for the second order case.

Corollary 4.15 *Let*$\left\{L^{2}{\left(\partial \Omega \right)}^{m},\Gamma_{0},\Gamma_{1}\right\}$*be the quasi-boundary triple from*Theorem 4.11*, and let*$\overset{˜}{\gamma }(\lambda )$*and*$\overset{˜}{M}(\lambda )$*,*$\lambda \in \rho (L_{D})$*, be the extended γ-field and Weyl function, respectively. Assume that*$L_{N}$*is self-adjoint, that*

$$\vartheta \subset \prod_{j=0}^{m-1}H^{-m_{j}-1/2}(\partial \Omega )\times \prod_{j=0}^{m-1}H^{-2m+m_{j}+1/2}(\partial \Omega )$$

*is a linear relation in*$\mathrm{ran}(\overset{˜}{D},\overset{˜}{N})$*and that the corresponding extension*

$$L_{\vartheta }:=L_{\max}↾\left\{f\in H_{L}^{0}(\Omega ):\vartheta \overset{˜}{D}f-\overset{˜}{N}f=0\right\}$$

*is closed in*$L^{2}(\Omega )$*. Then for all*$\lambda \in \rho (L_{D})$*the following assertions* (i)–(iv) *hold:*

(i) $\lambda \in \sigma_{p}(L_{\vartheta })$*if and only if*$0\in \sigma_{p}(\iota_{+}(\vartheta -\overset{˜}{M}(\lambda ))\iota_{-}^{-1})$*, in this case*
  $$\ker(L_{\vartheta }-\lambda )=\overset{˜}{\gamma }(\lambda )\ker\left(\vartheta -\overset{˜}{M}(\lambda )\right),$$
(ii) $\lambda \in \sigma_{c}(L_{\vartheta })$*if and only if*$0\in \sigma_{c}(\iota_{+}(\vartheta -\overset{˜}{M}(\lambda ))\iota_{-}^{-1})$*,*
(iii) $\lambda \in \sigma_{r}(L_{\vartheta })$*if and only if*$0\in \sigma_{r}(\iota_{+}(\vartheta -\overset{˜}{M}(\lambda ))\iota_{-}^{-1})$*,*
(iv) $\lambda \in \rho (L_{\vartheta })$*if and only if*$0\in \rho (\iota_{+}(\vartheta -\overset{˜}{M}(\lambda ))\iota_{-}^{-1})$*and*
  $${\left(L_{\vartheta }-\lambda \right)}^{-1}={\left(L_{D}-\lambda \right)}^{-1}+\overset{˜}{\gamma }(\lambda ){\left(\vartheta -\overset{˜}{M}(\lambda )\right)}^{-1}\overset{˜}{\gamma }{\left(\overset{¯}{\lambda }\right)}^{\prime }$$
  *holds for all*$\lambda \in \rho (L_{\vartheta })\cap \rho (L_{D})$*.*

### 4.3 Second order elliptic differential operators on smooth domains with compact boundary

In this section we pay particular attention to a special second order case which appears in the literature in different contexts, see, e.g., [10], [12], [13], [14], [42], [43], [44].

Let $\Omega \subset R^{n}$, n≥2, be a bounded or unbounded domain with a compact $C^{\infty }$-smooth boundary ∂*Ω* and consider the second order differential expression on *Ω* given by

$$L=-\sum_{j,\;k=1}^{n}\partial_{j}a_{jk}\partial_{k}+a$$

with coefficients $a_{jk}\in C^{\infty }(\bar{\Omega })$ such that $a_{jk}(x)=a_{kj}(x)$ for all $x\in \bar{\Omega }$ and j,k∈{1,…,n}, and $a\in L^{\infty }(\Omega )$ real. In the case that *Ω* is unbounded we also assume that the first partial derivatives of the functions $a_{jk}$ are bounded in *Ω*. Furthermore, the ellipticity condition $\sum_{j,\;k=1}^{n}a_{jk}(x)\xi_{j}\xi_{k}\geq c\sum_{k=1}^{n}\xi_{k}^{2}$ is assumed to hold for some c>0 and all $\xi \in R^{n}$ and $x\in \bar{\Omega }$. As in Section 4.2 we define the Hilbert spaces $H_{L}^{s}(\Omega )$ and inner products via (4.12) and (4.13), respectively. The minimal and maximal realization of the differential expression L are

$$A=L_{\min}=L↾H_{0}^{2}(\Omega )\;\text{and}\;A^{⁎}=L_{\max}=L↾H_{L}^{0}(\Omega ),$$

and we set $T:=L↾H^{2}(\Omega )$. The minimal operator *A* is a closed, densely defined, symmetric operator in $L^{2}(\Omega )$ with equal infinite deficiency indices. The Dirichlet and Neumann trace operator are defined by

$$\tau_{D}=f{↾}_{\partial \Omega }\;\text{and}\;\tau_{N}f=\sum_{j,k=1}^{n}a_{jk}n_{j}\partial_{k}f{↾}_{\partial \Omega },\;f\in C^{\infty }(\bar{\Omega }),$$

and extended by continuity to a surjective mapping ${\left(\tau_{D},\tau_{N}\right)}^{⊤}:H^{2}(\Omega )\to H^{3/2}(\partial \Omega )\times H^{1/2}(\partial \Omega )$; cf. [52]. Here $n={\left(n_{1},n_{2},\ldots ,n_{n}\right)}^{⊤}$ denotes the unit vector field pointing out of *Ω*.

The next theorem is a variant of Theorem 4.1 and Theorem 4.11 with $D=\tau_{D}$ and $N=-\tau_{N}$; cf. [12], [13]. We do not repeat the proof here and refer only to [16, Theorem 5] and [9, Theorem 7.1] for the self-adjointness of $L_{D}$ and $L_{N}$, respectively. As in the previous theorems the spaces $G_{0}$ and $G_{1}$ from Definition 2.7 turn out to be dense in $L^{2}(\partial \Omega )$, the *γ*-field coincides with a family of Poisson operators and the values of the Weyl function are (up to a minus sign) Dirichlet-to-Neumann maps.

Theorem 4.16 *Let*$T=L↾H^{2}(\Omega )$*and let*

$$\Gamma_{0},\Gamma_{1}:H^{2}(\Omega )\to L^{2}(\partial \Omega ),\;\Gamma_{0}f:=\tau_{D}f,\;\Gamma_{1}f:=-\tau_{N}f.$$

*Then*$\left\{L^{2}(\partial \Omega ),\Gamma_{0},\Gamma_{1}\right\}$*is a quasi-boundary triple for*$T\subset A^{⁎}=L_{\max}$*such that the minimal realization*$A=L_{\min}$*coincides with*T↾ker⁡Γ*and the following statements hold.*

(i) *The Dirichlet realization*$L_{D}$*and Neumann realization*$L_{N}$*correspond to*$\ker\Gamma_{0}$*and*$\ker\Gamma_{1}$*,*
  $$L_{D}:=T↾\ker\Gamma_{0}=L_{\max}↾\left\{f\in H^{2}(\Omega ):\tau_{D}f=0\right\},$$
  $$L_{N}:=T↾\ker\Gamma_{1}=L_{\max}↾\left\{f\in H^{2}(\Omega ):\tau_{N}f=0\right\},$$
  *respectively, and both operators are self-adjoint in*$L^{2}(\Omega )$*.*
(ii) *The spaces*
  $$G_{0}:=\mathrm{ran}(\Gamma_{0}↾\ker\Gamma_{1})=H^{3/2}(\partial \Omega ),$$
  $$G_{1}:=\mathrm{ran}(\Gamma_{1}↾\ker\Gamma_{0})=H^{1/2}(\partial \Omega ),$$
  *are dense in*$L^{2}(\partial \Omega )$*.*
(iii) *The values*$\gamma (\lambda ):L^{2}(\partial \Omega )⊃H^{3/2}(\partial \Omega )\to L^{2}(\Omega )$*of the γ-field are given by*
  $$\gamma (\lambda )\varphi =f,\;\varphi \in H^{3/2}(\partial \Omega ),\;\lambda \in \rho (L_{D}),$$
  *where*$f\in L^{2}(\Omega )$*is the unique solution of the boundary value problem*
  (4.22) $$(L-\lambda )f=0,\;\tau_{D}f=\varphi .$$
(iv) *The values*$M(\lambda ):L^{2}(\partial \Omega )⊃H^{3/2}(\partial \Omega )\to L^{2}(\partial \Omega )$*of the Weyl function are given by*
  $$M(\lambda )\varphi =-\tau_{N}f,\;\varphi \in H^{3/2}(\partial \Omega ),\;\lambda \in \rho (L_{D}),$$
  *where*f=γ(λ)φ*is the unique solution of*(4.22)*.*

Let $\left\{L^{2}(\partial \Omega ),\Gamma_{0},\Gamma_{1}\right\}$ be the quasi-boundary triple from Theorem 4.16. In the same way as in (4.19) and (4.20) we obtain that ${\left(\tau_{D},\tau_{N}\right)}^{⊤}$ admits a continuous extension to a mapping

$${\left({\overset{˜}{\tau }}_{D},{\overset{˜}{\tau }}_{N}\right)}^{⊤}:H_{L}^{0}(\Omega )\to H^{-1/2}(\partial \Omega )\times H^{-3/2}(\partial \Omega ),$$

where for all s∈[0,2] the restrictions

$${\overset{˜}{\tau }}_{D}↾H_{L}^{s}(\Omega ):H_{L}^{s}(\Omega )\to H^{s-1/2}(\partial \Omega ),$$

$${\overset{˜}{\tau }}_{N}↾H_{L}^{s}(\Omega ):H_{L}^{s}(\Omega )\to H^{s-3/2}(\partial \Omega ),$$

are continuous and surjective; cf. (4.21).

The quasi-boundary triples in the next proposition were first introduced in [11] on the domains $H^{2}(\Omega )$ and $H_{L}^{3/2}(\Omega )$. We note that the latter space coincides with the first order Beals space $B_{L}^{1}(\Omega )$, see [9].

Proposition 4.17 *Let* s∈[0,2] *,* $T_{s}:=L_{\max}↾H_{L}^{s}(\Omega )$ *, and let*

$$\Gamma_{0}^{s}:H_{L}^{s}(\Omega )\to H^{s-1/2}(\partial \Omega ),\;\Gamma_{0}^{s}f:={\overset{˜}{\tau }}_{D}f,$$

$$\Gamma_{1}^{s}:H_{L}^{s}(\Omega )\to H^{s-3/2}(\partial \Omega ),\;\Gamma_{1}^{s}f:=-{\overset{˜}{\tau }}_{N}f.$$

*Then the spaces*

$$G_{0}=\mathrm{ran}\;\left(\Gamma_{0}^{s}↾\ker\Gamma_{1}^{s}\right)=H^{3/2}(\partial \Omega ),$$

$$G_{1}=\mathrm{ran}\;\left(\Gamma_{1}^{s}↾\ker\Gamma_{0}^{s}\right)=H^{1/2}(\partial \Omega ),$$

*are dense in* $L^{2}(\partial \Omega )$ *and do not depend on s. Moreover, if* $s\in [\frac{3}{2},2]$ *then* $\mathrm{ran}\;\Gamma_{0}^{s}\subset L^{2}(\partial \Omega )$ *,* $\mathrm{ran}\;\Gamma_{1}^{s}\subset L^{2}(\partial \Omega )$ *, and* $\left\{L^{2}(\partial \Omega ),\Gamma_{0}^{s},\Gamma_{1}^{s}\right\}$ *is a quasi-boundary triple for* $T_{s}\subset A^{⁎}=L_{\max}$ *.*

Next we apply Theorem 2.12 to the quasi-boundary triple from Proposition 4.17. This boundary triple appears already in [39] in an implicit form, see also [10], [12], [17], [41], [53], [62]. Let $\iota_{\pm }:H^{\pm 1/2}(\partial \Omega )\to L^{2}(\partial \Omega )$ be a pair of isometric isomorphisms such that

$${\left(\iota_{-}x^{\prime },\iota_{+}x\right)}_{L^{2}(\partial \Omega )}={\langle x^{\prime },x\rangle }_{H^{-1/2}(\partial \Omega )\times H^{1/2}(\partial \Omega )}$$

holds for all $x\in H^{1/2}(\partial \Omega )$ and $x^{\prime }\in H^{-1/2}(\partial \Omega )$; cf. (2.15).

Corollary 4.18 *Let*$\eta \in \rho (L_{D})\cap R$*and define*${ϒ}_{0},{ϒ}_{1}:H_{L}^{0}(\Omega )\to L^{2}(\partial \Omega )$*by*

$${ϒ}_{0}f:=\iota_{-}{\overset{˜}{\tau }}_{D}f,\;{ϒ}_{1}f:=-\iota_{+}\tau_{N}f_{D},\;f=f_{D}+f_{\eta }\in \mathrm{dom}\;L_{D}∔N_{\eta }\left(A^{⁎}\right).$$

*Then*$\left\{L^{2}(\partial \Omega ),{ϒ}_{0},{ϒ}_{1}\right\}$*is an ordinary boundary triple for*$A^{⁎}=L_{\max}$*with*$A^{⁎}↾\ker{ϒ}_{0}=L_{D}$*and*

$$A^{⁎}↾\ker{ϒ}_{1}=L_{\min}\overset{˙}{+}\left\{{\left(f_{\eta },\eta f_{\eta }\right)}^{⊤}:Lf_{\eta }=\eta f_{\eta },\;f_{\eta }\in H_{L}^{0}(\Omega )\right\}.$$

As in Section 4.1 we apply Theorem 3.7 to the quasi-boundary triple from Theorem 4.16. The regularity statement can be proven in the same way as in Theorem 4.10.

Corollary 4.19 *Let*$\eta \in R\cap \rho (L_{D})\cap \rho (L_{N})$*and*$\overset{˜}{M}(\eta ):H^{-1/2}(\partial \Omega )\to H^{-3/2}(\partial \Omega )$*be the extended Dirichlet-to-Neumann map. Then the mapping*

$$\Theta \mapsto L_{\vartheta }=L_{\max}↾\left\{f\in H_{L}^{0}(\Omega ):\vartheta {\overset{˜}{\tau }}_{D}f+{\overset{˜}{\tau }}_{N}f=0\right\},\;\vartheta =\iota_{+}^{-1}\Theta \iota_{-}+\overset{˜}{M}(\eta ),$$

*establishes a bijective correspondence between all closed (symmetric, self-adjoint, (maximal) dissipative, (maximal) accumulative) linear relations Θ in*$L^{2}(\partial \Omega )$*and all closed (symmetric, self-adjoint, (maximal) dissipative, (maximal) accumulative, respectively) extensions*$L_{\vartheta }\subset L_{\max}$*of*$L_{\min}$*in*$L^{2}(\Omega )$*. Moreover, the following regularity result holds: For*s∈[0,2]

$$\mathrm{dom}\;\Theta \subset H^{s}(\partial \Omega )\;\text{implies}\;\mathrm{dom}\;L_{\vartheta }\subset H_{L}^{s}(\Omega ).$$

The next corollary is a consequence of Proposition 3.10 and Proposition 3.11. In item (i) we obtain an additional regularity statement.

Corollary 4.20 *Let*$\eta \in R\cap \rho (L_{D})\cap \rho (L_{N})$*and*$M(\eta ):H^{3/2}(\partial \Omega )\to H^{1/2}(\partial \Omega )$*be the Dirichlet-to-Neumann map from*Theorem 4.16(iv)*. Let ϑ be a symmetric linear operator in*$L^{2}(\partial \Omega )$*such that*

(4.23) $$H^{3/2}(\partial \Omega )\subset \mathrm{dom}\;\vartheta \;\text{and}\;\mathrm{ran}\;\left(\vartheta ↾H^{3/2}(\partial \Omega )\right)\subset H^{1/2}(\partial \Omega ),$$

*and assume that there exist*$c_{1}>0$*and*$c_{2}\in [0,1]$*such that*

$${\left\|\vartheta x\right\|}_{H^{1/2}(\partial \Omega )}\leq c_{1}{\left\|x\right\|}_{H^{-1/2}(\partial \Omega )}+c_{2}{\left\|M(\eta )x\right\|}_{H^{1/2}(\partial \Omega )},\;x\in H^{3/2}(\partial \Omega ).$$

*Then the following statements hold.*

(i) *If*$c_{2}\in [0,1)$*then*
  (4.24) $$L_{\vartheta }=L_{\max}↾\left\{f\in H_{L}^{0}(\Omega ):\vartheta {\overset{˜}{\tau }}_{D}f+{\overset{˜}{\tau }}_{N}f=0\right\}$$
  *is self-adjoint in*$L^{2}(\Omega )$*with regularity*$\mathrm{dom}\;L_{\vartheta }\subset H^{2}(\Omega )$*.*
(ii) *If*$c_{2}=1$*then*$L_{\vartheta }$*in*(4.24)*is essentially self-adjoint in*$L^{2}(\Omega )$*with regularity*$\mathrm{dom}\;L_{\vartheta }\subset H_{L}^{3/2}(\Omega )$*.*

Proof (i) The restriction $\theta :=\vartheta ↾H^{3/2}(\partial \Omega ):H^{3/2}(\partial \Omega )\to H^{1/2}(\partial \Omega )$ satisfies the assumptions in Proposition 3.10(iii) and hence we conclude that

$$L_{\theta }=L_{\max}↾\left\{f\in H^{2}(\Omega ):\theta \tau_{D}f+\tau_{N}f=0\right\}$$

is self-adjoint in $L^{2}(\Omega )$. As in Lemma 4.6 one verifies that the operator $L_{\vartheta }$ is a symmetric extension of the self-adjoint operator $L_{\theta }$ and hence both coincide.

(ii) follows in the same way as (i) from Proposition 3.11 and the reasoning in Lemma 4.6.  □

In the next example we consider a one parameter family $L_{\vartheta_{\alpha }}$ of extensions of $L_{\min}$ which correspond to $\vartheta_{\alpha }=\alpha \;M(\eta )$. It turns out that for α≠1 the extensions are self-adjoint and for α=1 essentially self-adjoint.

Example 4.21 Let $M(\eta ):H^{3/2}(\partial \Omega )\to H^{1/2}(\partial \Omega )$ be as in Corollary 4.20 and consider the symmetric operators $\vartheta_{\alpha }:=\alpha \;M(\eta )$, α∈R, in $L^{2}(\partial \Omega )$ with $\mathrm{dom}\;\vartheta_{\alpha }=H^{3/2}(\partial \Omega )$ and α∈R. Then according to Corollary 4.20 the extension

$$L_{\vartheta_{\alpha }}=L_{\max}↾\left\{f\in H_{L}^{0}(\Omega ):\vartheta_{\alpha }{\overset{˜}{\tau }}_{D}f+{\overset{˜}{\tau }}_{N}f=0\right\}=L_{\max}↾\left\{f\in H^{2}(\Omega ):\alpha M(\eta )\tau_{D}f+\tau_{N}f=0\right\}$$

in (4.24) is self-adjoint if |α|<1 and essentially self-adjoint if |α|=1. Here we have used ${\overset{˜}{\tau }}_{D}f=\tau_{D}f$ and ${\overset{˜}{\tau }}_{N}f=\tau_{N}f$ for $f\in H^{2}(\Omega )$. It follows in the same way as in Example 3.9 that

$$L_{\vartheta_{1}}=L_{\max}↾\left\{f\in H^{2}(\Omega ):M(\eta )\tau_{D}f+\tau_{N}f=0\right\}=L_{\min}\overset{˙}{+}\left\{{\left(f_{\eta },\eta f_{\eta }\right)}^{⊤}:Lf_{\eta }=\eta f_{\eta },\;f_{\eta }\in H^{2}(\Omega )\right\}.$$

We also remark that

$${\bar{L}}_{\vartheta_{1}}=L_{\min}\overset{˙}{+}\left\{{\left(f_{\eta },\eta f_{\eta }\right)}^{⊤}:Lf_{\eta }=\eta f_{\eta },\;f_{\eta }\in H_{L}^{0}(\Omega )\right\}=L_{\min}\overset{˙}{+}{\overset{ˆ}{N}}_{\eta }\left(A^{⁎}\right).$$

For α≤−1 and α>1 we make use of Corollary 3.5. For this we set

$$\Theta_{\alpha }:=\iota_{+}\left(\vartheta_{\alpha }-M(\eta )\right)\iota_{-}^{-1}=(\alpha -1)\iota_{+}M(\eta )\iota_{-}^{-1},\;\mathrm{dom}\;\Theta_{\alpha }=H^{2}(\partial \Omega ),$$

and note that the operators $\Theta_{\alpha }$ are self-adjoint in $L^{2}(\partial \Omega )$. Hence Corollary 3.5 yields that for α≤−1 and α>1 the extensions $L_{\vartheta_{\alpha }}$ are self-adjoint in $L^{2}(\Omega )$.

The following example is related to the case α=1 in the above example. It contains an observation which can also be interpreted from a slightly more abstract point of view. Namely, Example 4.22 shows that there exists a quasi-boundary triple $\left\{G,\Gamma_{0},\Gamma_{1}\right\}$ for $T\subset A^{⁎}$ and a self-adjoint relation *ϑ* in G with ϑ⊂ranΓ such that the extension $A_{\vartheta }:=T↾\{f\in \mathrm{dom}\;T:\Gamma f\in \vartheta \}$ is not self-adjoint in H; cf. Section 3.1.

Example 4.22 Let $\left\{L^{2}(\partial \Omega ),\Gamma_{0}^{s},\Gamma_{1}^{s}\right\}$ be the quasi-boundary triple from Proposition 4.17 for $s=\frac{3}{2}$ defined on the domain of

$$T_{3/2}=L_{\max}↾H_{L}^{3/2}(\Omega )\subset A^{⁎}.$$

The values of the corresponding Weyl function $M_{3/2}$ are mappings from $H^{1}(\partial \Omega )$ to $L^{2}(\partial \Omega )$. For $\eta \in R\cap \rho (L_{D})\cap \rho (L_{N})$ set $\vartheta :=M_{3/2}(\eta )$ with $\mathrm{dom}\;\vartheta =H^{1}(\partial \Omega )$. Then *ϑ* is a bijective symmetric operator in $L^{2}(\partial \Omega )$ and hence self-adjoint. As in Example 3.9 one verifies that the corresponding extension $L_{\vartheta }$ is given by

$$L_{\vartheta }=L_{\max}↾\left\{f\in H_{L}^{3/2}(\Omega ):\vartheta {\overset{˜}{\tau }}_{D}f+{\overset{˜}{\tau }}_{N}f=0\right\}=L_{\min}∔{\overset{ˆ}{N}}_{\eta }(T_{3/2})$$

and that ${\bar{L}}_{\vartheta }=L_{\min}∔{\overset{ˆ}{N}}_{\eta }(A^{⁎})=A^{⁎}↾\ker{ϒ}_{0}$ holds; here ${ϒ}_{0}$ is the boundary mapping from Corollary 4.18. Therefore $L_{\vartheta }$ is a proper restriction of the self-adjoint extension ${\bar{L}}_{\vartheta }$ and it follows, in particular, that $L_{\vartheta }$ is essentially self-adjoint, but not self-adjoint in $L^{2}(\Omega )$.

Proposition 3.10 together with well known compact embedding properties of Sobolev spaces yield some simple sufficient conditions for self-adjoint realizations of L.

Proposition 4.23 *Let ϑ be a symmetric operator in* $L^{2}(\Omega )$ *such that* (4.23) *holds, and assume that ϑ is continuous as a mapping from* $H^{3/2-\delta_{1}}(\partial \Omega )$ *to* $H^{1/2+\delta_{2}}(\partial \Omega )$ *, where* $\delta_{1}\in [0,\frac{3}{2}]$ *,* $\delta_{2}\geq 0$ *and* $\delta_{1}+\delta_{2}>0$ *. Then*

$$L_{\vartheta }=L_{\max}↾\left\{f\in H_{L}^{0}(\Omega ):\vartheta {\overset{˜}{\tau }}_{D}f+{\overset{˜}{\tau }}_{N}f=0\right\}$$

*is self-adjoint in* $L^{2}(\Omega )$ *with regularity* $\mathrm{dom}\;L_{\vartheta }\subset H^{2}(\Omega )$ *.*

Proof Observe that at least one of the embeddings $H^{3/2}(\partial \Omega )↪H^{3/2-\delta_{1}}(\partial \Omega )$ or $H^{1/2+\delta_{2}}(\partial \Omega )↪H^{1/2}(\partial \Omega )$ is compact; cf. [76, Theorem 7.10]. Hence we conclude that $\theta :=\vartheta ↾H^{3/2}(\partial \Omega ):H^{3/2}(\partial \Omega )\to H^{1/2}(\partial \Omega )$ is a compact operator. Therefore Proposition 3.10(i) yields that $L_{\theta }$ is self-adjoint in $L^{2}(\Omega )$ with regularity $\mathrm{dom}\;L_{\theta }\subset H^{2}(\Omega )$; cf. the proof of Corollary 4.20. It follows as in Lemma 4.6 that $L_{\vartheta }$ is a symmetric extension of the self-adjoint operator $L_{\theta }$ and hence both operators $L_{\vartheta }$ and $L_{\theta }$ coincide.  □

Finally we illustrate Proposition 4.23 with a simple example.

Example 4.24 Let $0<\varepsilon \leq \frac{3}{2}$ and assume that

$$\alpha \in M\left(H^{3/2}(\partial \Omega ),H^{1/2+\varepsilon }(\partial \Omega )\right)\;\text{or}\;\alpha \in M\left(H^{3/2-\varepsilon }(\partial \Omega ),H^{1/2}(\partial \Omega )\right),$$

where M(⋅,⋅) denotes the space of all pointwise multipliers; cf. [56], [71]. Then it follows from Proposition 4.23 that

$$L_{\alpha }=L_{\max}↾\left\{f\in H_{L}^{0}(\Omega ):\alpha \cdot {\overset{˜}{\tau }}_{D}f+{\overset{˜}{\tau }}_{N}f=0\right\}$$

is self-adjoint in $L^{2}(\Omega )$ with regularity $\mathrm{dom}\;L_{\alpha }\subset H^{2}(\Omega )$. In particular, since $C^{r}(\partial \Omega )\subset M(H^{1/2}(\partial \Omega ),H^{1/2}(\partial \Omega ))$ for $r\in (\frac{1}{2},\;1)$ the assertion holds for all $\alpha \in C^{r}(\partial \Omega )$, $r\in (\frac{1}{2},\;1)$.

## Footnotes

1 We emphasize that $G_{0}$ and $G_{1}$ in Definition 2.7 do, in general, not coincide with the spaces $G_{0}=\mathrm{ran}\;\Gamma_{0}$ and $G_{1}=\mathrm{ran}\;\Gamma_{1}$; this notation was used in [11], [12]. The symbols $G_{0}$ and $G_{1}$ will not be used in the present paper.

2 Here and in the following the expression $\Gamma_{1}-\Theta \Gamma_{0}$ is understood in the sense of linear relations if *Θ* is a linear relation, that is, $\Theta \Gamma_{0}$ is the product of the relation *Θ* with (the graph of the mapping) $\Gamma_{0}$ and the sum of $\Gamma_{1}$ and $-\Theta \Gamma_{0}$ is in sense of linear relations. We refer the reader to [6], [23], [29], [30], [46] for more details on linear relations.
